# Simulating Bacterial Membrane Models at the Atomistic Level: A Force Field Comparison

**DOI:** 10.1021/acs.jctc.4c00204

**Published:** 2024-09-03

**Authors:** Alexandre Blanco-González, Anika Wurl, Tiago Mendes Ferreira, Ángel Piñeiro, Rebeca Garcia-Fandino

**Affiliations:** † Facultad de Física, 223355 University of Santiago de Compostela (USC), 15782 Santiago de Compostela, Spain; ‡ Singular Research Centre in Chemical Biology and Molecular Materials, (CIQUS), Organic Chemistry Department, University of Santiago de Compostela (USC), 15782 Santiago de Compostela, Spain; § MD.USE Innovations S.L, Edificio Emprendia, 15782 Santiago de Compostela, Spain; ∥ Institute of Physics, Faculty of Natural Sciences II, Betty-Heimann-Str. 7, 06120 Halle/Saale, Germany

## Abstract

Molecular dynamics (MD) simulations are currently an indispensable tool to understand both the dynamic and nanoscale organization of cell membrane models. A large number of quantitative parameters can be extracted from these simulations, but their reliability is determined by the quality of the employed force field and the simulation parameters. Much of the work on parametrizing and optimizing force fields for biomembrane modeling has been focused on homogeneous bilayers with a single phospholipid type. However, these may not perform effectively or could even be unsuitable for lipid mixtures commonly employed in membrane models. This work aims to fill this gap by comparing MD simulation results of several bacterial membrane models using different force fields and simulation parameters, namely, CHARMM36, Slipids, and GROMOS-CKP. Furthermore, the hydrogen isotope exchange (HIE) method, combined with GROMOS-CKP (GROMOS-H2Q), was also tested to check for the impact of this acceleration strategy on the performance of the force field. A common set of simulation parameters was employed for all of the force fields in addition to those corresponding to the original parametrization of each of them. Furthermore, new experimental order parameter values determined from NMR of several lipid mixtures are also reported to compare them with those determined from MD simulations. Our results reveal that most of the calculated physical properties of bacterial membrane models from MD simulations are substantially force field and lipid composition dependent. Some lipid mixtures exhibit nearly ideal behaviors, while the interaction of different lipid types in other mixtures is highly synergistic. None of the employed force fields seem to be clearly superior to the other three, each having its own strengths and weaknesses. Slipids are notably effective at replicating the order parameters for all acyl chains, including those in lipid mixtures, but they offer the least accurate results for headgroup parameters. Conversely, CHARMM provides almost perfect estimates for the order parameters of the headgroups but tends to overestimate those of the lipid tails. The GROMOS parametrizations deliver reasonable order parameters for entire lipid molecules, including multicomponent bilayers, although they do not reach the accuracy of Slipids for tails or CHARMM for headgroups. Importantly, GROMOS-H2Q stands out for its computational efficiency, being at least 3 times faster than GROMOS, which is already faster than both CHARMM and Slipids. In turn, GROMOS-H2Q yields much higher compressibilities compared to all other parametrizations.

## Graphical abstract

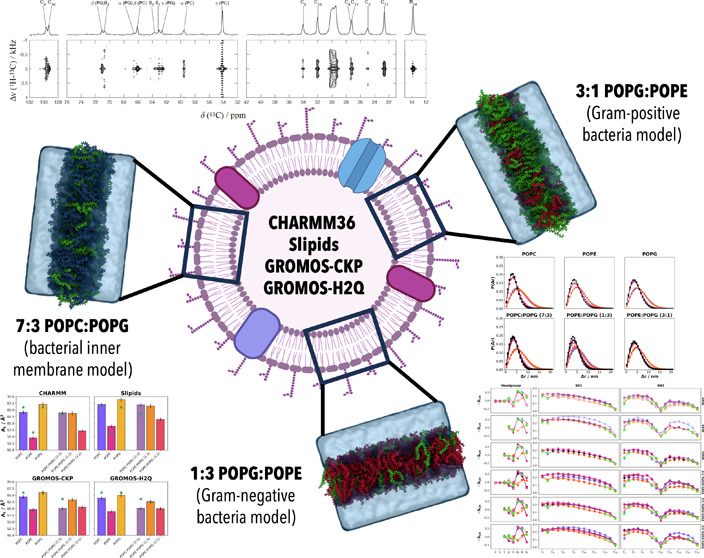

## 1 Introduction

Only 90 years after the introduction of penicillin and still immersed in a pandemic era, antimicrobial resistance (AMR) is threatening the ability of modern medicine to combat infectious diseases.^1^ We are now facing the possibility of a future without effective antimicrobial drugs for some infections, where surgical operations and treatments, such as cancer chemotherapy and organ transplants, could become scarily dangerous. The emergence of new bacteria and viruses and the persistent failure to develop or discover new antibiotics together with the overuse and misuse of antimicrobials are leading to an increasing antimicrobial resistance.^2^ The appearance of this resistance is a natural biological phenomenon and is an inevitable consequence of the continued use of antibiotics. This circumstance is accelerating at an alarming rate and has reached a potentially dangerous stage.^3,4^ That is why AMR has been declared as one of the global public health threats facing humanity.^5^

The interplay of bacteria with their surrounding ambiance is mediated, directly or indirectly, through the membranes that compose their cell envelopes. There is a large difference in the lipid composition of bacterial and mammalian cytoplasmic membranes, which can be used to specifically recognize pathogenic agents.^6^ Targeting the membrane is a promising strategy to develop novel antiresistance or fully resistance-free antimicrobials. The primary phospholipid components of bacterial cytoplasmic membranes are phosphatidylethanolamine (PE) and phosphatidylglycerol (PG). While PE is also present in significant proportions in eukaryotic cells, the concentration of PG is markedly lower in animal cell membranes, highlighting a key distinction.^7,8^ In contrast, the major lipid component of the animal cell membrane is phosphatidylcholine (PC), which is rarely present in the bacterial membrane.^9^ This difference in lipid composition between mammalian and bacterial membranes has led to the use of zwitterionic phospholipids or mixed phospholipid-charged bilayers as a good model for eukaryotic or bacterial membranes, respectively.^10,11^

Building on the understanding of the distinct lipid compositions between bacterial and mammalian membranes, an innovative approach emerges in the fight against AMR. This approach leverages the interconnection of lipid alterations to redefine treatment strategies, focusing on targeting cellular membranes to combat bacterial infections effectively. By identifying and exploiting the physical, chemical, and mechanical vulnerabilities of bacterial cells, new interventions can be developed to specifically address altered membranes in pathogenic bacteria. For example, the recognition of pathogenic agents by their membrane lipid composition has been exploited for millions of years by membrane-targeted endogenous therapeutic peptides (ETPs), which belong to the first defense barrier of the immune system.^12^ These peptides act directly on lipid cell membranes,^13^ without the need for specific membrane receptors, thus hindering the development of resistance mechanisms. Due to their cationic character, antimicrobial peptides have a preference for anionic membranes, typically presented by pathogens such as bacteria. The potential of creating molecules that mimic the action of ETPs presents an exciting opportunity for developing new treatments that specifically target and destroy bacteria with abnormal lipid profiles. Furthermore, the selective targeting and disruption of pathological bacterial membranes through nonchemical methods, such as focused ultrasonic wave pulses, open up new therapeutic interventions.^14^ By utilizing the mechanical and physicochemical properties of bacterial membranes, it could be possible to selectively eliminate bacteria while leaving healthy mammal cells unharmed. This approach offers significant advantages, including localized treatment with minimal side effects and precise delivery that minimizes the impact on healthy tissues.

Understanding the structural and functional intricacies of bacterial membranes is vital not only for comprehending the penetration and action of antimicrobial agents but also for exploring how mechanical disturbances can be strategically applied to selectively target and destroy pathogenic cells.^15^ Despite the advanced and rapid developments in wet-lab protocols, the intricate composition of bacterial membranes results in a varied structure that poses challenges for molecular-level experimental investigations. Achieving the deep level of detail needed to fully comprehend these interactions remains a challenge for many of today’s experimental methods. Over the past years, the use of computational modeling, especially molecular dynamics (MD) simulations, has emerged as a valuable method to uncover crucial details regarding the structural details of bacterial cell walls.^16^ In this regard, MD simulations may provide the necessary bridges to achieve a better understanding of membrane-antimicrobial agent interaction processes. One of the most exciting advances in this scope has been the development of molecular-level models that incorporate details of the nonprotein constituents. Nowadays, computational studies of phospholipid mixtures are routine.^17,18^

While MD simulations can provide invaluable detailed structural and dynamical information about the studied system, they rely on the quality of the employed force field. During the last few years, a lot of effort has been dedicated to parametrizing and optimizing force fields for biomembrane modeling.^19–25^ However, most of the work has been done for homogeneous bilayers composed of a single phospholipid type. Such parameters may not work optimally or even fail when they are employed in more accurate membrane models described by complex inhomogeneous systems, including lipid membranes, in contact with proteins, DNA, nanoparticles, etc., or simply lipid mixtures. No comparative studies of force fields with computational models typically used for bacterial membranes have been published to date, although hybrid membranes with several different lipids have been studied by different theoretical and experimental methods.^26–29^ This work intends to fill this gap using three different classical force fields: CHARMM36,^30^ Slipids,^31–33^ and GROMOS-CKP^34^ with two sets of simulation parameters for the first two force fields. Furthermore, the hydrogen isotope exchange (HIE) method, combined with GROMOS-CKP (GROMOS-H2Q), was also tested to explore how the increase of the time step by a factor of 3 upon slowing down the fastest degrees of freedom affects the results. This approach proved to reproduce well the structural and dynamical properties of other systems like cyclodextrins^35^ or cyclic peptide nanotubes^36^ while reducing the required computational resources to one-third with respect to the standard simulations, but it has not been tested yet for lipid membranes.

In this work, different combinations of 1-palmitoyl-2-oleoyl-*sn*-glycero-3-phosphatidylethanolamine (POPE) and 1-palmitoyl-2-oleoyl-*sn*-glycero-3-phosphatidylglycerol (POPG) are used as simple models for the bacterial membrane since they are known to have a significant presence in the Gram-negative and Gram-positive bacterial cytosolic membranes. Although the lipid composition varies quite significantly among pathogens, typically, Gram-negative bacteria have a higher PE content and Gram-positive bacteria have a higher PG content.^37^ Thus, to model the membranes of the Gram-positive bacterial membrane, we simulated a 3:1 ratio of POPG/POPE, while a 1:3 ratio of POPG/POPE was used to model the inner membranes of Gram-negative bacteria.^38^ A mixture with a 7:3 molar ratio of zwitterionic POPC to anionic POPG lipids mimicking the bacterial inner membrane was also included in the study.^39^ While this mixture is not a canonical model of the bacterial membrane, it is taken in our work as a simplification of a possible composition present in some strains.^40^ Finally, eukaryotic membranes are mimicked by using 1-palmitoyl-2-oleoyl-*sn*-glycero-3-phosphocholine (POPC), to model the outer leaflet of the mammalian membrane (Figure 1). It is worth mentioning that we use just PO tails, a common choice in the literature due to their well-documented behavior in computational models. It is well-known, however, that bacterial lipids exhibit a broader diversity in acyl chain composition, such as the inclusion of cyclopropane moieties, which can replace double bonds under specific growth conditions^41^ and the presence of cardiolipin, particularly significant in bacterial and mitochondrial membranes for its role in membrane stability and function.^42^ Additionally, bacterial membranes often contain lipids with various headgroups and unsaturation levels, which can significantly alter membrane properties such as fluidity, curvature, and phase behavior.^40^

**Figure 1:**
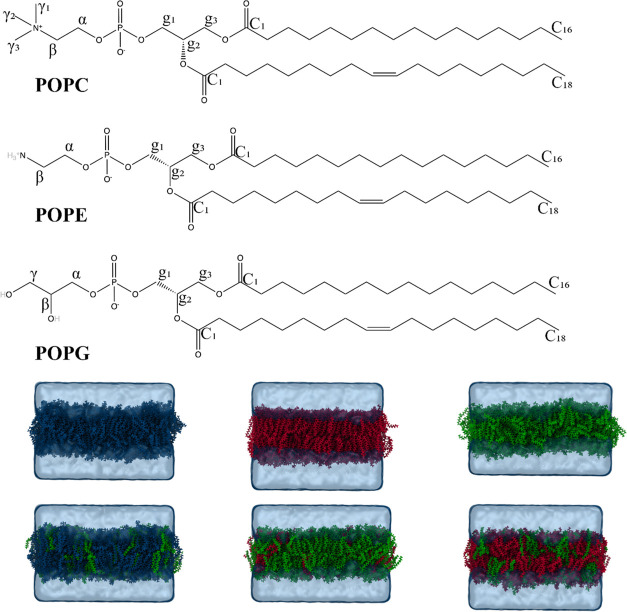
Chemical structures of POPC, POPE, and POPG with key atomic nomenclatures are shown above. 3D snapshots of six membrane compositions at *t* = 500 ns, using CHARMM: Pure POPC (blue), POPE (red), and POPG (green) membranes and bacterial model membranes: 7:3 POPC:POPG (bacterial inner membrane model), 1:3 POPG:POPE (Gram-negative bacterial model), and 3:1 POPG:POPE (Gram-positive bacterial model) are shown at the bottom of the figure. The results obtained for the rest of the force fields (Slipids, GROMOS-CKP, GROMOS-H2Q, as well as CHARMM-O and Slipids-O) are represented in Figures S1–S6.

A comprehensive comparison of several equilibrium physical properties of bacterial membrane models from MD simulations was conducted for the different studied force fields. This analysis included area per lipid, bilayer thickness, hydration levels within the bilayer’s inner region, hydrogen bonding patterns, radial distribution function, lateral density, compressibilities, and two-dimensional (2D)-density profiles. Additionally, we also present a calculation of C–H order parameters from the different simulations and their comparison with experimental order parameters obtained by using dipolar recoupling solid-state nuclear magnetic resonance (NMR), namely, R-type proton detected local field (R-PDLF) experiments as described previously.^43–45^ Our study is expected to provide valuable insights into the dynamics and structural features of bacterial membranes. This knowledge will serve as a reference for selecting appropriate force fields in simulations of heterogeneous membranes, ultimately enabling optimization of the current topological parameters for lipids.

## 2 Methods

### 2.1 Computational Methods

Three different force fields, namely, CHARMM36,^30^ Slipids,^31–33^ and GROMOS-CKP^34^ were used for the simulation of six lipid membranes: POPC, POPE, POPG, POPC/POPG (7:3), POPE/POPG (1:3), and POPE/POPG (3:1). Additionally, the HIE method with H2Q,^35,36^ which quadruples the mass of the polar hydrogens to mimic Quadium, combined with GROMOS-CKP, was also tested for all of the membrane compositions. This force field parametrization, referred to as GROMOS-H2Q along the document, was employed with a time step of 6 fs to integrate the motion equations.

All of the membranes were built with CHARMM-GUI Input Generator^46–48^ and simulated using the GROMACS 2018.3 simulation package.^49^ Each of them consisted of 500 lipids (250 per leaflet) solvated by 25000 TIP3P water molecules in a box of ∼125 × 125 × 96 Å^3^. For the systems containing charged lipids, enough Na^+^ ions were added until charge neutrality was reached, using the standard available ion parameters for each force field. Prior to the simulation, all systems were minimized using the steepest descent method for 1000 steps. An NPT ensemble was employed at 1 bar and at 310 K using a semi-isotropic Parrinello–Rahman barostat,^50^ using a coupling constant of 1 ps and a V-rescale thermostat^51^ with a coupling constant of 0.1 ps. The LINCS algorithm was employed to remove bond vibrations.^52^ Electrostatic interactions were calculated using the PME method with a cutoff of 1.0 nm and a grid spacing of 0.12 nm.^53^ Van der Waals interactions were calculated using a 1.0 nm radius cutoff. These simulation parameters were employed for all of the force fields even when they did not correspond exactly to those employed for their original parametrization. From a practical point of view, a common set of simulation parameters is convenient to facilitate the comparison between force fields. It is also worth mentioning that longer cutoffs do not necessarily imply more realistic MD simulations, as it has been shown by increasing the short-range cutoff in CHARMM simulations, which takes DPPC bilayers to a gel phase under conditions where it should be in a fluid phase.^22^ Additionally, all of the systems were also simulated using CHARMM and Slipids with the parameter values that were originally used for their parametrization, labeled CHARMM-O and Slipids-O in this work. The main differences between the common parameters that we used for all of the force fields and the original CHARMM parameters were that the latter used constraints for just the bonds involving H atoms, a force-switch modifier for the van der Waals interactions with a switch radius of 1.0 nm, have longer cutoff radii of 1.2 nm for both electrostatics and Lennard-Jones interactions, as well as the neighbor list, and do not apply a dispersion correction scheme for either energy or pressure. For the case of Slipids-O, the differences with the common simulation parameter file employed were that the former have longer radii of 1.4 nm for the neighbor list and Coulombic and Lennard-Jones interactions, with a potential-shift-Verlet modifier in the van der Waals forces. All simulations were performed for 500 ns and analyzed just over the last 100 ns of the trajectory.

#### 2.1.1 Analysis of the Trajectories

VMD^54^ was employed to generate snapshots and animations from trajectories. GROMACS commands were employed for some standard analyses. Other analyses were performed using specifically developed Python scripts, based mainly on the MDAnalysis,^55,56^ NumPy,^57^ and Matplotlib^58^ libraries. The first 400 ns of each trajectory were discarded for the calculation of all of the average properties to ensure a proper equilibration for the analyses. All averages are taken using the *NumPy.mean* function and standard deviations (error bars in all cases) are taken using *NumPy.std* with N–1 Delta degrees of freedom to get an unbiased estimator of the variance in the limit of infinite population.

The area per lipid was calculated by dividing the *XY* simulation box area by the number of lipids present in one leaflet of the bilayer.

The thickness of the bilayers was evaluated as the distance between the average positions of the phosphorus (P) atoms in each of the two leaflets of a bilayer (P–P distance).

Lateral densities were calculated with GROMACS and represented by using Python scripts. The following groups are used for this calculation: Headgroup (from the upper end of the lipid to phosphorus atom, included), glycerol (from the oxygen bonded to the phosphorus to the ester oxygen, included), and tails (from the carbonyl ester to the end of the tail). In the all-atom force fields, the polar hydrogens were ignored to make the results comparable to those obtained with GROMOS parametrizations. The errors for the lateral densities were calculated using the block average method: lateral density was calculated in intervals of 10 ns for the last 100 ns of the trajectory. The number of water molecules located within the bilayer’s inner region (defined as the area situated between the glycerol lateral density peaks) was derived by converting the lateral density from kg/m^3^ to the number of water molecules along the *Z*-axis in #molecules/nm and then integrating this water curve along the *Z*-axis within the region between the glycerol peaks.

The 2D densities (in *XY*) were calculated for each leaflet using MDAnalysis in intervals of 10 ns, using bins with a width of 0.1 × 0.1 nm^2^. The results are represented with Matplotlib.

Radial distribution functions (RDF) between phosphate groups (PO4) were calculated with GROMACS considering the center of mass of the PO4 group from the different lipids.

Hydrogen bonds (H-bonds) between the different groups (headgroups and glycerol) of each lipid and the neighbors along the last 100 ns of the trajectory are calculated using the *h-bond* tool of the GROMACS package. The obtained values were normalized to account for differences in molecule concentration and bonding capabilities. The normalization of the observed H-bonds between two molecule types (*X* and *Y*) is calculated as

$$N_{\mathrm{norm}}\left(X,Y\right)=\frac{N_{\mathrm{obs}}\left(X,Y\right)}{N_{\max}\cdot \left[P\left(X\right)+P\left(Y\right)\right]}$$

where *N*_obs_(*X,Y*) is the observed number of H-bonds between types *X* and *Y* and *N*_max_ represents the total number of H-bonds that could theoretically exist within the entire system. The probability of molecule *X* or *Y* forming a H-bond, denoted as *P*(*X*) or *P*(*Y*), respectively, is derived from their relative abundances and bonding capabilities. For instance, in a simulation with various molecule types, let us assume that we have *n*_*i*_ molecules of molecule *i*, each capable of forming χ_*i*_ H-bonds, thus

$$N_{\max}=\sum_{i}\chi_{i}\cdot n_{i}$$

where *i* iterates over the total number of different compounds of the system. The probability for compound *Y* can be defined as

$$P\left(Y\right)=\frac{\chi_{Y}\cdot n_{Y}}{N_{\max}}$$

This approach involved setting maximum H-bond counts for different atomic species: neutral oxygen atoms can form two H-bonds based on two lone electron pairs, negatively charged oxygen atoms up to three due to an extra lone pair, and each polar Hydrogen atom can form one H-bond as a donor. The groups for H-bond computation were categorized into three types: headgroup, glycerol, and solvent, encompassing different molecular structures. This approach yields a dimensionless metric, effectively correcting for biases related to the diverse H-bonding capacities and varying molecular quantities across different species.

The C–H bond order parameters were calculated directly from the carbon and hydrogen positions using the following formula

$$S_{\mathrm{CH}}=\frac{1}{2}\left\langle 3\cdot \cos^{2}\theta -1\right\rangle$$

where θ is the angle between the C–H bond and the membrane normal (taken to align with *z*, with bilayer periodicity in the *xy*-plane). Angular brackets denote the average over all sampled configurations.

Diffusion coefficients were calculated from the displacement distributions in different windows of 2, 5, and 10 ns, using the two-dimensional random walk equation as previously described.^59^ Finally, area compressibility modulus values of the lipid bilayers (*K*_A_) were determined following the method proposed by Feller and Pastor.^60^

### 2.2 Experimental Methods

#### 2.2.1 *S*_CH_ Order Parameters

The C–H order parameters (*S*_CH_) were determined through a solid-state nuclear magnetic resonance (NMR) technique utilizing dipolar recoupling, specifically through R-type proton detected local field (R-PDLF) experiments as previously detailed.^43–45^ POPC, POPE, and POPG were all purchased from Avanti Polar Lipids with more than 99% purity in the form of lipid powders. All chemicals have a natural abundance of isotopes. The single-component lipid membrane systems were prepared by simply mixing about 20 mg of lipid powder with an amount of water corresponding to the same hydration level of the simulated systems. The mixing with water was done inside an Eppendorf vial with a volume of 0.5 mL using a thin rod to mix the system and then by repeatedly and alternately centrifuging and mixing it until a homogeneous system was attained. For the membranes with lipid mixtures, first, the lipids were mixed in an organic solvent (chloroform/methanol 2:1) in a glass vial, and then, a lipid film was obtained by solvent evaporation under a nitrogen gentle stream at a temperature slightly below the solvent boiling point using an external water bath under sonication. The resulting film was then hydrated, as described above. The identification of the different peaks was based on previous assignments.^43,44,61^ All of the details for the setup of the R-PDLF experiments used and for the spectral analysis leading to the determination of the C–H order parameters are given in the Supporting Information (Figures S23–S30).

## 3 Results and Discussion

To thoroughly assess the equilibration process of lipid bilayers, a comprehensive evaluation over the 500 ns long MD trajectories was conducted. This evaluation encompassed a detailed recording of time profiles for a variety of critical parameters, each offering insights into different aspects of bilayer behavior and properties. These parameters included the area per lipid (*A*_L_), bilayer thickness, hydration levels within the bilayer’s inner region, H-bonding patterns, and radial distribution functions. Additionally, the study incorporated the lateral density analysis, and 2D-density (in *XY*) values were calculated for each leaflet. Moreover, the C–H order parameters were calculated from the simulations and compared to the experimental values determined for accessing the validity of the molecular structures obtained.

The stabilization and consistent behavior of the parameters over time were crucial for meaningful analysis of the MD trajectory, providing a robust foundation for understanding the complex dynamics and structural characteristics of lipid bilayers. This comprehensive approach allowed for an in-depth exploration of the lipid bilayer properties, shedding light on the nuances of membrane modeling and simulation accuracy.

Figure 1 showcases the final structures of all simulation systems after 500 ns, featuring 3D snapshots of six membrane compositions. These include pure POPC, POPE, and POPG membranes as well as bacterial model membranes: 7:3 POPC/POPG (representing the bacterial inner membrane model), 1:3 POPG/POPE (for the Gram-negative bacterial model), and 3:1 POPG/POPE (representing the Gram-positive bacterial model) using the CHARMM force field. The results obtained for the other force fields, namely, CHARMM-O, Slipids, Slipids-O, GROMOS-CKP, and GROMOS-H2Q, are represented in supplementary figures (Figures S1–S6).

### 3.1 Area per Lipid

Area per lipid (*A*_L_) is a significant property frequently used in the validation of lipid force field parameters. Yet, discrepancies often arise when comparing simulations to experimental data, stemming in part from the indirect nature of experimental *A*_L_ measurements.^62,63^ Whereas early studies using very small systems suggested that methodological differences were important, recent work using more realistic systems and more appropriate cutoffs has shown that *A*_L_ appears relatively insensitive to methodological aspects of simulations such as the treatment of the long-range electrostatic interactions (Ewald-summation methods, reaction-field correction, shift function or straight cutoff).^64^ Overall, the uncertainty in the experimental value of *A*_L_ means that this parameter by itself is a poor indicator of the quality of a force field even though it is in principle an important property that is worth reproducing. Nevertheless, the area per molecule is a very simple parameter to be calculated and analyzed that enables a straightforward comparison between the outcomes of the distinct simulations.

Examining the average *A*_L_ for the pure membranes (POPC, POPE, and POPG) over the last 100 ns of the simulations across various force fields reveals interesting patterns (Figures 2, S7 and Table S1). For the POPC lipid, the area values are quite consistent across the different force fields, with the highest area observed in Slipids at 67.11 ± 0.78 Å^2^ and the lowest in GROMOS-H2Q at 63.94 ± 0.46 Å^2^. The variation, though not drastic, emphasizes subtle differences in the lipid representation or interactions in each force field. In any case, the values agree reasonably well with the experimental results obtained at a similar temperature (66.0 Å^2^ at 310 K, though this value is not directly comparable as it was measured under slightly different conditions: 150 mM NaCl),^65^ especially for Slipids (67.11 ± 0.78 Å^2^) and Slipids-O (65.72 ± 0.80 Å^2^), also accordingly with our previous results.^66^ As one would expect, and in agreement with the experimental value (56.6 Å^2^ at 303K),^67^ POPE presents a lower *A*_L_ in all of the simulated force fields, although the biggest differences are observed for CHARMM (54.55 ± 0.64 Å^2^ versus 57.16 ± 0.59 Å^2^ in CHARMM-O, 58.97 ± 0.63 Å^2^ in Slipids, 58.03 ± 0.73 Å^2^ in Slipids-O, 59.64 ± 0.45 Å^2^ in GROMOS-CKP, and 58.92 ± 0.51 Å^2^ in GROMOS-H2Q). These differences underscore the importance of force field choice and also the simulation parameters, especially when modeling specific lipid types like POPE. The difference between the result obtained for CHARMM using the common simulation parameters and those corresponding to the original parametrization of this force field is remarkable. For POPG, which lacks substantial experimental data for validation, Slipids tops the chart with 68.88 ± 0.94 Å^2^, and GROMOS-H2Q occupies the lower end with 65.03 ± 0.61 Å^2^. The differences again arise from distinct parameters and interactions defined by each force field. Differences between simulation parameters (CHARMM vs CHARMM-O and Slipids vs Slipids-O) are less important for this lipid than the differences between these two forces and the two GROMOS variants.

**Figure 2:**
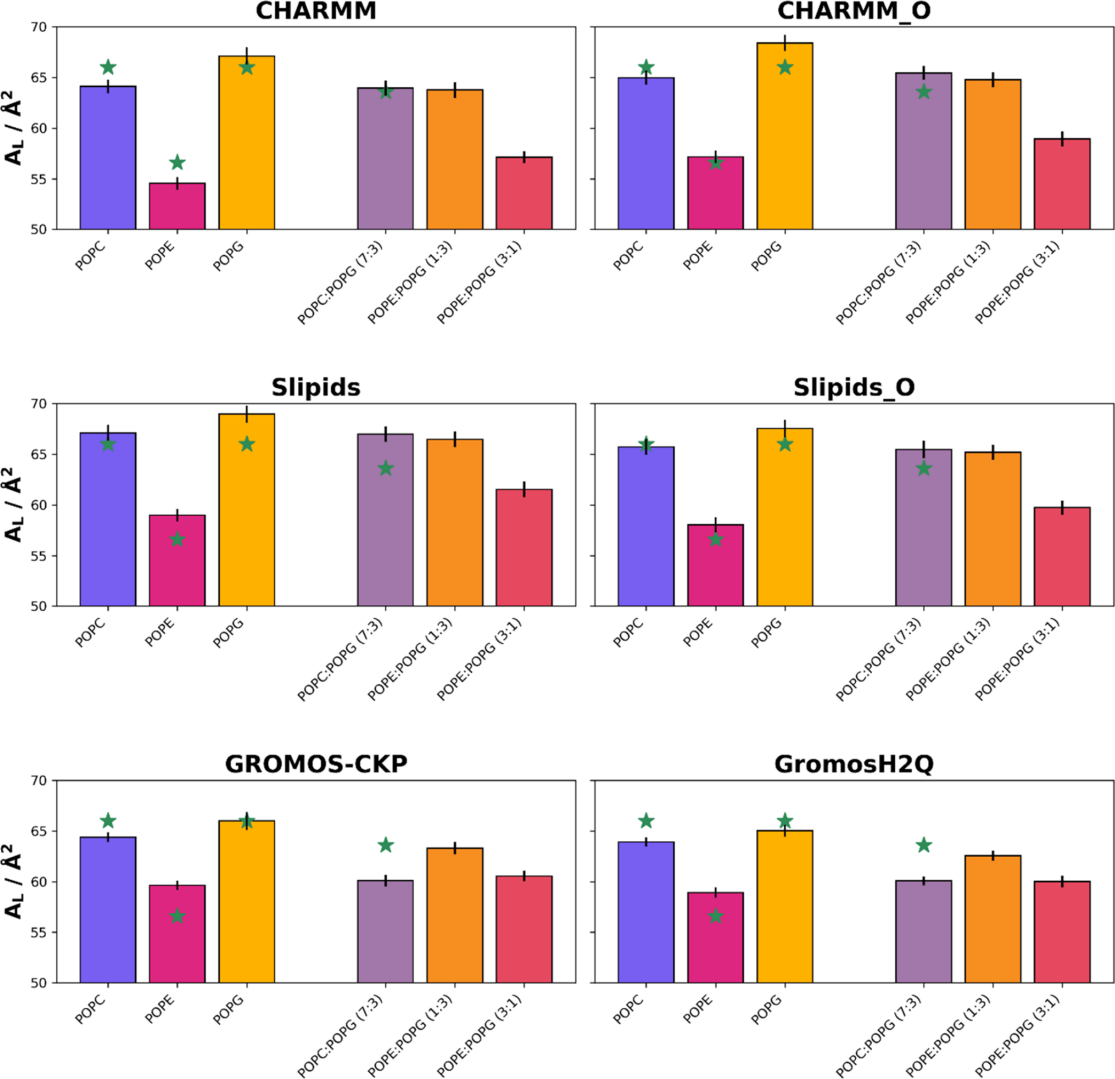
Area per lipid (*A*_L_, in Å^2^) obtained for the different force fields and simulation parameters employed in this work: CHARMM, CHARMM-O, Slipids, Slipids-O, GROMOS-CKP, and GROMOS-H2Q. The bars display the mean values obtained from the last 100 ns of the simulations, with error bars indicating the standard deviation. The green stars represent experimental values where available, which should be cautiously interpreted as they were measured at different temperatures and salt concentrations (refer to Table S1 for details). The bar colors represent lipid compositions: the colors for mixed membranes are combinations of the colors used for the pure membranes, blended in the same proportions as the lipid mixtures (7:3, 1:3, and 3:1 ratios), visually reflecting the composition of the membranes.

Simulations reveal that in general the *A*_L_ value of a lipid mixture closely aligns with the average of individual component *A*_L_ values, weighted by their proportions. This suggests that interactions within binary mixtures minimally affect the individual *A*_L_ values. One exception to this rule is the case of POPC/POPG (7:3), mainly for the two GROMOS parametrizations, leading to a minimum value of 60.07 ± 0.52 Å^2^ in GROMOS-CKP, while the values for both POPC and POPG are significantly higher (see above). A similar behavior is also observed for the rest of the force fields, although to a lesser extent (Table S1). This suggests synergistic interactions between the two different lipids, favoring the packing when they are mixed. This cannot be discarded due to the different structures of the two headgroups. However, this finding also reveals a clear difference between the GROMOS parametrizations and the other two force fields (CHARMM and Slipids). For the rest of the multicomponent simulations, differences across force fields seem to arise primarily from variations in pure components. The maximum *A*_L_ corresponds to the POPC/POPG (7:3) mixture in Slipids, leading to 66.99 ± 0.79 Å^2^, which is also lower than the *A*_L_ obtained for both POPC and POPG by using this force field and simulation parameters. POPE/POPG (1:3) presents the most closely clustered area values across the force fields for any of the mixtures, with the smallest difference observed between CHARMM and GROMOS-CKP at 63.76 ± 0.77 and 63.31 ± 0.62 Å^2^, respectively. Lastly, the POPE:POPG (3:1) system also exhibits a relatively narrow range of area values, from 57.14 ± 0.57 Å^2^ in CHARMM to 61.55 ± 0.77 Å^2^ in Slipids. The proximity of these values suggests that the force fields, although different, might capture the interactions and orientations of these lipids in a comparable manner. Other explanations are also possible, such as the possible screening of the interactions between POPG headgroups by ion binding. However, it is worth noting that there is a significant lack of experimental data for the lipid mixtures. The existence of such data would greatly aid in the validation and accuracy checks of simulation outputs, ensuring that the force fields and the results derived from them are aligned with real-world behavior.

When comparing the GROMOS-CKP and GROMOS-H2Q force fields, the results obtained are remarkably consistent across different lipid types and mixtures, making GROMOS-H2Q especially advantageous due to its ability to run simulations at a faster pace (by a factor of 3) by increasing the time step.

### 3.2 Bilayer Thickness

The thickness of each bilayer, calculated as the distance between the average positions of phosphorus (P) atoms in the two leaflets (P–P distance), is represented in Figures 3, S8 and Table S2. From the observed data, it can be concluded that the bilayer thickness values obtained from the MD simulations align reasonably well with experimental values, where available. This reinforces the capability of the selected force fields to capture the inherent structural properties of lipid bilayers. Different lipid compositions display varying thickness values, as expected. The mixed compositions tend to show deviations when compared to pure lipid bilayers. This is especially true for the POPC/POPG (7:3) mixture in the two GROMOS parametrizations, leading to a higher thickness than those of the corresponding pure components, in agreement with the lower *A*_L_—also compared to those of the monocomponent bilayers—observed for these two simulations (see above). Both GROMOS parametrization simulations produce results that are in close proximity to the known experimental values. The similarity in the results between GROMOS-CKP and GROMOS-H2Q is again exceptional. This implies that we can benefit from faster simulations using GROMOS-H2Q without compromising the accuracy of the results. The largest discrepancy with experimental values corresponds to the simulation of POPC with Slipids and, to a lesser extent, Slipids-O and to the simulations of pure POPE and the POPE/POPG (3:1) mixture with CHARMM. Unfortunately, no experimental thickness values are available for POPC/POPG (7:3) and for POPE/POPG (1:3). Significant differences exist between CHARMM and CHARMM-O for pure POPE, with the original parametrization of this force field yielding values closer to experimental results. For other systems, the variations between these two CHARMM variants are less pronounced. Differences between Slipids and Slipids-O remain minimal across all cases.

**Figure 3:**
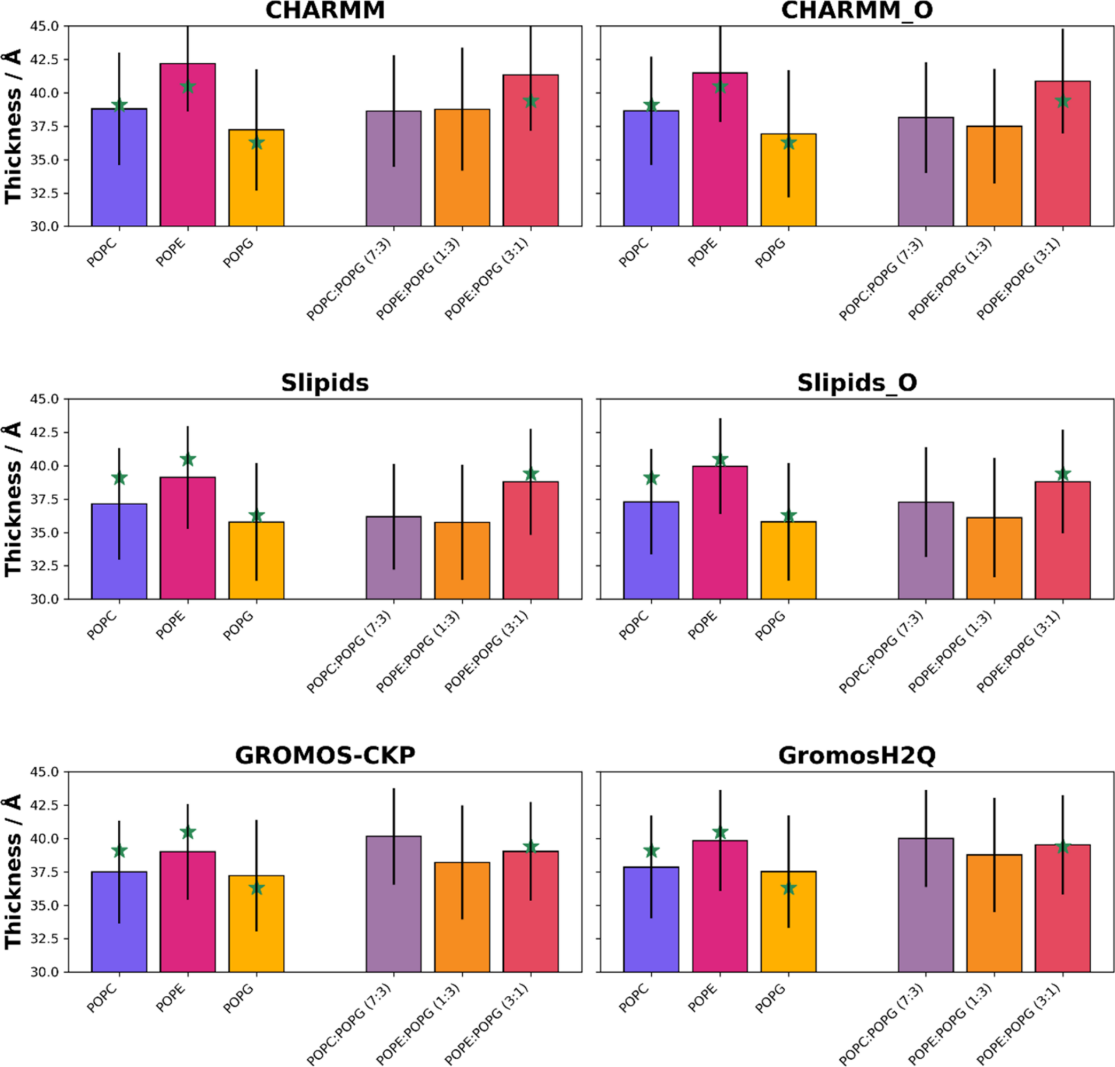
Bilayer thickness, calculated as a distance between average positions of phosphorus (P) atoms in the two leaflets of a bilayer (P–P distance), obtained for the different force fields and simulation parameters employed in this work: CHARMM, CHARMM-O, Slipids, Slipids-O, GROMOS-CKP, and GROMOS-H2Q. The bars display the mean values obtained from the last 100 ns of the simulations, with error bars indicating the standard deviation. The green stars represent experimental values where available, which should be cautiously interpreted, as they were measured at different temperatures and salt concentrations (refer to Table S2 for details). The bar colors represent lipid compositions: the colors for mixed membranes are combinations of the colors used for the pure membranes, blended in the same proportions as the lipid mixtures (7:3, 1:3, and 3:1 ratios), visually reflecting the composition of the membranes.

### 3.3 Lateral Density and Membrane Hydration

The density distributions along the bilayer normal, *z*, calculated for various components of all of the membrane compositions (Figure S9) are consistent with a well-equilibrated bilayer system. The distributions further indicate that water penetrates the bilayer up to the glycerol-ester region, while the methyl groups of the lipid tails are fully dehydrated, in agreement with the experiments.

To gain a deeper understanding of water penetration in membranes and its variation across force fields, we analyzed the hydration levels of various lipid compositions. This process involved quantifying the number of water molecules within the bilayer’s inner region, specifically between the glycerol lateral density peaks (Figure 4 and Table S3). Our findings exhibit a general trend where bilayers simulated with the CHARMM and Slipids force fields exhibit higher hydration levels compared with those using GROMOS-CKP and GROMOS-H2Q. This overarching observation is consistent across multiple lipid compositions and is critical for interpreting the structural and functional implications for membranes.

**Figure 4:**
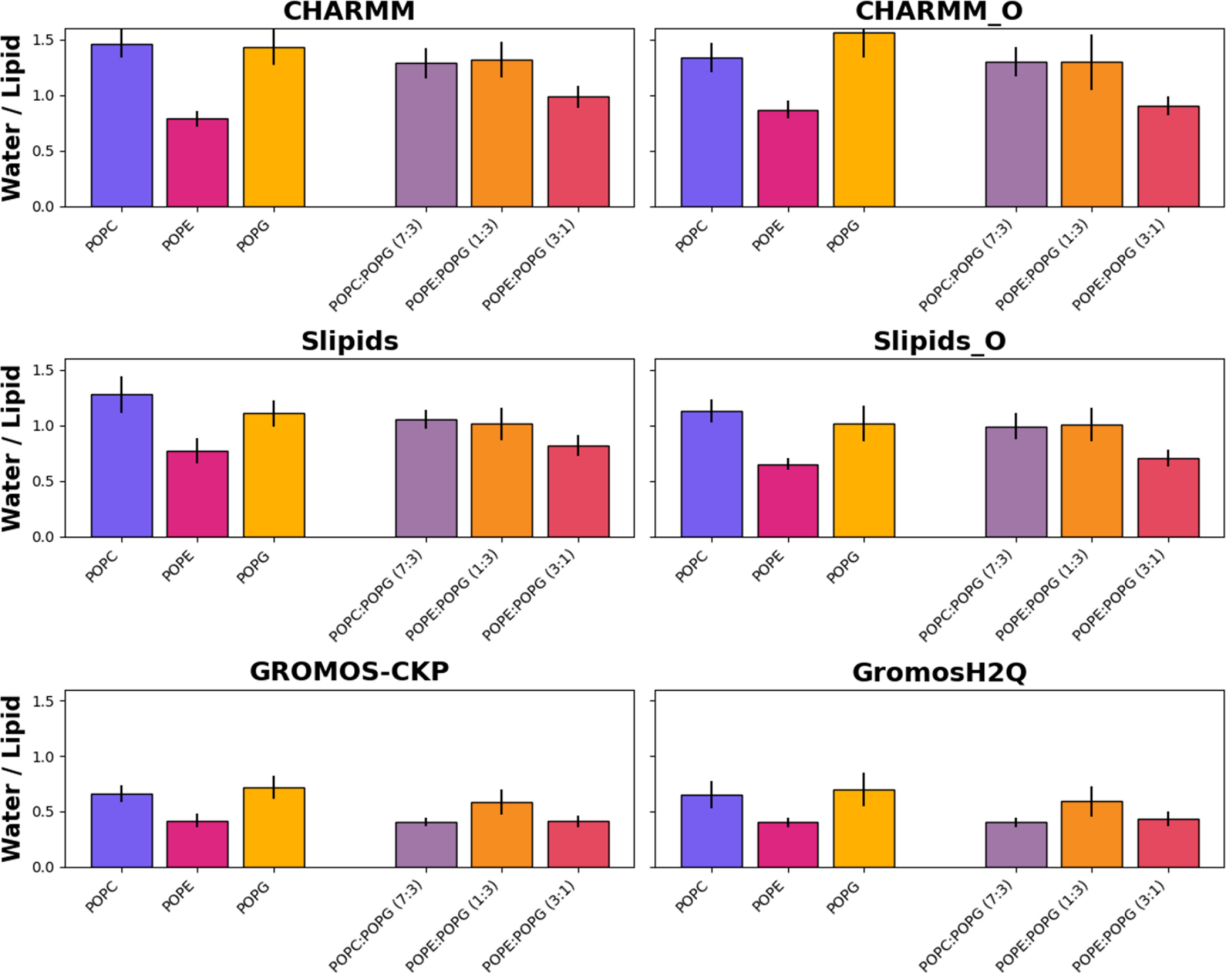
Number of water molecules per lipid molecule located within the bilayer’s inner region for the different force fields and simulation parameters employed in this work: CHARMM, CHARMM-O, Slipids, Slipids-O, GROMOS-CKP, and GROMOS-H2Q. The inner region is defined as the area situated between the glycerol lateral density peaks. The bars display the mean values obtained from the last 100 ns of the simulations, with error bars indicating the standard deviation (Table S3). The bar colors represent lipid compositions. The colors for mixed membranes are combinations of the colors used for the pure membranes, blended in the same proportions as the lipid mixtures (7:3, 1:3, and 3:1 ratios), visually reflecting the composition of the membranes.

Specifically, the CHARMM force field predicts a notably higher number of water molecules within the bilayer’s inner region. This happens using both sets of simulation parameters (CHARMM and CHARMM-O). For POPC, the CHARMM simulations suggest an average of 1.46 ± 0.13 water molecules per lipid, contrasted with 1.34 ± 0.13 using CHARMM-O and 1.28 ± 0.17 molecules predicted by Slipids and 1.13 ± 0.11 by Slipids-O. Conversely, both GROMOS-CKP and GROMOS-H2Q estimate substantially lower hydration levels for the same lipid at 0.660 ± 0.074 and 0.65 ± 0.12 molecules, respectively. This trend holds for other lipid types, as well. For POPE, the number of water molecules predicted by CHARMM and CHARMM-O is 0.786 ± 0.068 and 0.868 ± 0078, respectively, while GROMOS-H2Q shows a reduced hydration level at 0.404 ± 0.042. Similarly, for POPG, CHARMM and CHARMM-O estimate 1.43 ± 0.16 and 1.56 ± 0.23 molecules, with GROMOS-H2Q at the lower end with 0.70 ± 0.15. The trend extends to mixed lipid compositions like POPC/POPG (7:3) and POPE/POPG (1:3 and 3:1), where CHARMM and CHARMM-O, very similar to each other, consistently predict higher hydration than GROMOS-H2Q, with 1.29 ± 0.13 versus 0.402 ± 0.044 molecules for the former and 1.32 ± 0.16 versus 0.59 ± 0.14 molecules for the latter.

The different hydration levels are indicative of the sensitivity of membrane hydration to the choice of force field and simulation parameters, which can profoundly affect membrane properties such as fluidity, phase behavior, and permeability. The enhanced hydration predicted by CHARMM and Slipids could translate to a more fluid and potentially more permeable bilayer state, which might be more suitable under certain physiological conditions. In contrast, the lower hydration levels predicted by GROMOS-CKP and GROMOS-H2Q might suggest a tighter, less permeable bilayer, which could influence the membrane’s interaction with proteins, ions, and other heteromolecules. These variations underscore the importance of force field selection in molecular dynamics simulations, as the computational predictions need to align with experimental data to accurately reflect biological reality.

When examining the two GROMOS parametrizations, the similarity in their predictions for hydration levels across a variety of membrane compositions becomes apparent, suggesting a robust consistency in how these force fields handle the majority of lipid–water interactions. The close agreement between GROMOS-CKP and GROMOS-H2Q across different lipid types and their mixtures reinforces the confidence in these simulations in faithfully reproducing similar conditions. The results obtained for the POPC/POPG (7:3) are again anomalous in the sense that the number of waters in this bilayer is lower than those of the membranes with pure POPC or POPG. This happens for all of the force fields and simulation parameters, especially for the two GROMOS parametrizations, in agreement with the also anomalous values of A_L_ and thickness for this mixture.

### 3.4 2D Densities (in *XY*)

2D densities (in *XY*) were calculated for each leaflet using MDAnalysis in intervals of 10 ns, using bins with a width of 0.1 × 0.1 nm^2^ (see Methods). Upon examination of the provided heatmaps for various lipid membranes (Figures S10–S18), a few key observations can be discerned. Across all heatmaps calculated from the two-component membranes, there is evidence of lipid domain formation in comparison to the heatmaps of the single-component membranes. These domains, indicating areas of high lipid concentration, are present in varying sizes and distributions, suggesting differences in lipid interactions and organizational behaviors. Some heatmaps show pronounced clustering or lipid-type segregation, with distinct areas of high lipid concentration. This is particularly noticeable for the POPE:POPG mixtures, pointing to the importance of lipid–lipid interactions in shaping the overall membrane structure. In contrast, the heatmaps corresponding to the POPC/POPG (7:3) membrane display a more homogeneous lipid distribution, with less defined domain boundaries, probably due to the lower difference in the concentration ratio of these lipids in this mixture. However, the density of POPC in the mixture significantly increases with respect to that of the monocomponent membrane of this lipid (Figure S10). This happens with all force fields, especially with the two GROMOS parametrizations, and it could be related to the anomalous behavior of this system for the *A*_L_, thickness, and number of water molecules in the membrane region. It is worth noting that POPC and POPG have very similar melting temperatures in contrast to the much higher value of this property for POPE.^68,69^ These observations suggest a nonideal synergistic interaction between lipids in this particular mixture, specifically favoring the packing of POPC. Differences between simulation parameters (CHARMM vs CHARMM-O and Slipids vs Slipids-O) are not evident.

### 3.5 Radial Distribution Function

RDFs (Figure S19) are useful for understanding the spatial arrangement and interaction dynamics within lipid membranes. By analyzing the probability distribution of finding a lipid phosphate at a certain distance from a reference phosphate, we gain insights into the local ordering and structural properties of the bilayer. As for previously discussed properties, within the two GROMOS force field variants (GROMOS-CKP and GROMOS-H2Q), the RDF plots exhibit similar peak shapes and heights, underscoring a consistent representation of lipid–lipid interactions. The same happens when comparing CHARMM with CHARMM-O and Slipids with Slipids-O. Conversely, there is a clear distinction when comparing RDFs generated from GROMOS force fields to those from CHARMM and Slipids.

Both GROMOS-CKP and GROMOS-H2Q consistently provide higher peaks for the initial coordination shell in their RDFs, except for those of the PG-PG RDFs. This suggests a more compact arrangement or a heightened local structure compared to CHARMM or Slipids. A recurring observation is the earlier appearance of peaks in the GROMOS variants, implying a more condensed arrangement of the lipids in the bilayer. Such compactness can translate to various biophysical implications, such as a more densely packed membrane. Moreover, the proximate positioning of lipid headgroups could suggest reduced fluidity, potentially impacting membrane protein dynamics and overall membrane functions. This agrees with the significantly lower number of penetrating water molecules for the simulations with the two GROMOS parametrizations compared to those with CHARMM and Slipids with both sets of simulation parameters.

In particular, RDF plots for both POPC and POPE show that GROMOS variants exhibit more pronounced peaks with shorter PO4–PO4 distances compared with CHARMM and Slipids force fields. This points to denser packing in these models. Conversely, in the POPG membrane, GROMOS-CKP and GROMOS-H2Q display a significantly diminished initial peak compared to those of CHARMM and Slipids, which could be indicative of less localized density or different ion-binding characteristics affecting the local structure.

In mixed lipid compositions, the trends are more nuanced but follow a similar pattern. The GROMOS force fields often present a slightly more structured intermediate-range order, as evidenced by the sharper peaks beyond the first coordination shell than CHARMM and Slipids force fields. This could imply that the GROMOS family predicts more defined lipid domains or a different organization of lipid phases, which can be important for understanding the function of mixed membranes in biological systems. As for previous properties, in most scenarios, GROMOS-CKP and GROMOS-H2Q yield nearly identical outcomes. The denser packing of POPC molecules in the POPC/POPG (7:3) mixture is manifested by the sharper peak in the RDF corresponding to the PO4–PO4 distances of this lipid in these simulations for the two GROMOS parametrization. No significant changes are observed for the PC–PC RDF with CHARMM and Slipids with respect to the corresponding monocomponent membrane, regardless of the set of simulation parameters employed in both cases.

Wider peaks observed in certain force fields might signify a less compact distribution of lipid headgroups, affecting membrane fluidity and potential lipid domains. This is in good agreement with the observations made for 2D densities (Figures S10–S18), where the CHARMM and Slipids heatmaps are more diffuse than their GROMOS counterparts, which display some banding patterns. These disparities are pivotal for lipid raft interpretation as local environments and lipid packing can vary. These force field-specific variations might lead to unique biophysical properties in simulations, influencing lipid dynamics, membrane domain creation, protein interactions, and membrane responsiveness to external factors.

### 3.6 Number of H-Bonds

An integral aspect of lipid bilayer dynamics and stability is the network of hydrogen bonds established among lipid molecules. In this study, we investigated H-bonding patterns between different lipid groups, namely, headgroups and glycerols, and the solvent across the six lipid compositions. These interactions were monitored over the last 100 ns of the simulation trajectories and normalized as indicated in the methods section to facilitate comparative analysis across different lipid concentrations and force fields (Figures S20–S22 and Tables S4–S9). The values obtained for CHARMM and CHARMM-O are identical, within the uncertainties, and the same happens for Slipids and Slipids-O and for the two GROMOS parametrizations.

In the case of pure POPC bilayers, the GROMOS force fields show a slightly reduced H-bonding tendency between the headgroups compared to CHARMM and Slipids. In single-component POPE bilayers, the predominant interaction occurred between the headgroups of the POPE for CHARMM and Slipids, while for both GROMOS force fields, this switched to headgroup–glycerol interactions. This is the most significant difference between the impact of the parametrizations between force fields. On the other hand, the H-bond pattern for the pure POPG membrane was revealed to be relatively uniform across the four force fields, although a slightly lower proportion of H-bonds between the headgroup and the solvent molecules is observed for the two GROMOS parametrizations.

POPC/POPG (7:3) bilayers presented again a differentiated pattern of H-bonding across different force fields. The results of CHARMM indicate that the H-bonds between headgroups of POPG lipids represent the largest proportion of these interactions, followed by H-bonds between the headgroups and the solvent of POPC and, to a lower extent, of POPG. The number of H-bonds for Slipids and the GROMOS force fields is lower than for CHARMM, mainly those between the headgroups of POPG, although the difference from CHARMM to GROMOS is larger than that to Slipids.

For POPE/POPG mixtures, the trends depend on the ratio of the lipids. Differences between all force fields are significant; again, the pattern provided by the GROMOS force fields is almost identical to each other and more different from CHARMM and Slipids than the differences between these two last force fields. For the (1:3) ratio of POPE/POPG, the highest proportion of H-bonds corresponds to those between the headgroups of POPG with themselves and with the solvent, followed by far by the equivalent interactions for the headgroups of POPE. The same happens for Slipids, but in contrast to what happens with CHARMM, in this force field, the proportion of H-bonds between each headgroup and the solvent is higher than between headgroups of the same lipid. For the two GROMOS parametrizations, the results corresponding to the H-bonds of POPG headgroups are similar to what was observed for CHARMM, but in this case, the interactions between the headgroup of POPE and the glycerol of both POPG and POPE represent the highest proportion of these interactions. For the (3:1) ratio of the same lipids, the relative proportion of H-bonds involving POPE lipids becomes more important, but the trends are similar. Now, the differences between CHARMM and Slipids are lower than for the (1:3) ratio of these lipids and the interactions involving the headgroups of POPE with the headgroups of both lipids and with the solvent dominate the H-bonds interaction pattern, but the H-bonds between headgroups of different lipids are larger in Slipids than in CHARMM. For the two GROMOS force fields, the weight of the H-bonds between the headgroups of POPE and the glycerol of the same lipid provides the most significant contribution to this type of interaction, screening mainly the number of H-bonds between headgroups of POPE with themselves.

In general, the most significant difference between all force fields is that the interaction between headgroups and glycerol of POPE molecules are much more important for both GROMOS parametrizations than for CHARMM and Slipids, and this makes a significant impact in the mixtures involving this lipid. The differences with POPC and POPG between force fields exist, but they are marginal compared to those with POPE.

### 3.7 *S*_CH_ Order Parameters

The fidelity of MD simulations in replicating the physical reality of lipid bilayers has long been gauged against experimental data, with NMR spectroscopy being the gold standard. Among the various parameters accessible through NMR, the C–H bond order parameters (*S*_CH_) are particularly valuable as they provide a direct measure of the lipid chains’ conformational order combined with the overall motion of the molecule. It is these parameters that simulations strive to accurately predict, as their congruence with experimental values is a strong indicator of the simulations’ accuracy. By incorporating the calculated distributions of C–H tilt angles (θ) from atomistic simulations into the corresponding equation, we can derive order parameters that should, in theory, correspond to the values determined by NMR. A match between the simulated and experimental order parameters lends credence to the conformational ensemble and the overall motional geometry of the molecules produced by the simulation, suggesting that it is a realistic representation at an atomistic resolution. Obviously, the replication of order parameters alone is not a definitive proof of structural accuracy since multiple, distinct structural models could yield identical values for this property.^70^ Therefore, while the agreement between the simulated and experimental order parameters is a necessary condition for validation, it is not sufficient. Additional structural data and constraints are often required to ensure the robustness of the model.

The complexity of lipid mixtures, especially in bacterial membrane models, is an area that has been relatively unexplored in biophysical research. While studies on the order parameters of single-component lipid systems are more common, the intricate interactions and diverse compositions of bacterial membranes present a significant challenge, leading to a dearth of experimental data for these systems. In fact, order parameters for lipid mixtures that mimic the multicomponent nature of bacterial membranes have not been extensively documented or published. This uncharted territory is explored here. By utilizing NMR spectroscopy, we are not only filling a critical void in the experimental literature but also providing essential data that will enhance the accuracy and relevance of MD simulations in representing the bacterial membrane structure.

In the *sn-2* chains (Figure 5, right column), the order parameters decrease as we move toward the terminal end of the chains, with a prominent kink at the middle carbon atoms due to the double bond in the tail. This feature is consistent across different force fields and lipid types, although force-field-dependent quantitative differences are clear. The order parameters of the *sn-1* chains (Figure 5, central column) continuously decrease from the glycerol linkage toward the end of the tail. This behavior is also uniform across the different force fields and simulation parameters.

**Figure 5:**
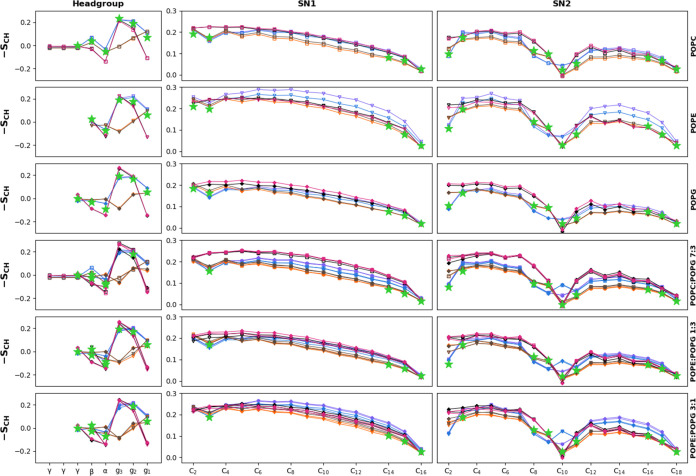
Order Parameters for the headgroups and aliphatic chains for the different studied membranes. Headgroups appear in the small subplots on the left. Marker types differentiate specific lipids (square for POPC, triangle for POPE, and plus sign for POPG). The color indicates the force field (violet for CHARMM, navy blue for CHARMM-O, orange for Slipids, brown for Slipids-O, magenta for GROMOS-CKP, and black for GROMOS-H2Q). The experimental values are plotted over the simulation results as green stars. It is important to note that experimentally, it is not possible to distinguish between these two types of chains or to identify all of the carbon atoms in the tails, except for the carbons participating in double bonds and the adjacent ones.

POPE consistently exhibits higher order parameters for the acyl chains compared to the other lipids, a trend that is distinctly visible in pure membranes as well as in bacterial membrane models with a higher content of POPE. This agrees with the experimental order parameters and potentially explains the higher melting temperatures measured for PEs comparatively to PCs and PGs.^68,69^ This observation holds true across all utilized force fields, suggesting a fundamental characteristic of POPE in promoting a more ordered membrane state and a tendency toward more rigid and tightly packed bilayers and therefore an increase in membrane thickness. No significant differences are appreciated between CHARMM and CHARMM-O or between Slipids and Slipids-O. Such ordered membranes might exhibit decreased permeability and potentially altered dynamics of membrane proteins, impacting biological functions, such as signaling and transport. In mixtures, the acyl chain order parameters of the distinct lipids are equivalent, i.e., there is a homogeneous behavior for the acyl chains in the mixtures. This suggests that the selectivity in the interaction between proteins and lipids or membranes of different compositions is mainly due to the headgroups.

Further analysis reveals that the GROMOS family of force fields (magenta and black lines, for CKP and H2Q flavors, respectively) provides remarkably similar values for both the *sn-1* and *sn-2* tails across different lipid types, with their curves nearly superimposable, indicating a conserved prediction of chain order for this part of the lipid molecules within these force fields.

Regarding the performance of individual force fields, Slipids consistently yield the lowest order parameters for all membranes examined, both with the common simulation parameters and with the original ones, which could imply a prediction of more fluid and dynamic membrane states. This is in accordance with previous studies and comparatively to CHARMM, which presents more ordered chains as a result of reduced electrostatic 1–4 interactions for Slipids due to the absence of partial charges for acyl chain methylenes,^71^ an effect that is observed to a lesser extent when using the original simulation parameters. This characteristic could potentially affect membrane protein function, diffusion processes, and the overall mechanical properties of the bilayer.

The GROMOS family generally predicts higher order parameters, indicative of a more ordered and less dynamic lipid environment, which could have implications for the barrier properties and stability of membranes. However, in cases like the pure POPE and POPE/POPG (3:1) membranes, the CHARMM force field predicts the highest order parameters, surpassing the GROMOS force fields, even when using the original set of simulation parameters. This might reflect CHARMM’s unique parametrization that captures a more ordered state in POPE-rich systems, potentially influencing the mechanical and physicochemical properties of the membranes.

In addition to our simulations, experimental NMR data (as described in Section 2.2 and Figures S22–S30) provide a reference for validating our computational results (Figure 5, green stars). Confirming previous studies,^45,71^ the Slipids and Slipids-O force fields exhibit the strongest correlation with experimental NMR values for the aliphatic chains, particularly mirroring the *sn-2* tail order parameters. This force field also performs very well in capturing the order parameters of the three multicomponent membranes. For the headgroups, CHARMM and CHARMM-O most accurately replicate the NMR-derived experimental data for glycerols, where no significant deviations are observed between the two sets of simulation parameters, while for the polar heads, no single force field emerges as the definitive best fit. Instead, the experimental results tend to align with a balanced average of all of the parametrizations under consideration.

In order to assess how closely each parametrization followed the experimental results for the order parameters, the mean squared error between simulation results and the experiment was calculated (Figure 6), separating the contributions of headgroups, *sn-1* and *sn-2* tails. For all force fields, the results that resembled the experimental measurements the most were those of the *sn-2* tails, this is, the ones containing the unsaturation. There are some exceptions in this trend, as the GROMOS force fields capture the headgroup OPs (order parameters) slightly better than those of the *sn-2* tails for the POPC/POPG (7:3) membrane, and the *sn-1* tail (fully saturated) is the best captured too in POPE/POPG (3:1) mixtures; an effect shared by Slipids-O. For Slipids, and the GROMOS parametrizations too (although to a lesser extent), the agreement between the results for the headgroups and experimental data is worse than for both CHARMM parametrizations and also worse than for the tails in all force fields. For CHARMM and CHARMM-O, however, there is comparable agreement for the aliphatic chains and headgroups for all of the studied systems. This is not surprising since CHARMM36 was optimized to reproduce such values.^72^ In summary, no significant differences are observed between the order parameters of the original and the modified CHARMM and Slipid parametrizations, and CHARMM produces the best order parameters compared to the experimental values for both the headgroups and the tails, followed by GROMOS and Slipids. The results for the headgroups using Slipids are significantly further from experimental values than those provided by GROMOS and CHARMM.

**Figure 6:**
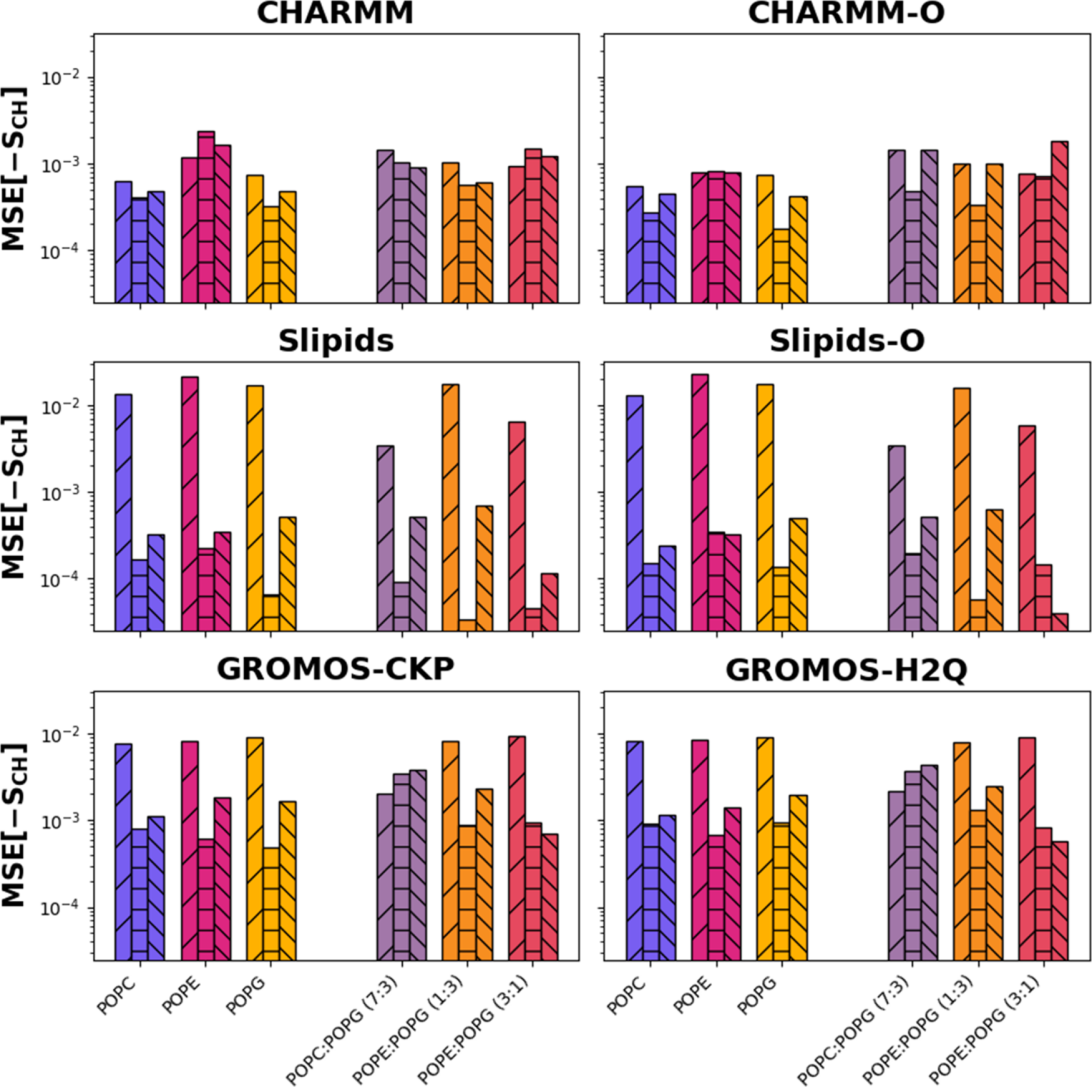
Mean squared errors for the order parameters, in logarithmic scale, of the headgroups (forward slash hatching), *sn-2* (horizontal hatching), and *sn-1* (backward slash hatching) tails, calculated against the experimental values, when available. The bar colors represent lipid compositions. The colors for mixed membranes are combinations of the colors used for the pure membranes, blended in the same proportions as the lipid mixtures (7:3, 1:3, and 3:1 ratios), visually reflecting the composition of the membranes.

### 3.8 Lipid Lateral Displacement

The lateral displacement of lipid molecules is a fundamental aspect that profoundly affects the physical state and biological functionality of cellular membranes. This movement is indicative of the fluid nature of the lipid bilayer, allowing for essential processes such as membrane fusion, protein function, and lipid signaling. Understanding the extent and pattern of lipid lateral displacement can provide insights into the viscosity and heterogeneity of the membrane, as well as the potential for domain formation within the lipid bilayer.^73^ In computational studies, this displacement is often quantified through MD simulations, where the trajectories of individual lipid molecules are tracked over different windows of time (see Section 2.2) to generate a statistical view of their motion. By comparing these movements across different force fields and simulation parameters and across all types of lipids and membrane compositions, we observe that the displacement profiles generally show a single peak, indicating that the majority of lipids undergo a similar extent of lateral movement within the time window (Figures 7 and S31–S33). The appearance of two peaks would indicate the coexistence of two different diffusion regimes for the lipids in the bilayer. This would be possible, for instance, in the presence of a macromolecule embedded in the membrane, slowing down the movement of the neighboring lipid units.

**Figure 7:**
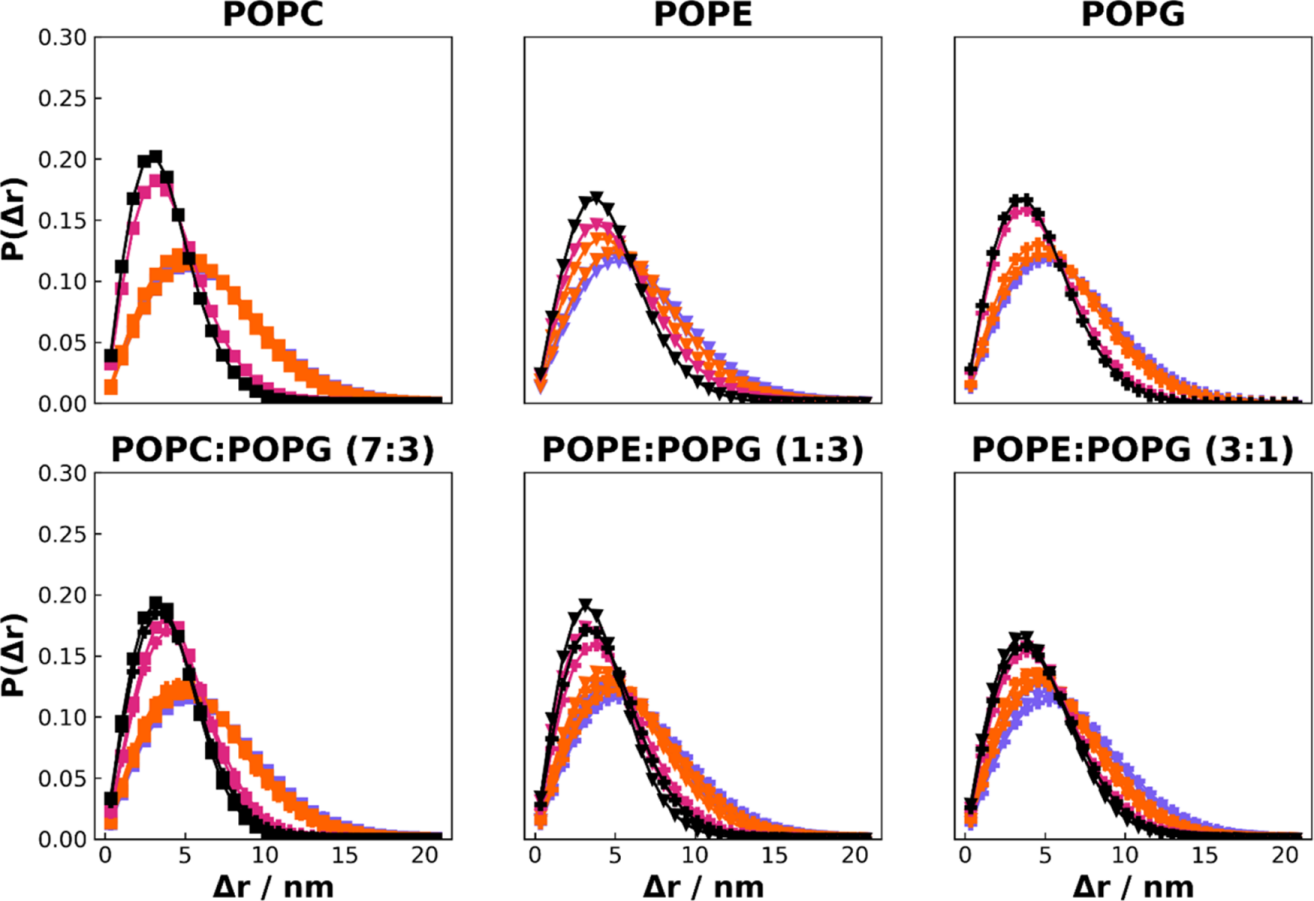
Probability density for the lateral displacement for each lipid type in a window time of 5 ns, calculated along the last 100 ns of the trajectory. Marker types differentiate specific lipids (square for POPC, triangle for POPE, and plus for POPG), and color indicates force field (violet for CHARMM & CHARMM-O, orange for Slipids & Slipids-O, magenta for GROMOS-CKP, and black for GROMOS-H2Q). The results obtained using window times of *t* = 2 and *t* = 10 ns are represented in Figures S31 and S33.

The lateral displacement profiles of lipids in various membrane compositions provide intriguing insights into the dynamics governed by different force fields. In the simulations, the behavior of lipids modeled with the GROMOS family of force fields, namely, GROMOS-CKP and GROMOS-H2Q, demonstrates remarkable consistency within its group yet distinctly varies from the trends observed in Slipids and CHARMM. The use of the different simulation parameters in CHARMM and Slipids provides just slightly different behaviors. These force fields exhibit broader distribution curves in the lateral displacement of lipids. Such wider distributions suggest a more fluid-like character within the membrane environment as described by CHARMM and Slipids, implying higher diffusion rates for lipids and potentially for membrane proteins.

Interestingly, minor differences between the GROMOS-CKP and GROMOS-H2Q force fields become more pronounced at longer time windows. However, these discrepancies are not substantial, especially when considering the effect of hydrogen mass, which is quadrupled in GROMOS-H2Q simulations due to the explicit representation of hydrogen atoms. This observation hints at the robustness of the GROMOS-H2Q parametrization, where even the increased mass of explicit hydrogens does not dramatically alter the lipid displacement behavior. The difference between CHARMM and CHARMM-O, as well as between Slipids and Slipids-O, decreases at longer time windows.

Furthermore, it is notable that in mixed lipid membranes, the lateral displacement appears to be independent of the type of lipid. Different lipids exhibit nearly identical displacement behaviors across all examined force fields. This finding underscores a level of consistency in the physical movement of diverse lipid species within the bilayer, suggesting that despite the chemical diversity, the force fields predict a similar mobility pattern within the complex membrane environment.

The lateral displacement of lipids serves as a precursor to understanding the diffusion characteristics within the membrane, with the observed distributions providing the basis for calculating diffusion coefficients (Figures S34–S36 and Tables S10–S12). These coefficients reaffirm the trends we noted in the dynamic behaviors of the different force fields. The broader and lower displacement profiles for CHARMM and Slipids correlate with higher diffusion coefficients, indicating more rapid lipid mobility. Conversely, the narrower displacement distributions for the GROMOS family correlate with lower diffusion coefficients, suggesting a more restrained lipid movement.

The lateral diffusion coefficients obtained from our simulations align with certain values reported for the same lipids in the existing literature.^74,75^ This concordance provides a measure of validation for the computational approaches and parameters used. However, it is worth acknowledging the complexities involved in comparing these simulation-derived coefficients with those from experimental studies. One of the primary complicating factors is the dependence of the simulated diffusion coefficients on the size of the simulation box. This issue arises due to the use of periodic boundary conditions (PBCs), which can artificially impact lipid mobility by influencing the time scale of diffusive behavior that can be observed.^76–78^ Furthermore, the level of hydration in the system plays a crucial role;^79^ a more hydrated bilayer may facilitate more significant lipid movement, leading to higher diffusion rates. In fact, CHARMM and Slipids predict higher hydration values than both GROMOS force fields, in line with the higher diffusion coefficients observed. Similarly, the time window over which the lateral displacement is calculated can dramatically affect the resultant diffusion coefficient, with longer windows potentially capturing more complex and slower-moving dynamics. Even within computational simulations, varying these parameters can yield divergent results, highlighting the challenge of achieving direct comparisons with experimental data. The discrepancies observed underscore the need for meticulous methodological consistency and comprehensive reporting in both simulation and experimental protocols to enable more reliable and meaningful comparisons of the lipid dynamics across these two domains. To facilitate direct comparison between force fields, all calculations were performed using identical methods and metaparameters in addition to the original simulation parameters of CHARMM and Slipids. This ensures a reliable comparison of force field performance, allowing us to say that the diffusion using CHARMM or Slipids for all of the tested membrane models and for the three different time windows is significantly higher than using any of the GROMOS parametrizations. The differences in the estimated diffusion coefficients using different metaparameters are significantly lower than those between the force fields. This agrees with the conclusions from the previous analysis, indicating that the membranes simulated using GROMOS are tighter and thicker and with fewer water molecules penetrating the bilayer region. In contrast with the quantitative parameters obtained from other analyses, no special behavior is observed for the POPC/POPG (7:3) membrane composition.

### 3.9 Area Compressibility Moduli

The area compressibility modulus is a fundamental mechanical property of lipid bilayers that quantifies the resistance or response of the membranes to changes in the surface area. It is an essential parameter for understanding the biophysical behavior of cell membranes, influencing various cellular processes, such as membrane fusion, signaling, and the function of membrane proteins. A higher *K*_A_ indicates a stiffer membrane, which resists deformation, while a lower *K*_A_ suggests a more flexible membrane. The lipid concentration in the membrane can significantly affect the *K*_A_, and this property is expected to be sensitive to the force field parametrization. Our results (Figure S37) indicate that CHARMM and Slipid parametrizations exhibit quite uniform *K*_A_ values, with subtle differences between bilayer compositions. This also suggests minor impacts of parameter modifications on membrane compressibility. GROMOS-CKP shows much greater variability in *K*_A_ values, with POPE having the highest compressibility (∼500 mN·m^–1^) and POPG the lowest (∼140 mN·m^–1^). GROMOS-H2Q force field stands out with significantly higher *K*_A_ values overall, ranging from ∼540 to 930 mN·m^–1^. In this force field, POPC and POPC/POPG (7:3) show the highest compressibilities. Mixed lipid systems (POPC/POPG ratios) generally show intermediate *K*_A_ values between their pure counterparts, with some variations depending on the force field. An exceptional case is again GROMOS-H2Q with these mixtures exhibiting higher compressibilities than their pure components. The *K*_A_ values provided by the two GROMOS parametrizations are unrealistically high when compared to typical values obtained from different computational and experimental methods.^60^

## 4 Conclusions

In this study, we employed molecular dynamics (MD) simulations to analyze bacterial membrane models, focusing on a range of lipid compositions. The simulations were conducted using various force fields, namely, CHARMM, Slipids, and two GROMOS variants, as well as two different simulation parameters for CHARMM and Slipids. The aim is to assess their effectiveness and differences in replicating the properties of these biological systems. Experimental order parameters using nuclear magnetic resonance (NMR) spectroscopy provide a critical benchmark for evaluating the accuracy of the simulations.

The area per lipid (*A*_L_) analysis showed that Slipids generally presented the highest *A*_L_ values, suggesting looser lipid packing, which became particularly evident in simulations of POPC lipids. This higher *A*_L_ in Slipids aligned well with experimental values, indicating its effectiveness in mimicking realistic membrane structures. On the other hand, GROMOS force fields, particularly GROMOS-H2Q, displayed lower *A*_L_ values, indicative of a tighter lipid arrangement. The use of different simulation parameters makes significant differences in some cases, especially between CHARMM and CHARMM-O for POPE, which provides a particularly low *A*_L_ value for CHARMM.

Different conclusions can be obtained from the quantification of the bilayer thickness. CHARMM and Slipids closely matched experimental data, demonstrating their accuracy in capturing the structural properties. Again, there is a significant difference between CHARMM and CHARMM-O for POPE, with the rest of the systems giving very similar values for both sets of simulation parameters. The GROMOS variants, and notably GROMOS-H2Q, also showed remarkable alignment with experimental values, coupled with the added benefit of increased efficiency in simulation time due to a larger time step. The analysis of solvent penetration led to surprisingly different results for different force fields. CHARMM and Slipids integrate many more water molecules in the lipid bilayers, implying a more fluid and permeable structure, while GROMOS variants showed much lower hydration levels. The simulation parameters employed in CHARMM and Slipids do not have a large impact on this property. These distinctions are crucial for understanding the implications of membrane fluidity, phase behavior, and permeability. All of these properties are highly sensitive to the membrane composition. It is worth highlighting the behavior of the system with POPC/POPG (7:3), which seems to demonstrate a significant synergy between the interaction of these two lipids, as revealed by the lower *A*_L_, higher thickness, and lower penetrating water molecules compared to the monocomponent membranes with POPC or POPG. This behavior is reproducible with all of the force fields but more marked with both GROMOS parametrizations.

H-bonding patterns across the force fields varied significantly but not between simulation parameters. CHARMM and Slipids showed a tendency for higher normalized H-bonding, particularly involving glycerol groups and solvent interactions, suggesting a more dynamic membrane structure. GROMOS force fields, in contrast, favored glycerol interactions with POPE headgroups, pointing toward a different membrane structural organization. The radial distribution function (RDF) analysis further illuminated the differences in the lipid arrangements. GROMOS variants, especially GROMOS-H2Q, revealed a more compact and structured lipid arrangement, implying a reduced fluidity and denser packing of the membrane. Such an arrangement has significant implications for biophysical properties like protein dynamics and overall membrane functions, as reflected in the extremely high area compressibility moduli of all membranes using this force field parametrization.

The use of NMR spectroscopy to determine the order parameters for various lipid compositions provided a robust benchmark for simulation accuracy. The experimental data were instrumental in validating the simulation results. CHARMM better reproduces the order parameters of the headgroups, while Slipids provide better values for the acyl chains. In contrast, the order parameters of the headgroups using Slipids are significantly further from the experimental values than those provided by GROMOS, even more, by CHARMM. This happens for both monocomponent and multicomponent bilayers. The two GROMOS parametrizations provide very similar results to each other and represent an intermediate case between Slipids and CHARMM, with no exceptional results for any lipid group but with less differences with respect to the experimental values than the least reliable force field for each group.

This study highlights the importance of carefully selecting the appropriate force field for specific membrane properties in MD simulations. Each force field displayed unique strengths in modeling different aspects of the bacterial membranes. The consistency between GROMOS-CKP and GROMOS-H2Q across various properties, coupled with the high efficiency of the latter, makes it particularly interesting for this type of simulation. Different simulation parameters in CHARMM and Slipids generally do not impact most of the analyzed properties. Significant differences were observed in the area per lipid and thickness of pure POPE, while other properties and systems show similar results across both parameter sets. The force field’s sensitivity to simulation parameters, such as Lennard-Jones interactions and algorithm-specific cutoffs, is noteworthy since small variations in these parameters can lead to significantly faster calculations and longer trajectories. Additionally, low sensitivity in simulation parameters suggests the robustness of a force field under various conditions beyond its initial parametrization.

The comparison of a set of different structural and dynamic properties for a variety of lipid compositions is interesting, showing nearly ideal behavior for the two studied mixtures of POPE and POPG, contrasting with the highly synergistic interaction between POPC and POPG in the membrane formed by these two lipids. The fact that this behavior is reproducible at different extents, at least for some properties, for the six parametrizations makes it trustworthy. Thus, the new insights gained from this research contribute significantly to our understanding of membrane biophysics and pave the way for more accurate and comprehensive models of bacterial membranes.

## Supplementary Material



## Notes

Detailed 2D and 3D lipid models from simulations; time evolution analyses of lipid bilayers; lateral density distributions; 2D densities for various lipids; comparative radial distribution functions; spectral analysis including NMR peak assignments and order parameters; probability density plots; tables with quantitative data like area per lipid; bilayer thickness; and diffusion coefficients (PDF)
