# Supplementary material for: Simulating Bacterial Membrane Models at the Atomistic Level: A Force Field Comparison
Source: J Chem Theory Comput. 2024 Sep 3;20(18):8290–307. doi: 10.1021/acs.jctc.4c00204 (PMC12442013; doi:10.1021/acs.jctc.4c00204)
Supplement: Supplementary file 1 [file ct4c00204_si_001.pdf]

Supporting Information

for

Simulating bacterial membrane models at atomistic-level: A force field comparison

Alexandre Blanco-González[1,2,3], *Anika Wurl*[4], *Tiago Mendes Ferreira*[4], *Ángel Piñeiro*[1*], *Rebeca Garcia-Fandino*[2*]

[1]Facultad de Física, University of Santiago de Compostela (USC), Santiago de Compostela, Spain.
[2]Singular Research Centre in Chemical Biology and Molecular Materials, (CIQUS), Organic Chemistry Department, University of Santiago de Compostela (USC), Santiago de Compostela, Spain.
[3]MD.USE Innovations S.L, Edificio Emprendia, 15782 Santiago de Compostela, Spain.
[4]Institute of Physics, Faculty of Natural Sciences II, Betty-Heimann-Str. 7, 06120 Halle/Saale, Germany.

*Corresponding author. E-mail addresses: angel.pineiro@usc.es and rebeca.garcia.fandino@usc.es

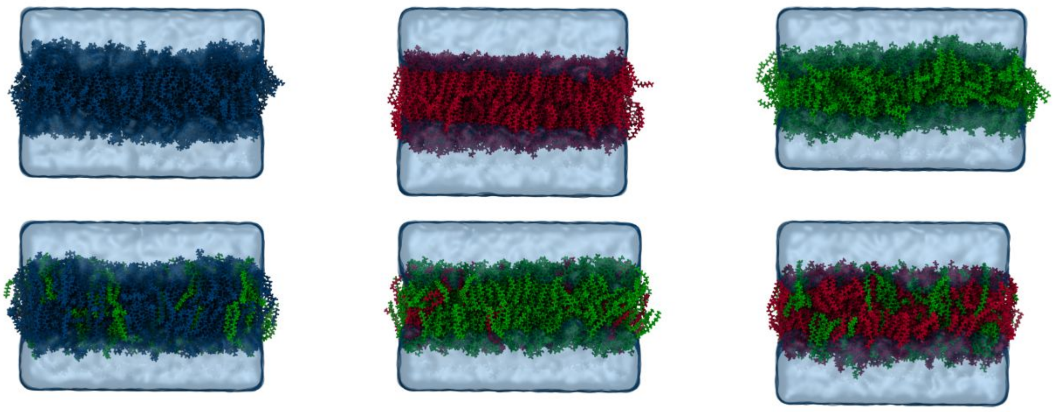

Figure S1. 3D snapshots of six membrane compositions at t=500 ns, using CHARMM (common parameters): Pure POPC (blue), POPE (red) and POPG (green) membranes, and bacteria model membranes: 7:3 POPC:POPG (bacterial inner membrane model), 1:3 POPG:POPE (Gram-negative bacteria model), and 3:1 POPG:POPE (Gram-positive bacteria model).

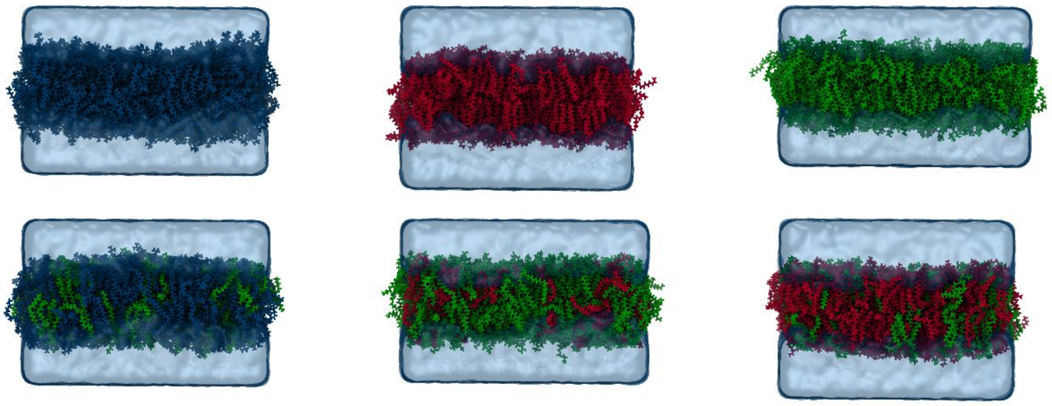

Figure S2. 3D snapshots of six membrane compositions at t=500 ns, using CHARMM-O: Pure POPC (blue), POPE (red) and POPG (green) membranes, and bacteria model membranes: 7:3 POPC:POPG (bacterial inner membrane model), 1:3 POPG:POPE (Gram-negative bacteria model), and 3:1 POPG:POPE (Gram-positive bacteria model).

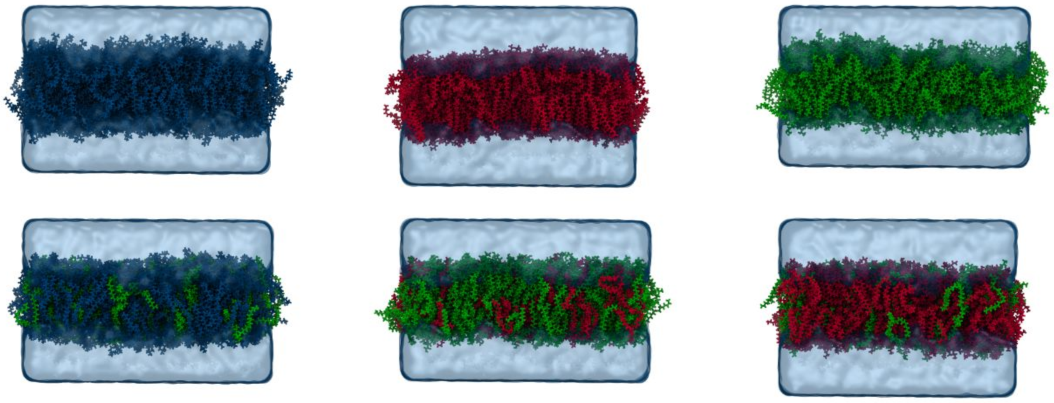

Figure S3. 3D snapshots of six membrane compositions at t=500 ns, using Slipids (common parameters): Pure POPC (blue), POPE (red) and POPG (green) membranes, and bacteria model membranes: 7:3 POPC:POPG (bacterial inner membrane model), 1:3 POPG:POPE (Gram-negative bacteria model), and 3:1 POPG:POPE (Gram-positive bacteria model).

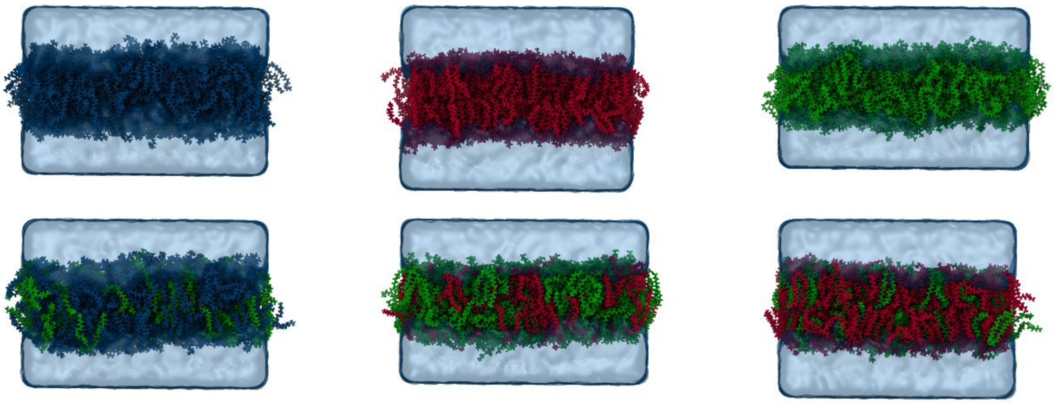

Figure S4. 3D snapshots of six membrane compositions at t=500 ns, using Slipids-O: Pure POPC (blue), POPE (red) and POPG (green) membranes, and bacteria model membranes: 7:3 POPC:POPG (bacterial inner membrane model), 1:3 POPG:POPE (Gram-negative bacteria model), and 3:1 POPG:POPE (Gram-positive bacteria model).

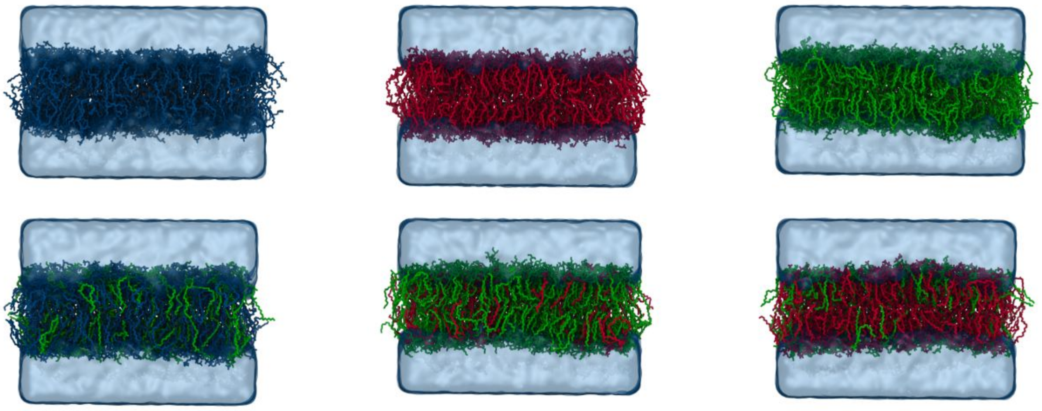

Figure S5. 3D snapshots of six membrane compositions at t=500 ns, using GROMOS-CKP: Pure POPC (blue), POPE (red) and POPG (green) membranes, and bacteria model membranes: 7:3 POPC:POPG (bacterial inner membrane model), 1:3 POPG:POPE (Gram-negative bacteria model), and 3:1 POPG:POPE (Gram-positive bacteria model).

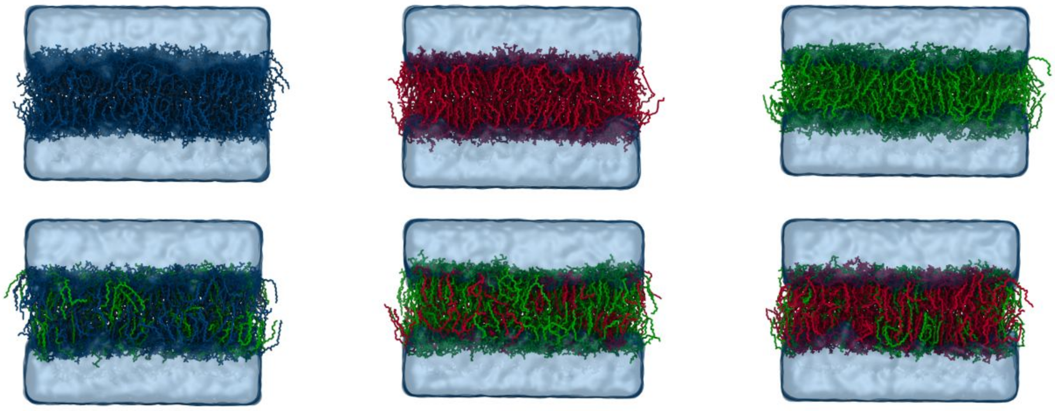

Figure S6. 3D snapshots of six membrane compositions at t=500 ns, using GROMOS-H2Q: Pure POPC (blue), POPE (red) and POPG (green) membranes, and bacteria model membranes: 7:3 POPC:POPG (bacterial inner membrane model), 1:3 POPG:POPE (Gram-negative bacteria model), and 3:1 POPG:POPE (Gram-positive bacteria model).

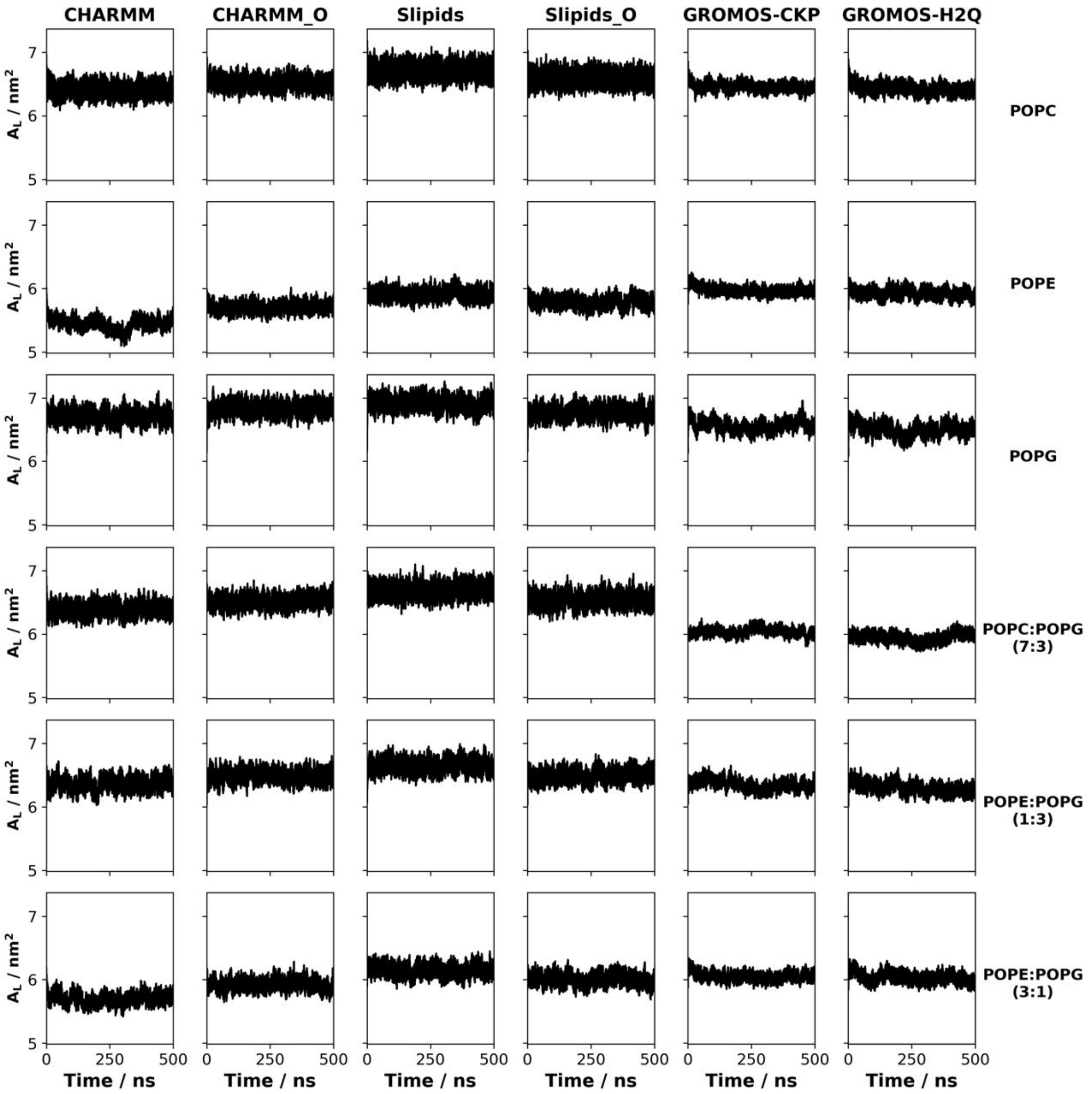

Figure S7. Time evolution of the area per lipid (A_L) along the simulated time (500 ns) for all the forcefields and all the lipid compositions studied.

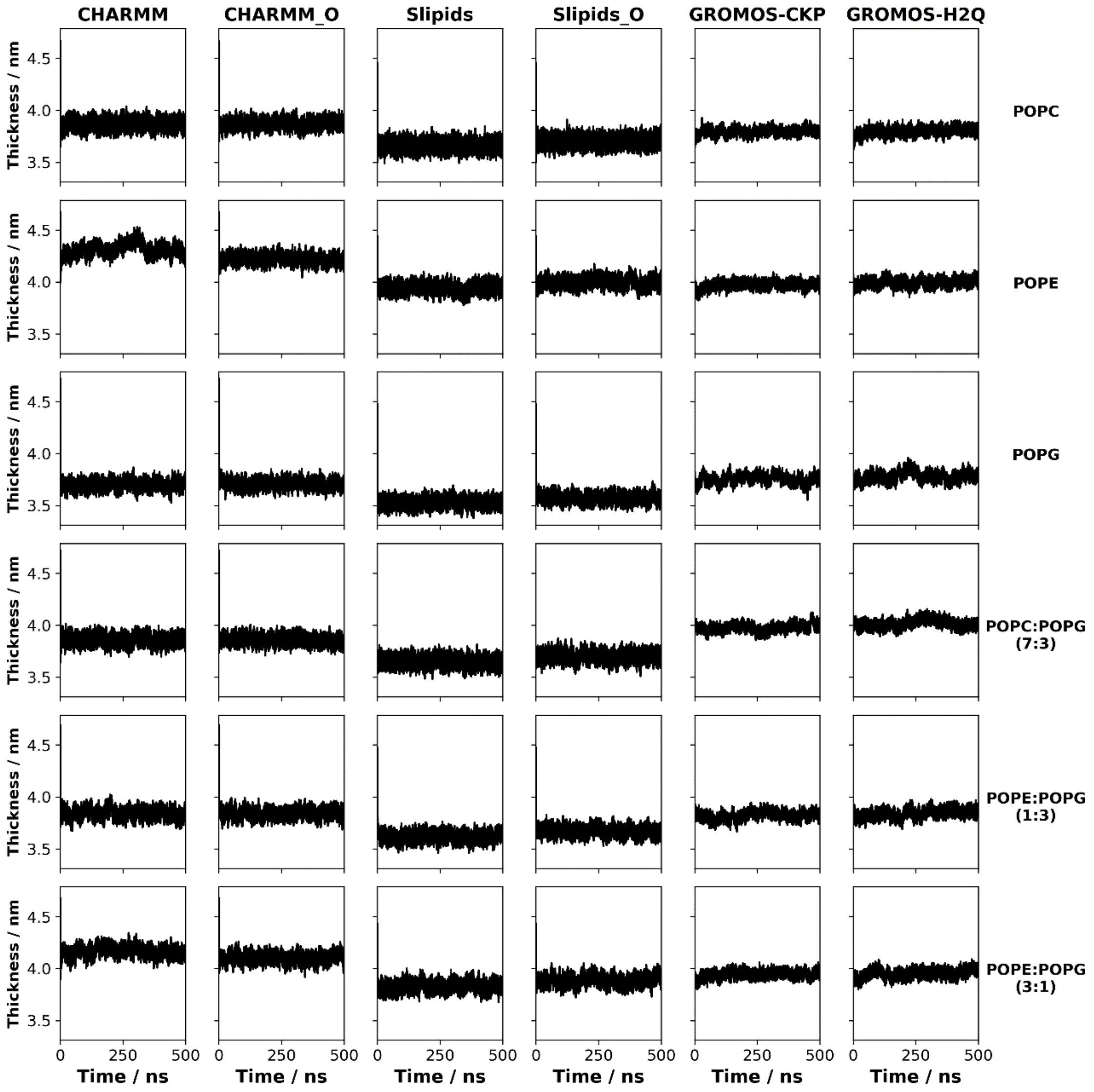

Figure S8. Time evolution of the thickness of each bilayer, along the simulated time (500 ns), calculated as a distance between average positions of phosphorus (P) atoms in the two leaflets of a bilayer (P-P distance), for all the forcefields and all the lipid compositions studied.

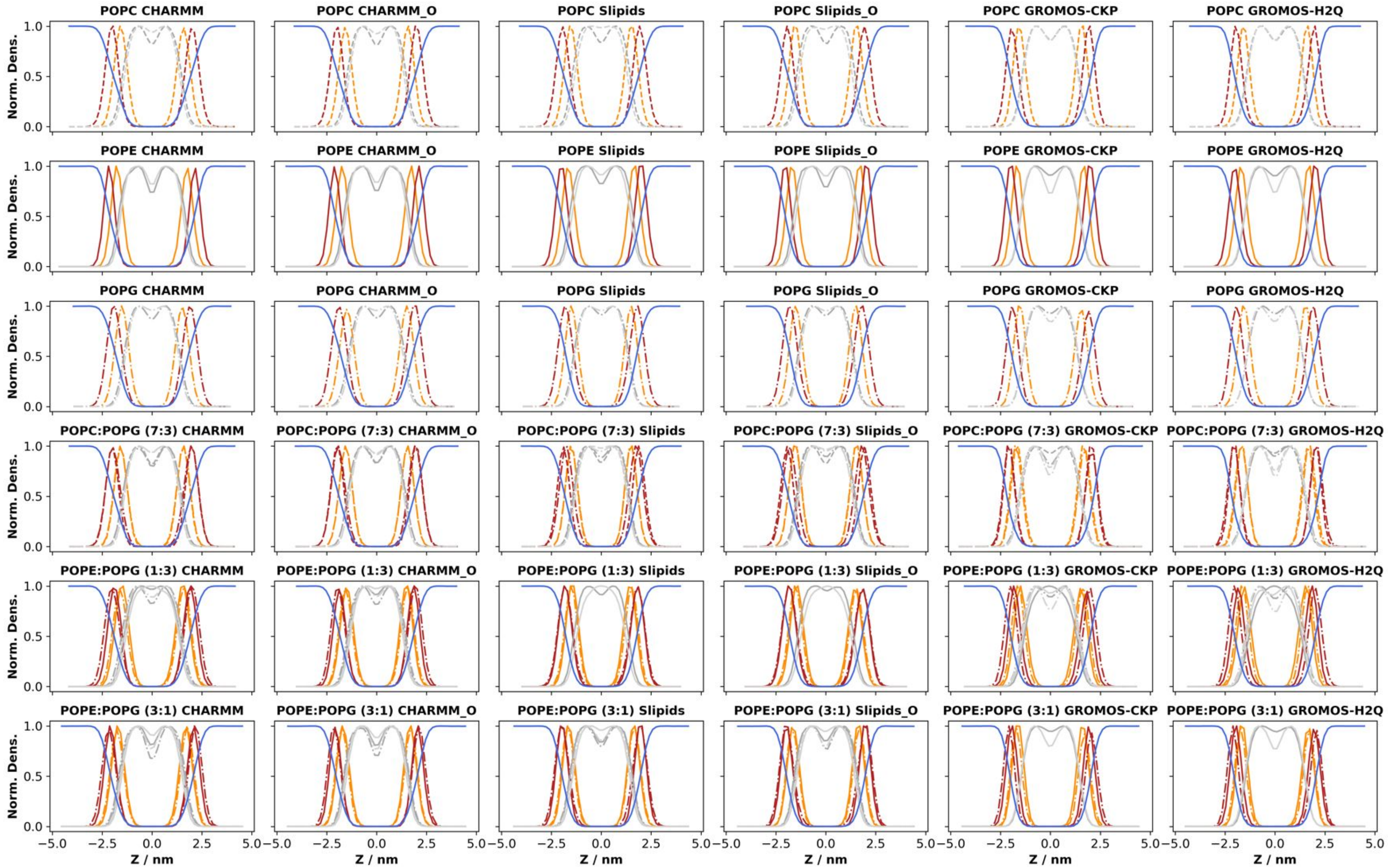

Figure S9. Lateral density distributions along the bilayer normal, z, calculated for various components of all the membrane along the last 100 ns of the simulation, for all the forcefields and all the lipid compositions. Water (blue), headgroups (red), glycerol (yellow), tail *sn1* (grey, solid line), tail *sn2* (grey, dotted line).

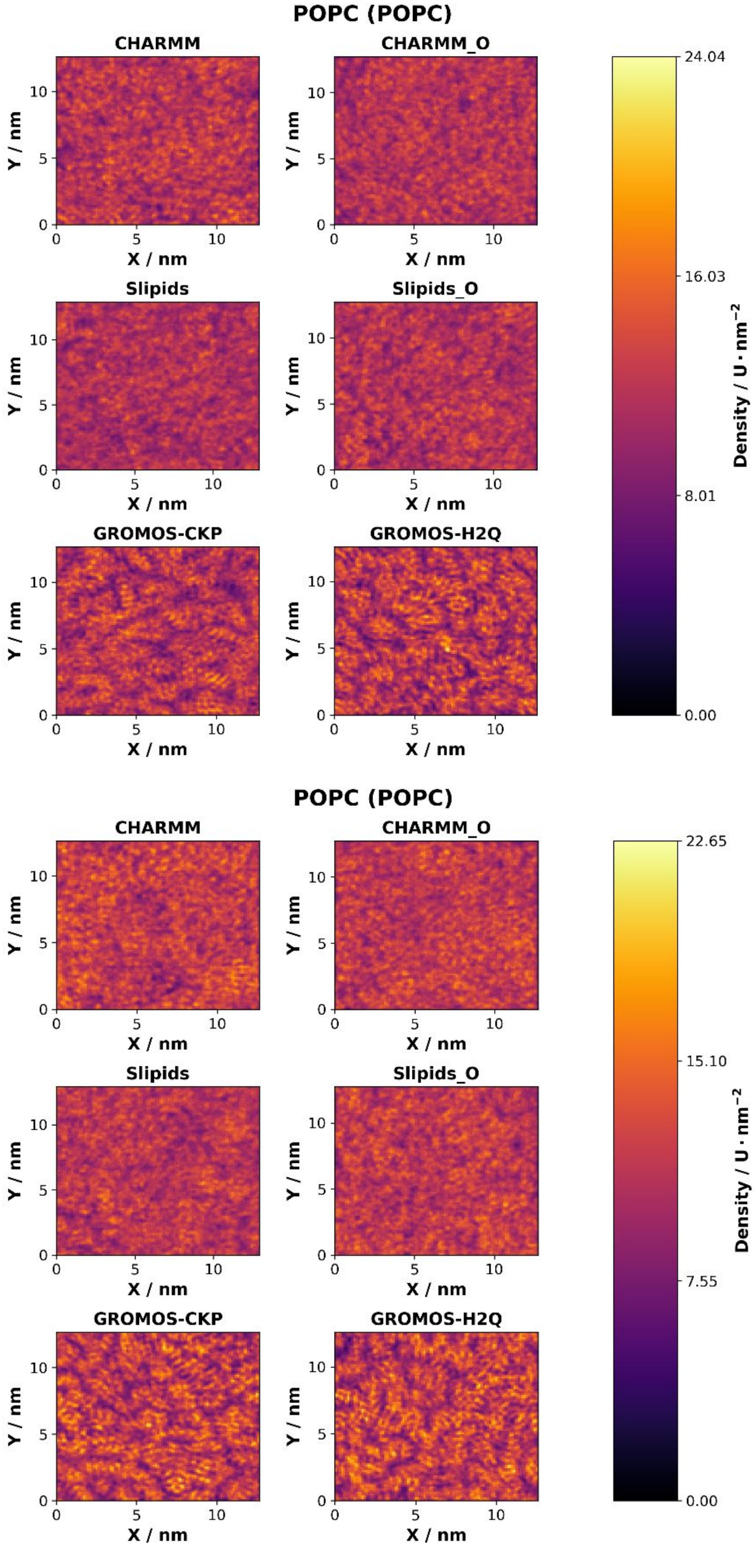

Figure S10. 2D-densities for POPC membrane, calculated for the upper (top graphs) and lower (bottom graphs) leaflet, averaged for the 400-410 ns interval, in bins with a width of 0.1x0.1 nm.

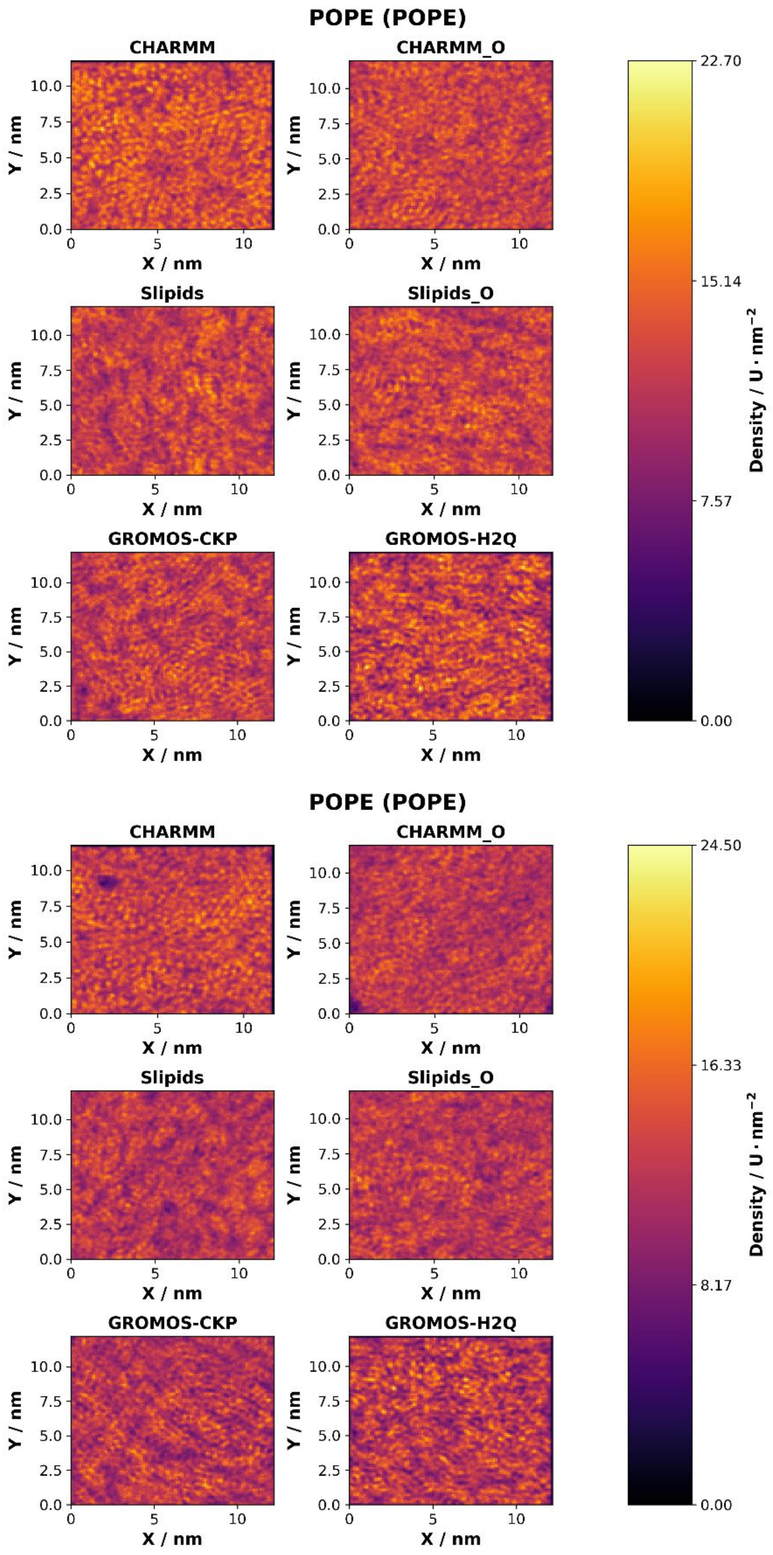

Figure S11. 2D-densities for POPE membrane, calculated for the upper (top graphs) and lower (bottom graphs) leaflet, averaged for the 400-410 ns interval, in bins with a width of 0.1x0.1 nm.

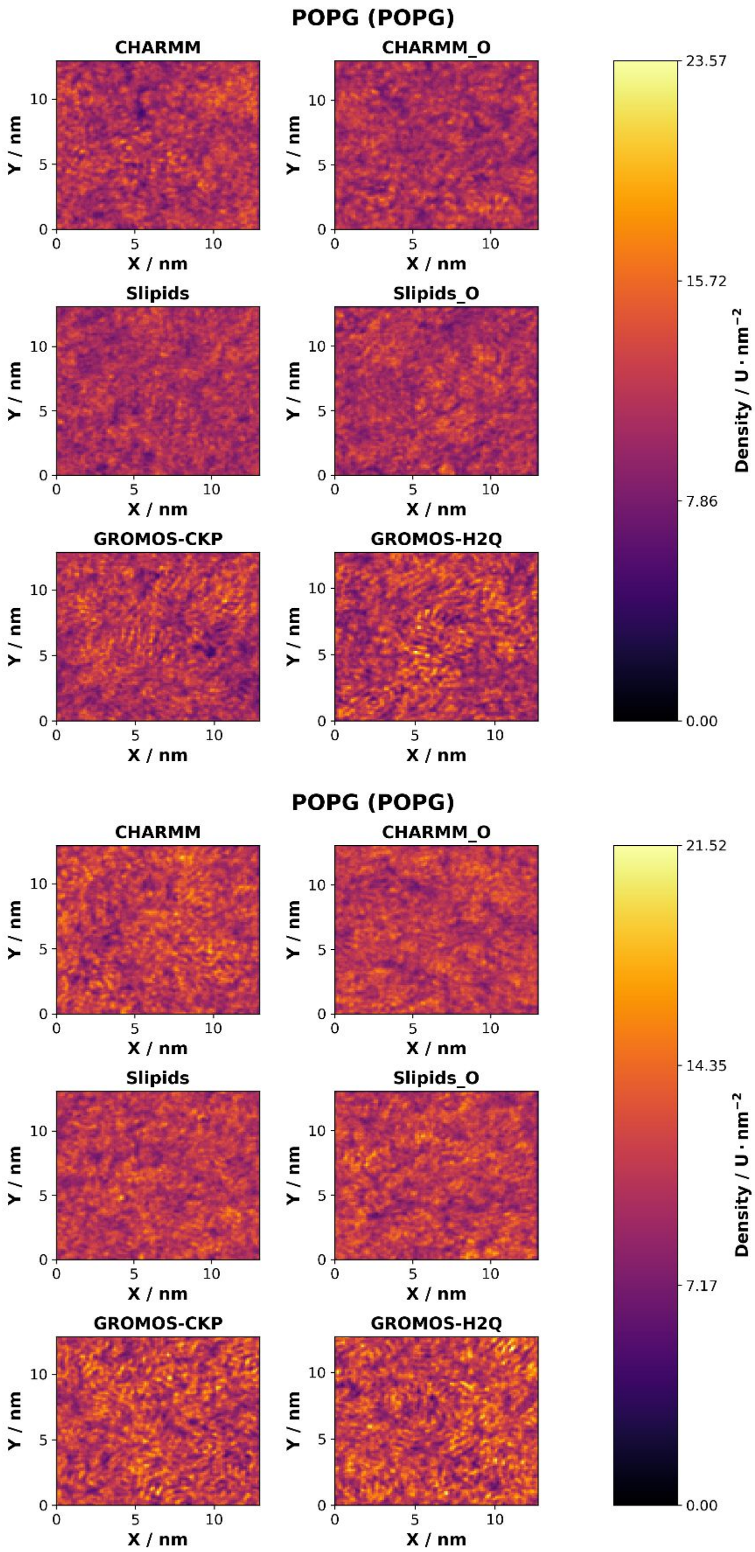

Figure S12. 2D-densities for POPG membrane, calculated for the upper (top graphs) and lower (bottom graphs) leaflet, averaged for the 400-410 ns interval, in bins with a width of 0.1x0.1 nm.

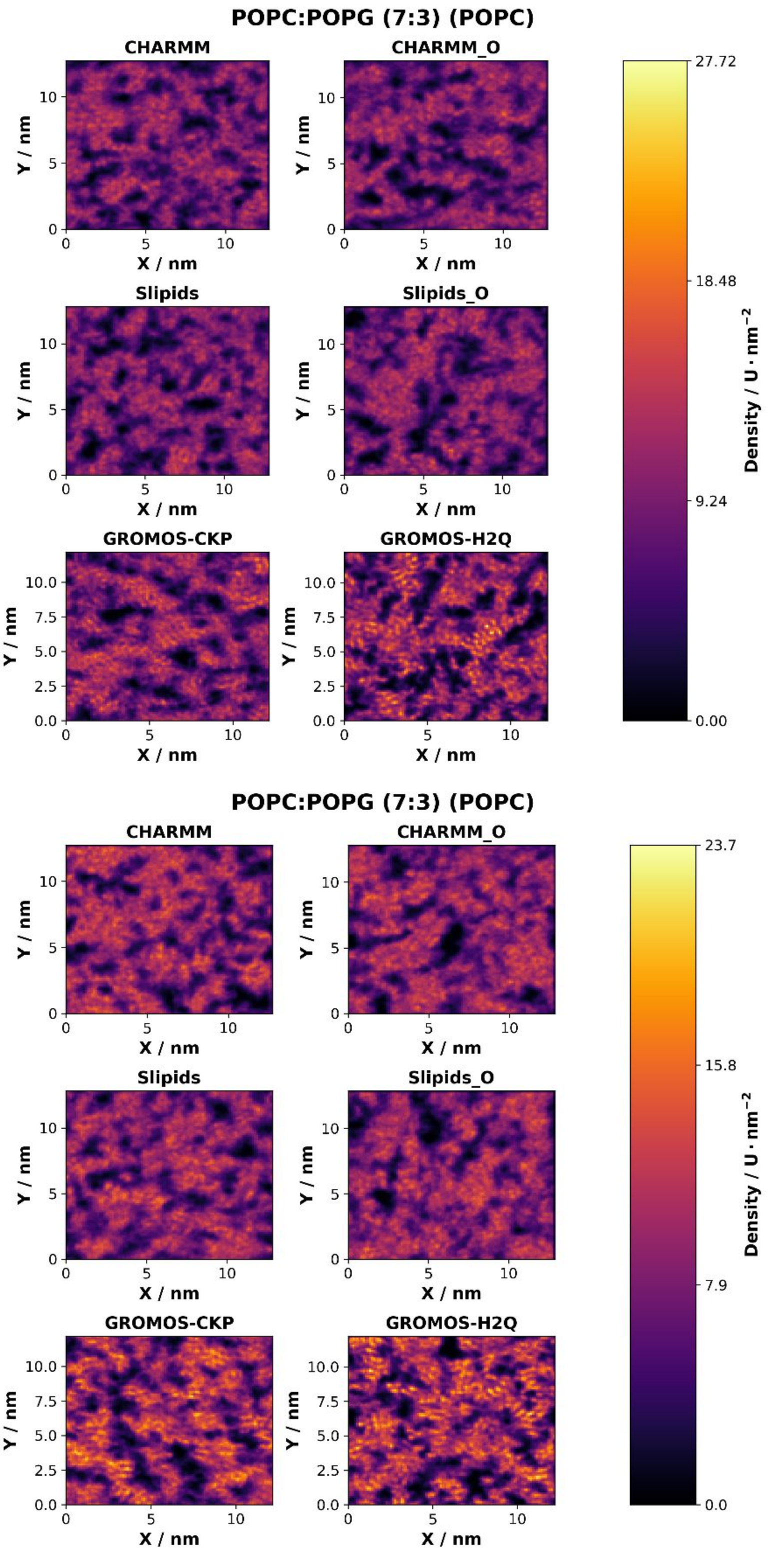

Figure S13. 2D-densities for the POPC lipid, POPC:POPG (7:3) membrane, calculated for the upper (top graphs) and lower (bottom graphs) leaflet, averaged for the 400-410 ns interval, in bins with a width of 0.1x0.1 nm.

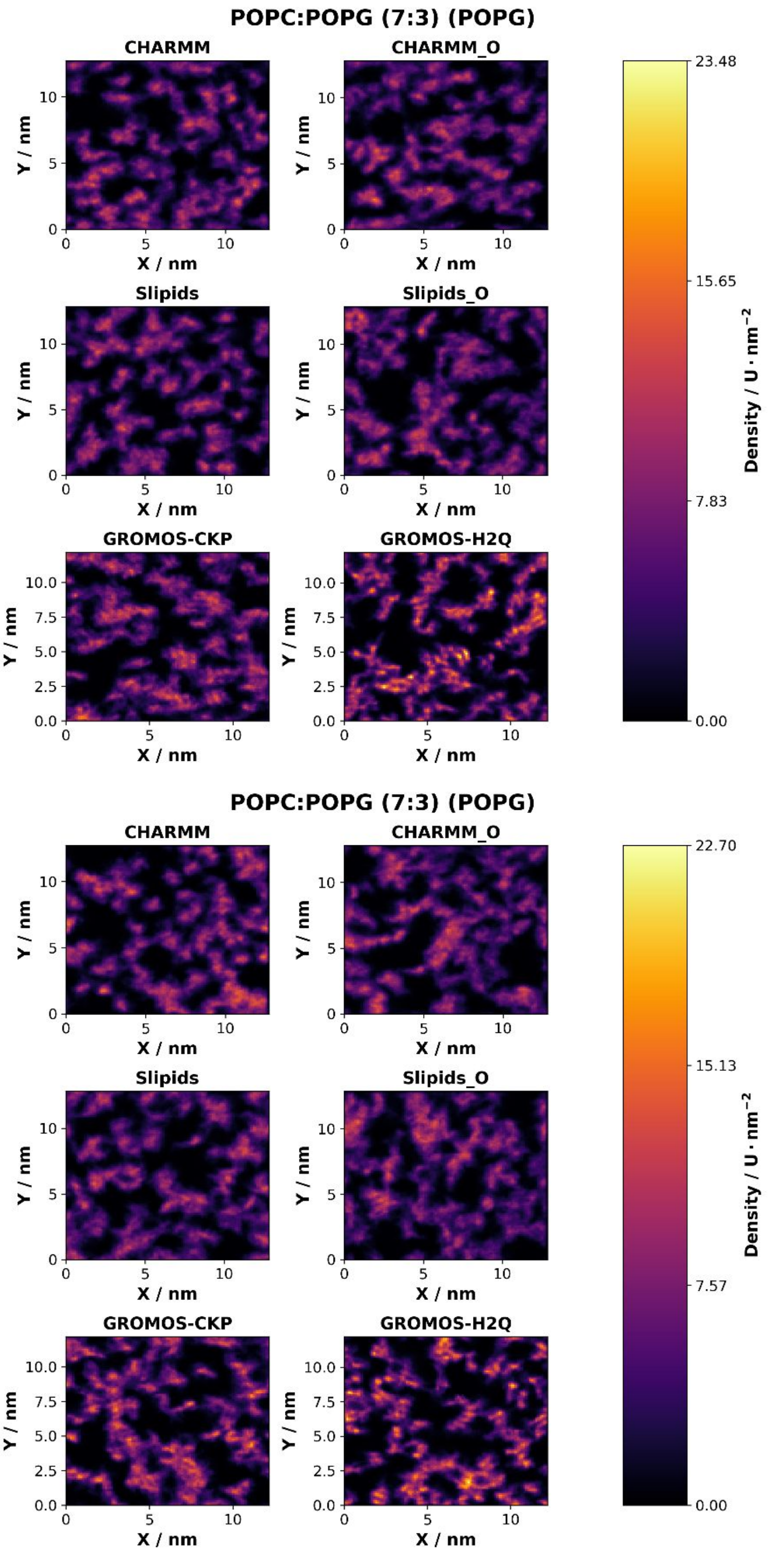

Figure S14. 2D-densities for the POPG lipid, POPC:POPG (7:3) membrane, calculated for the upper (top graphs) and lower (bottom graphs) leaflet, averaged for the 400-410 ns interval, in bins with a width of 0.1x0.1 nm.

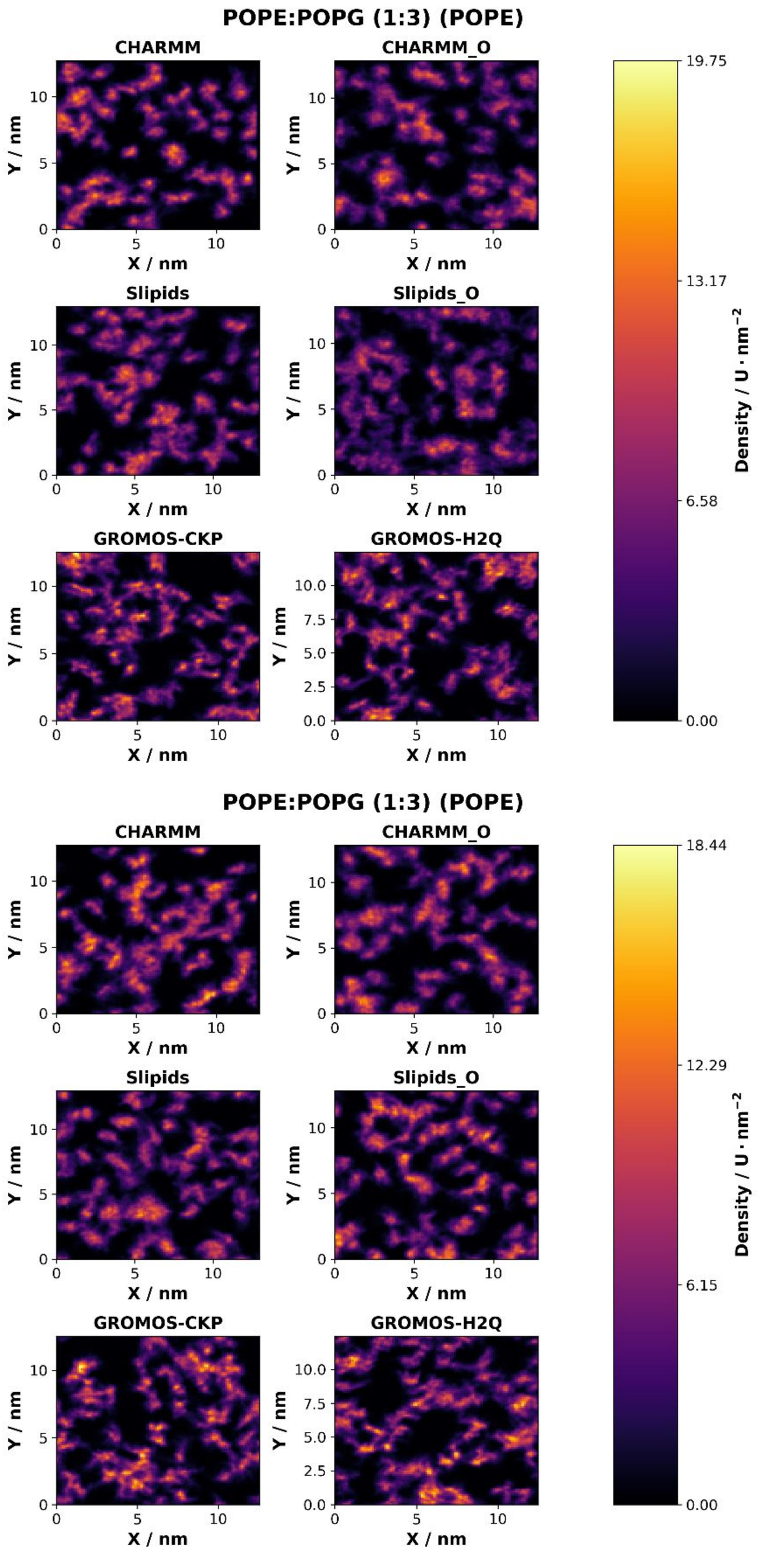

Figure S15. 2D-densities for the POPE lipid, POPE:POPG (1:3) membrane, calculated for the upper (top graphs) and lower (bottom graphs) leaflet, averaged for the 400-410 ns interval, in bins with a width of 0.1x0.1 nm.

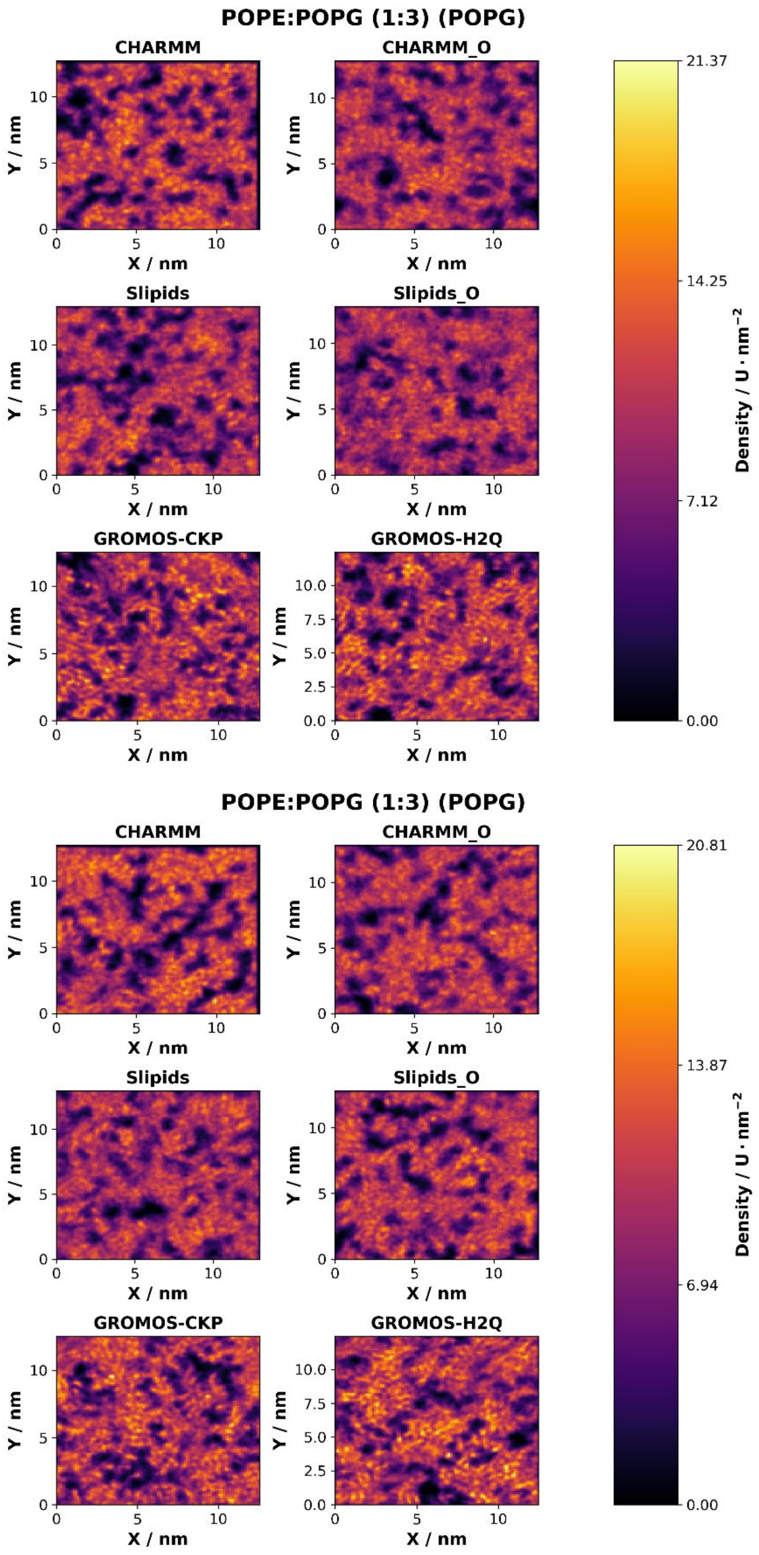

Figure S16. 2D-densities for the POPG lipid, POPE:POPG (1:3) membrane, calculated for the upper (top graphs) and lower (bottom graphs) leaflet, averaged for the 400-410 ns interval, in bins with a width of 0.1x0.1 nm.

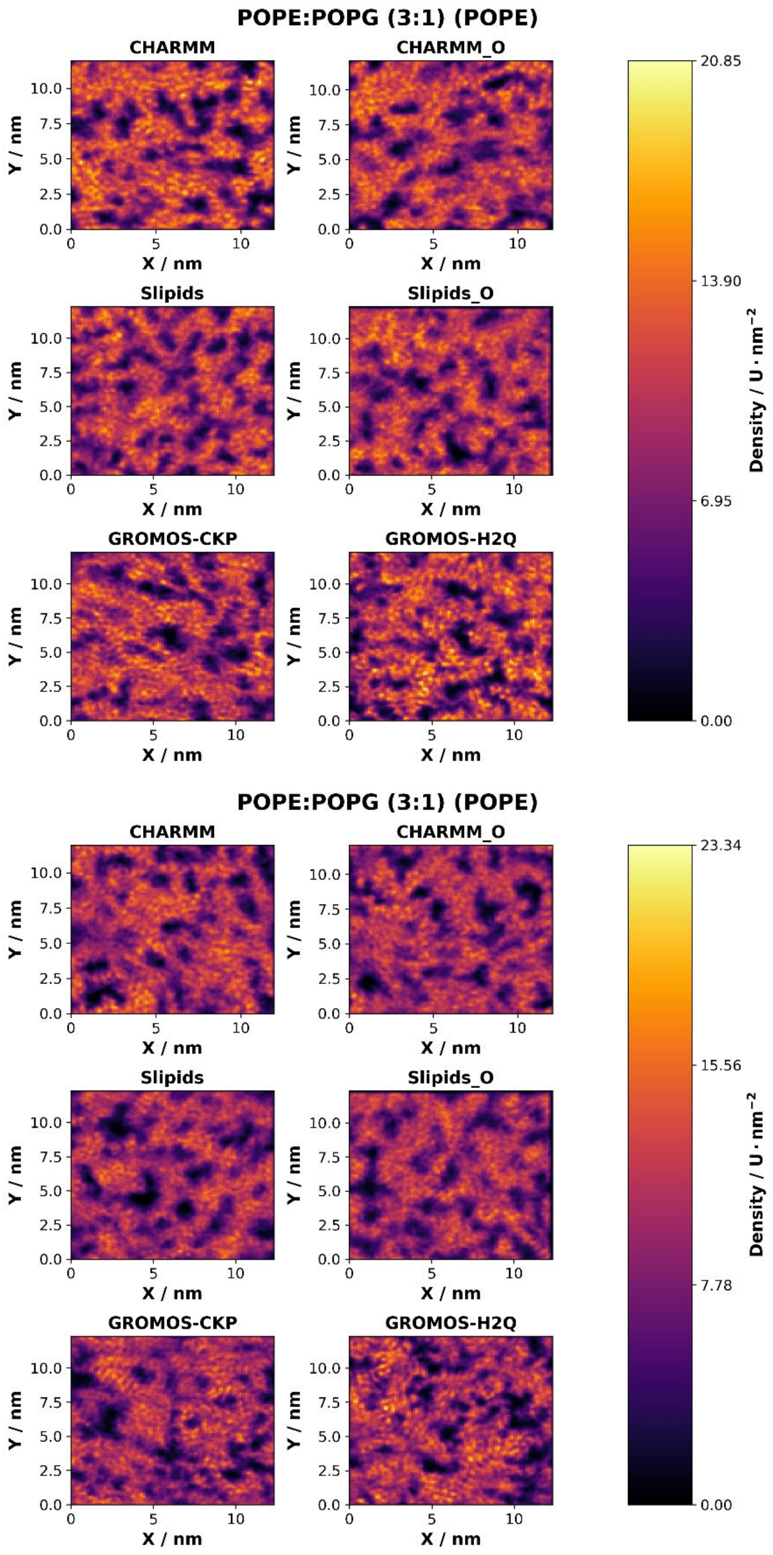

Figure S17. 2D-densities for the POPE lipid, POPE:POPG (1:3) membrane, calculated for the upper (top graphs) and lower (bottom graphs) leaflet, averaged for the 400-410 ns interval, in bins with a width of 0.1x0.1 nm.

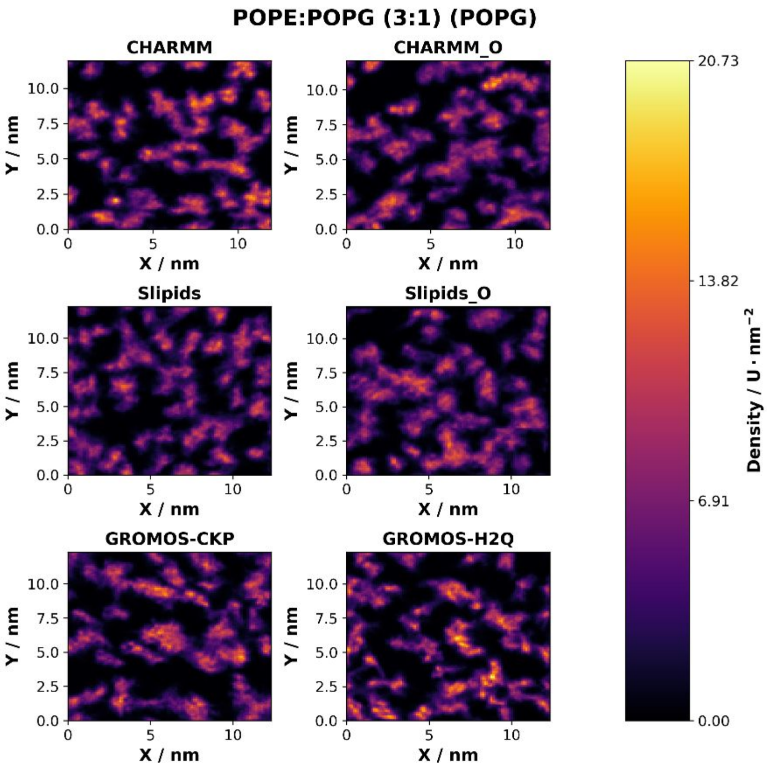

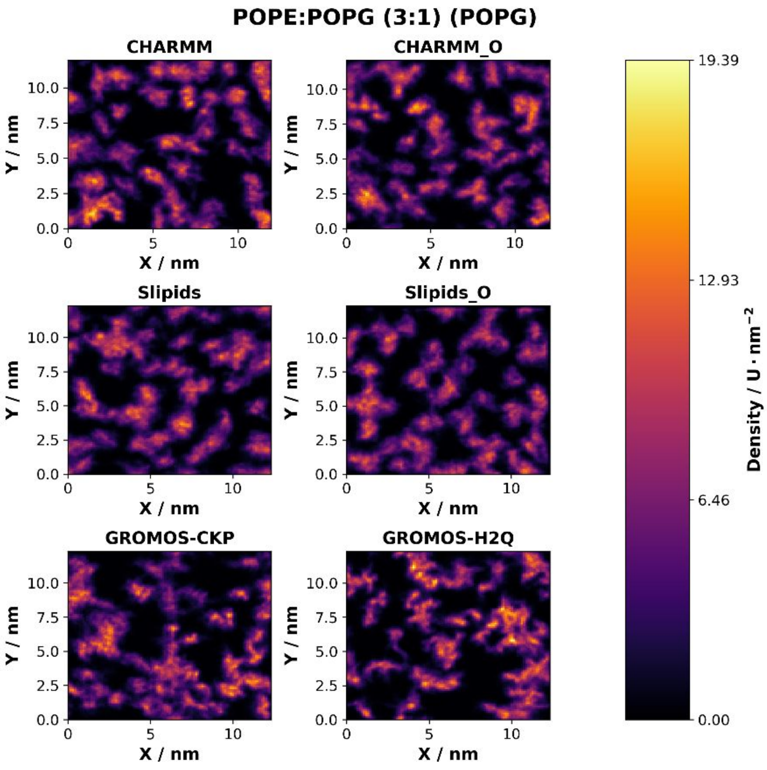

Figure S18. 2D-densities for the POPG lipid, POPE:POPG (1:3) membrane, calculated for the upper (top graphs) and lower (bottom graphs) leaflet, averaged for the 400-410 ns interval, in bins with a width of 0.1x0.1 nm.

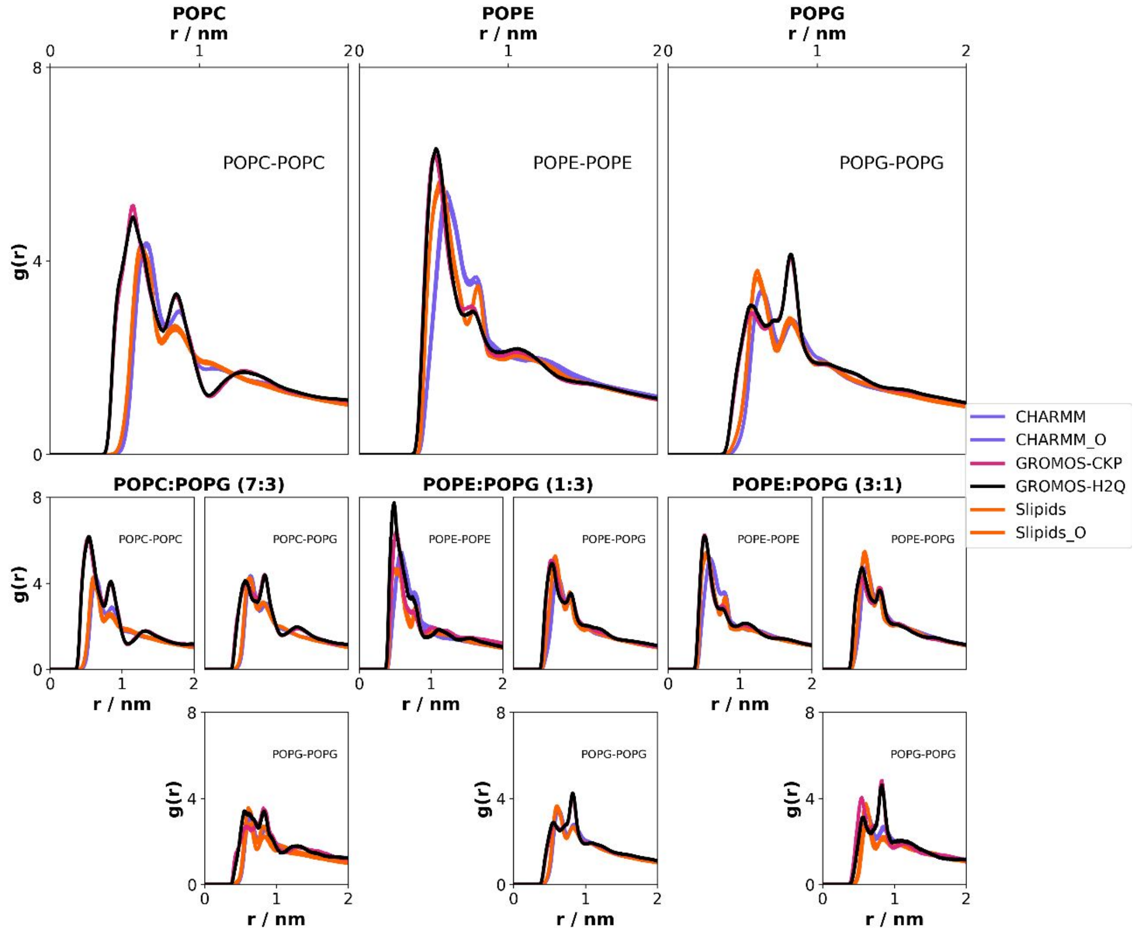

Figure S19. Comparative analysis of Radial Distribution Functions (RDFs) across different lipid compositions and forcefields: CHARMM (blue), Slipids (orange), GROMOS-CKP (magenta) and GROMOS-H2Q (black). RDFs are calculated for the phosphate groups (PO4) of lipids to examine the local structure and packing density in membranes. Top row: RDFs for pure lipid membranes of POPC (fist column), POPE (second column), and POPG (third column). Two bottom rows exhibit the RDFs for the POPC:POPG (7:3), POPE:POPG (1:3) and POPE:POPG (3:1) membranes, grouped in sets of three subgraphs. The specific RDF can be consulted in a text inset in the graphs.

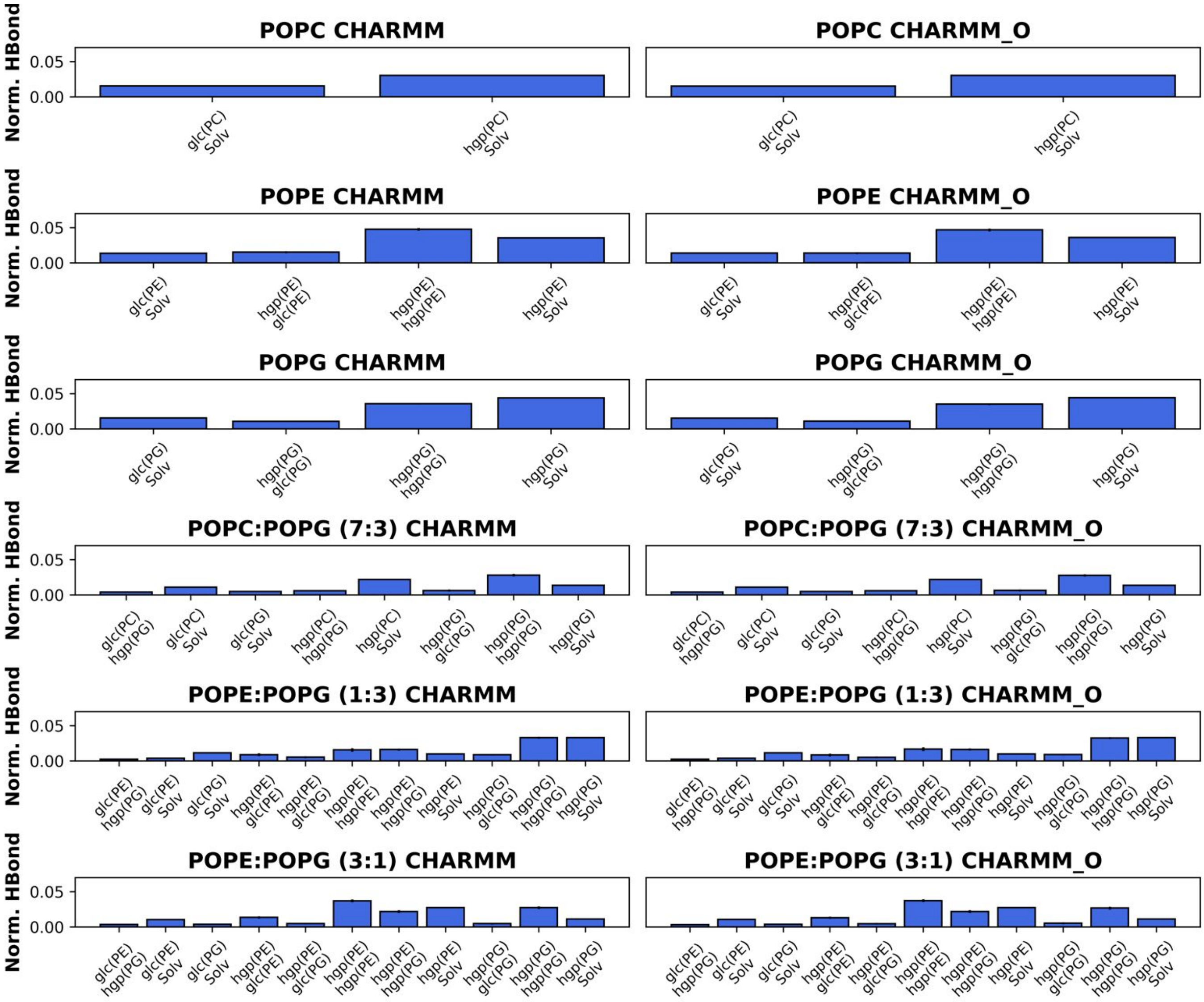

Figure S20. Normalized H-bonds between the different groups (solvent, headgroups and glycerol) of each lipid and the neighbors, along the last 100 ns of the trajectory. Each row corresponds to a different lipid composition simulated in a different force field.

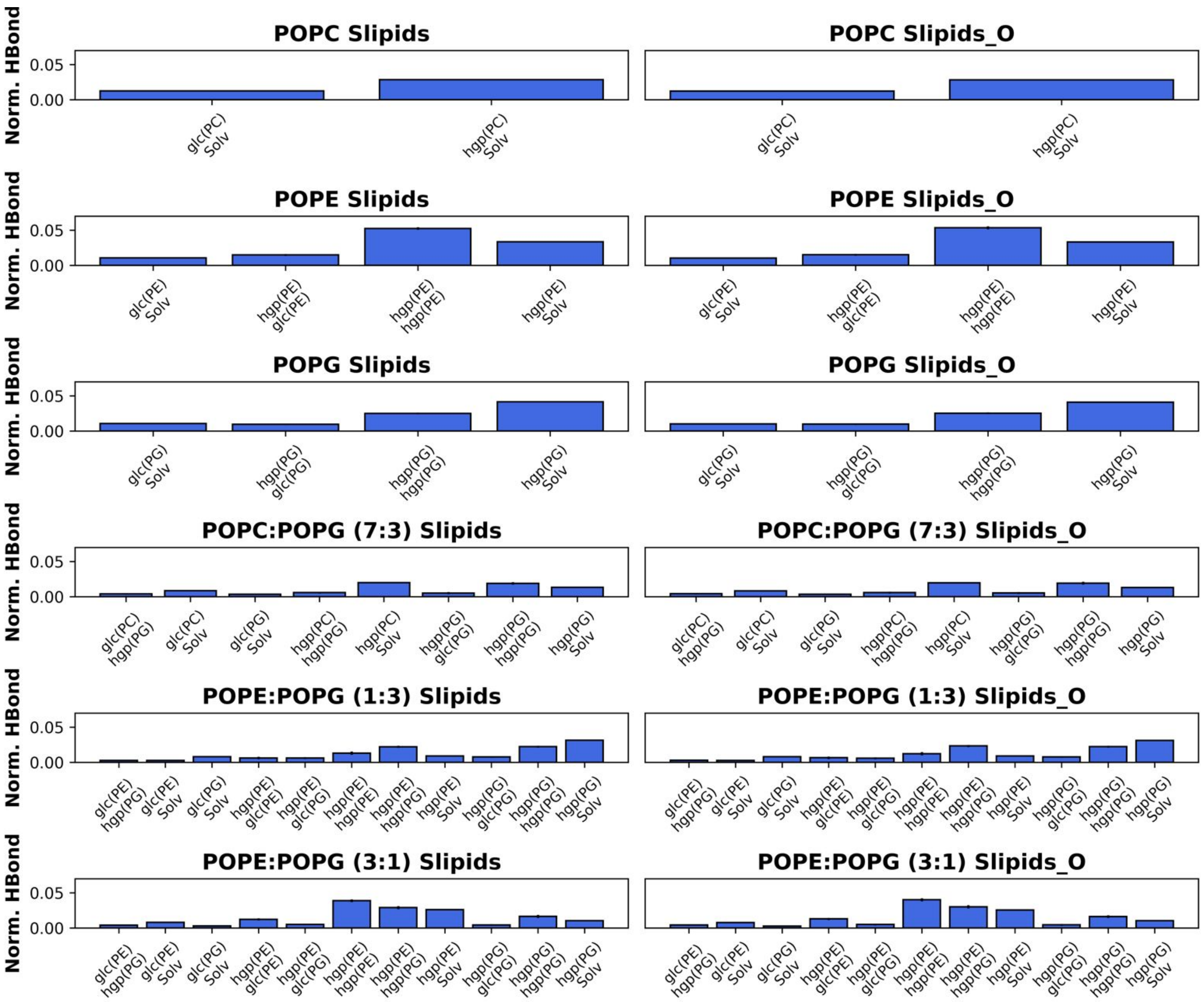

Figure S21. Normalized H-bonds between the different groups (solvent, headgroups and glycerol) of each lipid and the neighbors, along the last 100 ns of the trajectory. Each row corresponds to a different lipid composition simulated in a different force field.

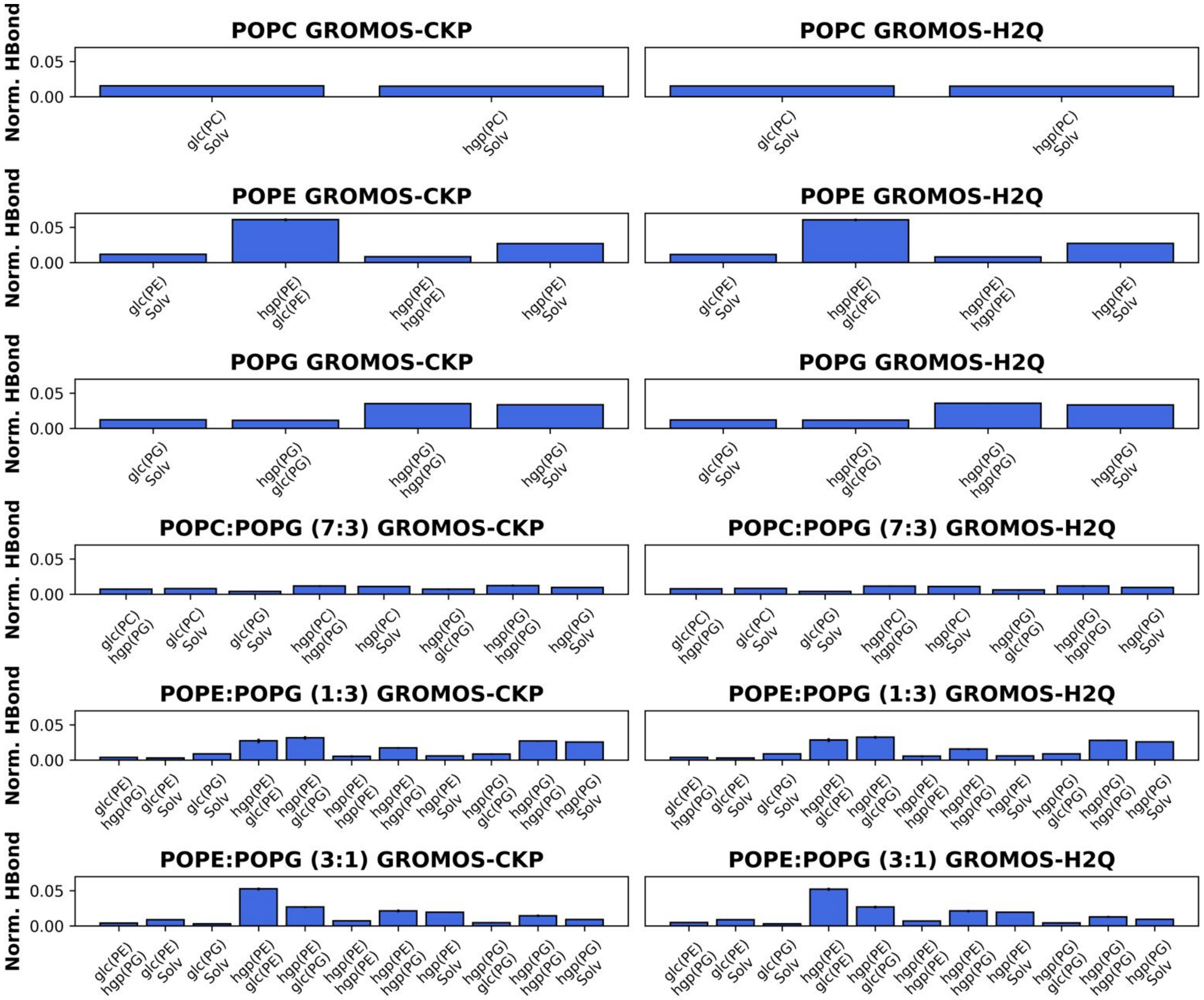

Figure S22. Normalized H-bonds between the different groups (solvent, headgroups and glycerol) of each lipid and the neighbors, along the last 100 ns of the trajectory. Each row corresponds to a different lipid composition simulated in a different force field.

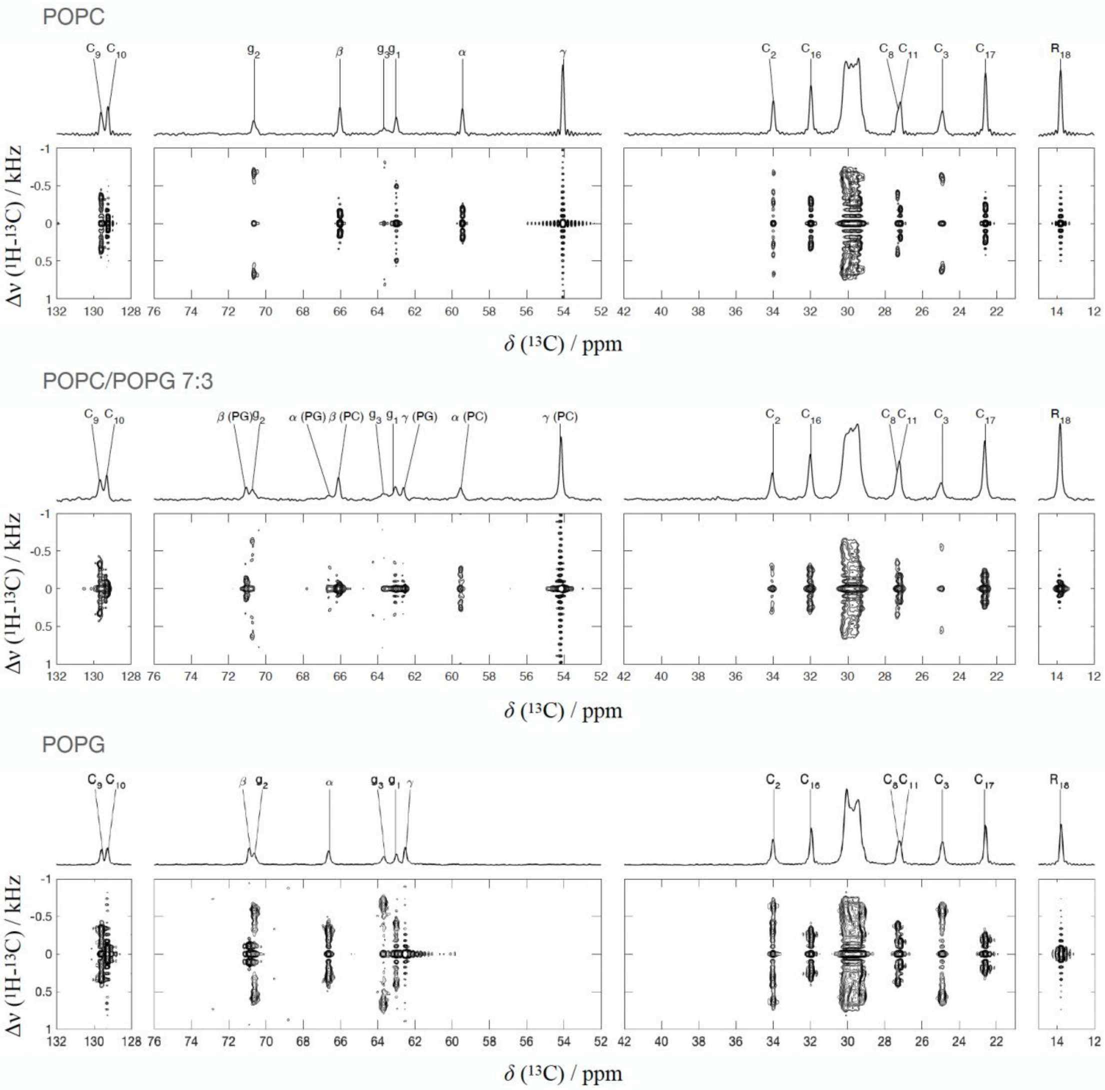

Figure S23. Selected contour plots of the R-PDLF spectra acquired from the POPC (top), POPC:POPG (middle) and POPG (bottom) samples, with carbon assignments using labels as described in the main text. The identification of the different peaks was based on previous assignments [1–3]. All the spectra were acquired with the following settings: Magic angle spinning rate of 5 kHz; Indirect dimension increments of 399.9(9) µs; 256 transients accumulated for each indirect dimension point with an acquisition time equal to approximately 50 ms; SPINAL64 ^{1}H decoupling using at an RF nutation frequencies from 30-45 kHz (lower decoupling strength was used for samples with charged lipids to prevent RF heating). Recycle delay of 5-10 s to prevent RF heating of the samples; 90º and 180º rINEPT pulses with pulse lengths of 3.2 and 6.4 µs, respectively; rINEPT delays equal to 1.2 and 2 ms; The external magnetic field used corresponded to a Larmor frequency for 1H of approximately 400 MHz. The ^{13}C chemical shifts were calibrated by setting the methyl ^{13}C chemical shift to 13.8 ppm.

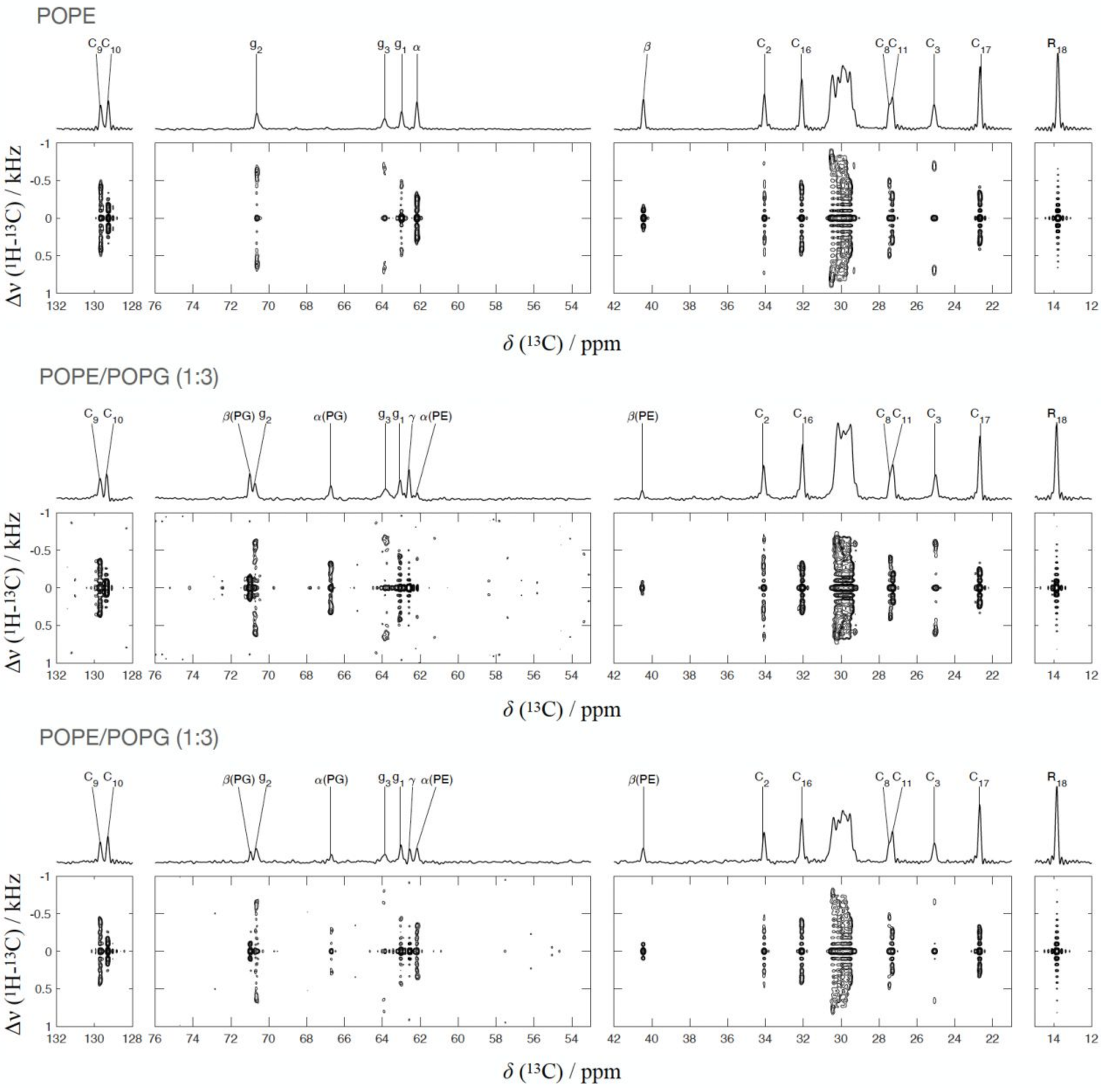

Figure S24. Selected contour plots of the R-PDLF spectra acquired from the POPE (top), POPE:POPG (1:3) (middle) and POPE:POPG (3:1) (bottom) samples, with carbon assignments using labels as described in the main text. The identification of the different peaks were based on previous assignments [1–3]. All the spectra were acquired with the settings described in the previous Figure caption.

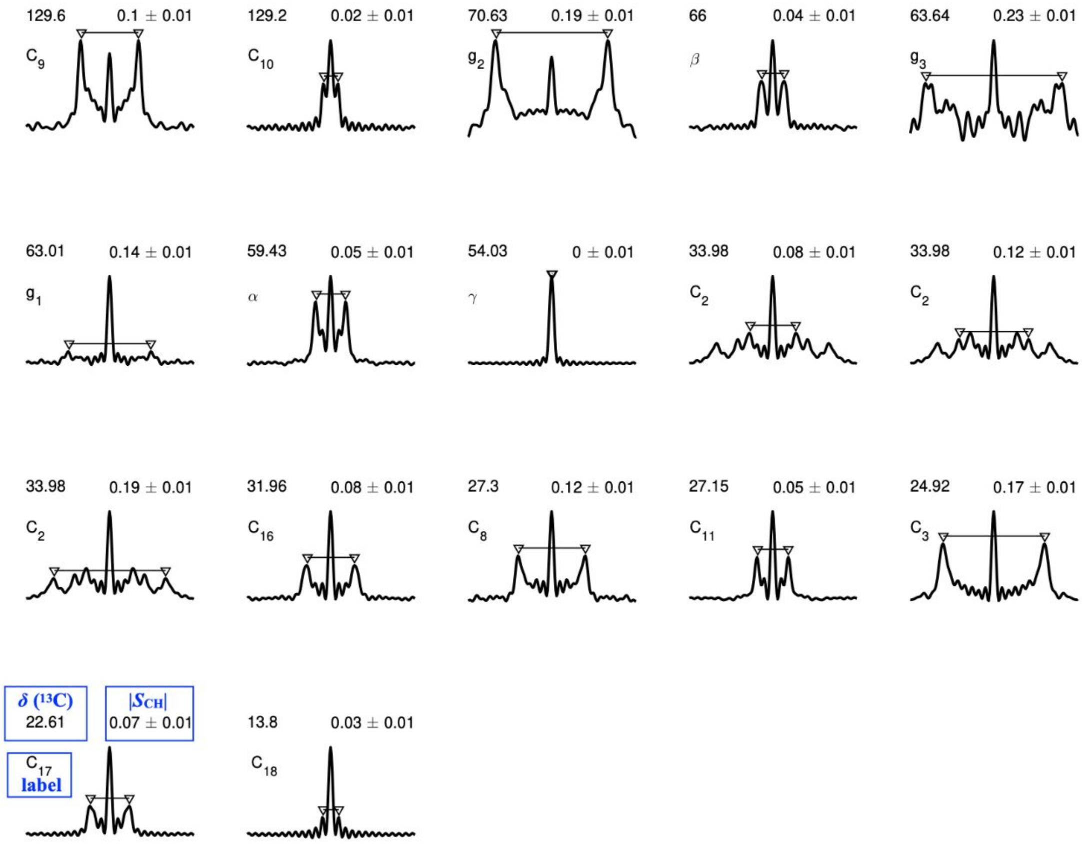

Figure S25. Selected dipolar slices showing the dipolar splittings used to calculate the different order parameters of the R-PDLF spectra acquired from the POPC sample, with carbon assignments using labels as described in the main text.

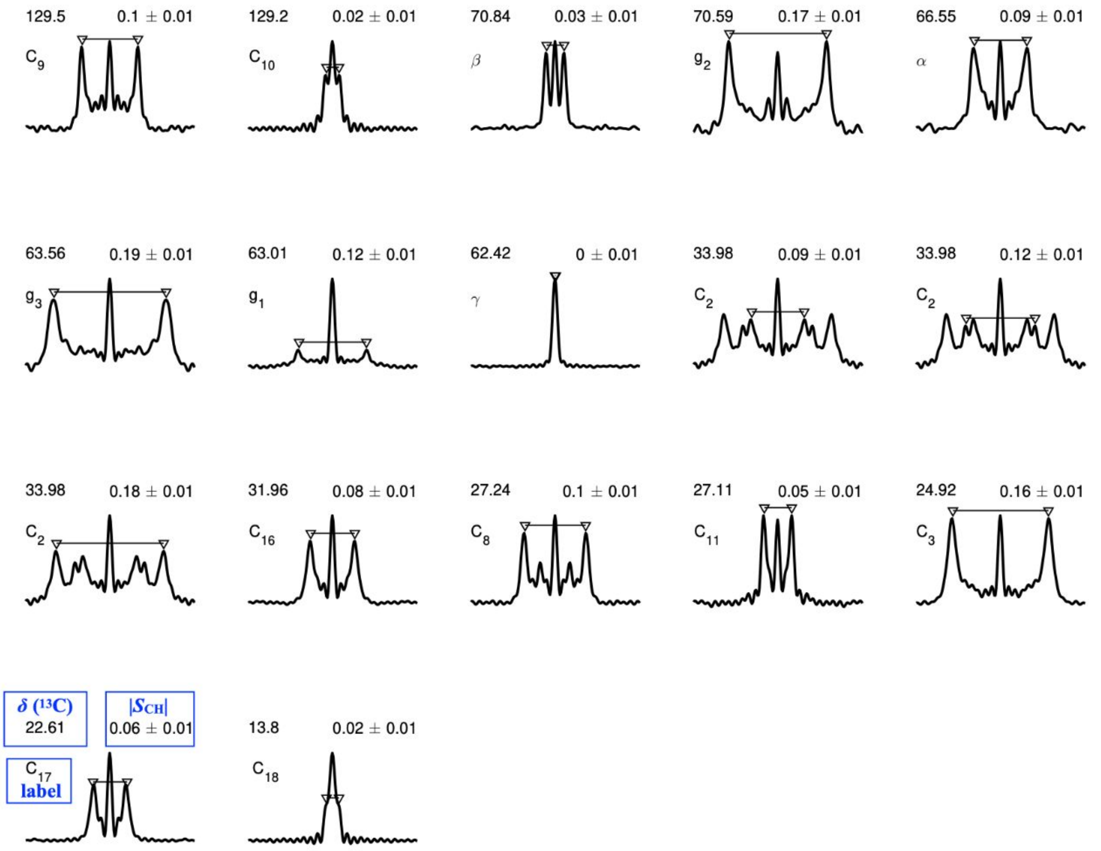

Figure S26. Selected dipolar slices showing the dipolar splittings used to calculate the different order parameters of the R-PDLF spectra acquired from the POPG sample, with carbon assignments using labels as described in the main text.

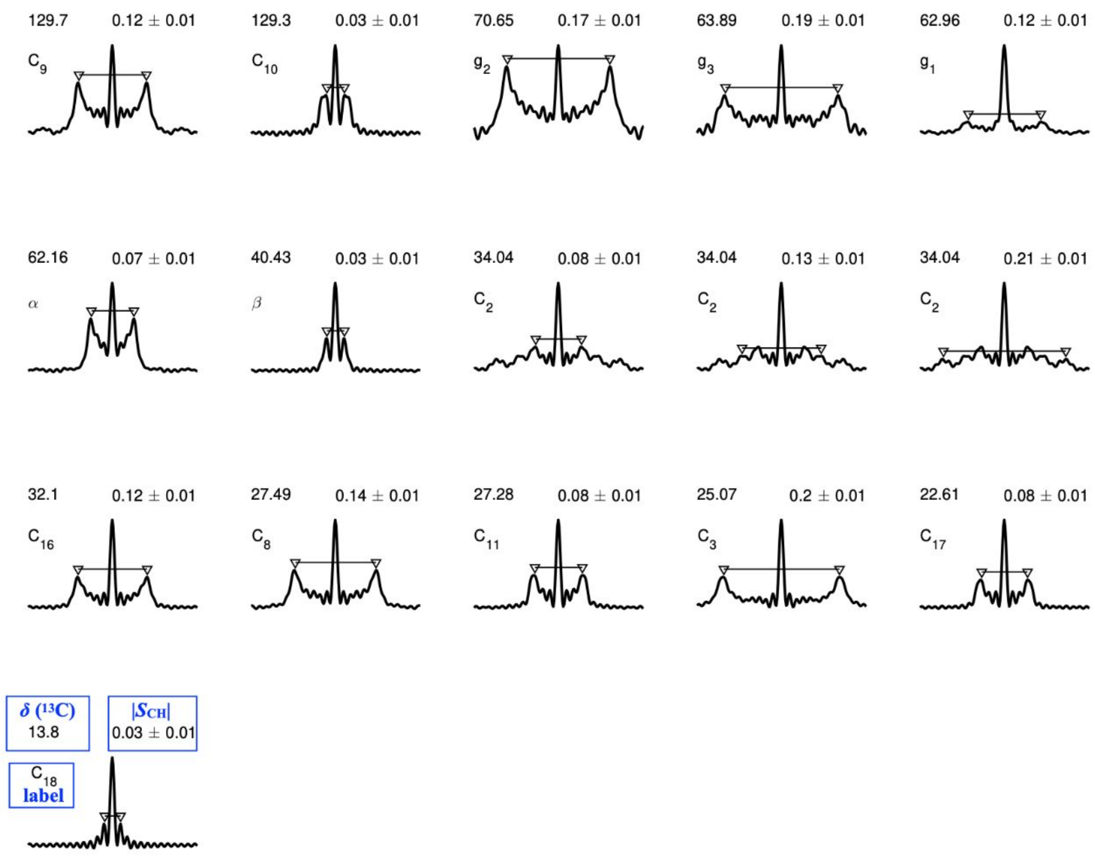

Figure S27. Selected dipolar slices showing the dipolar splittings used to calculate the different order parameters of the R-PDLF spectra acquired from the POPE sample, with carbon assignments using labels as described in the main text.

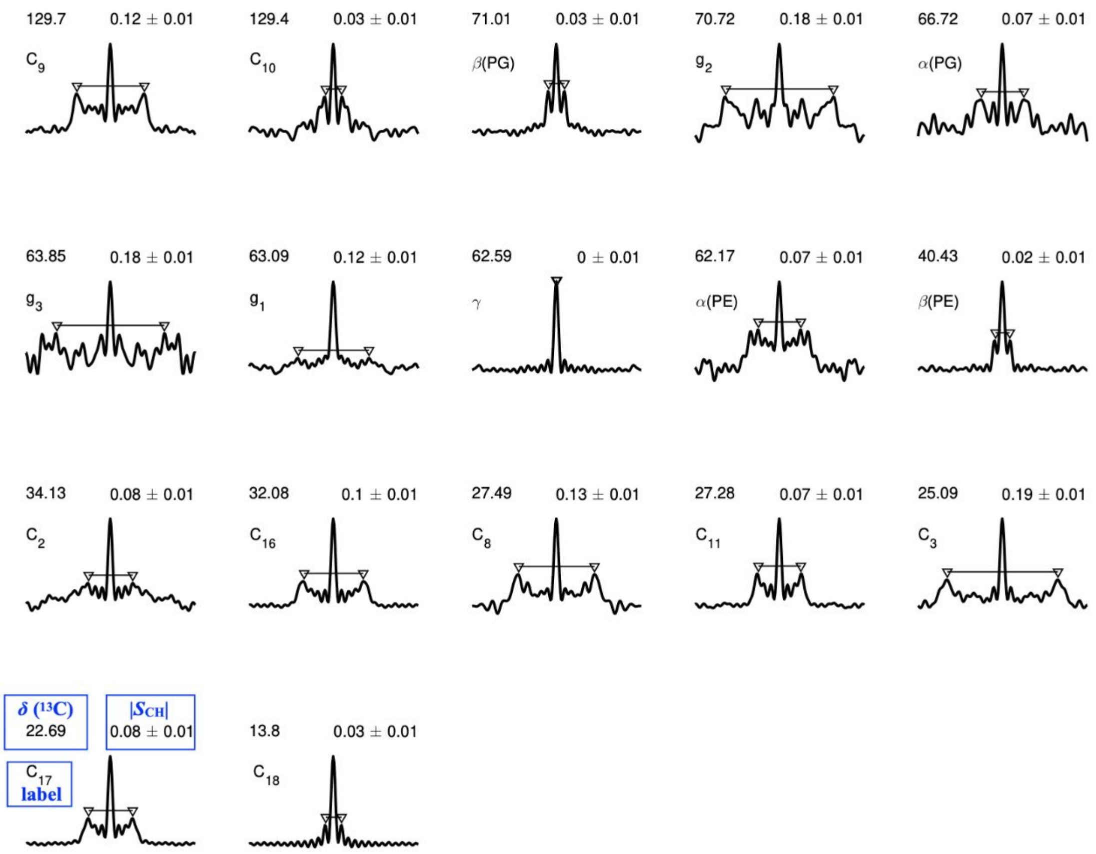

Figure S28. Selected dipolar slices showing the dipolar splittings used to calculate the different order parameters of the R-PDLF spectra acquired from the POPE:POPG (3:1) sample, with carbon assignments using labels as described in the main text.

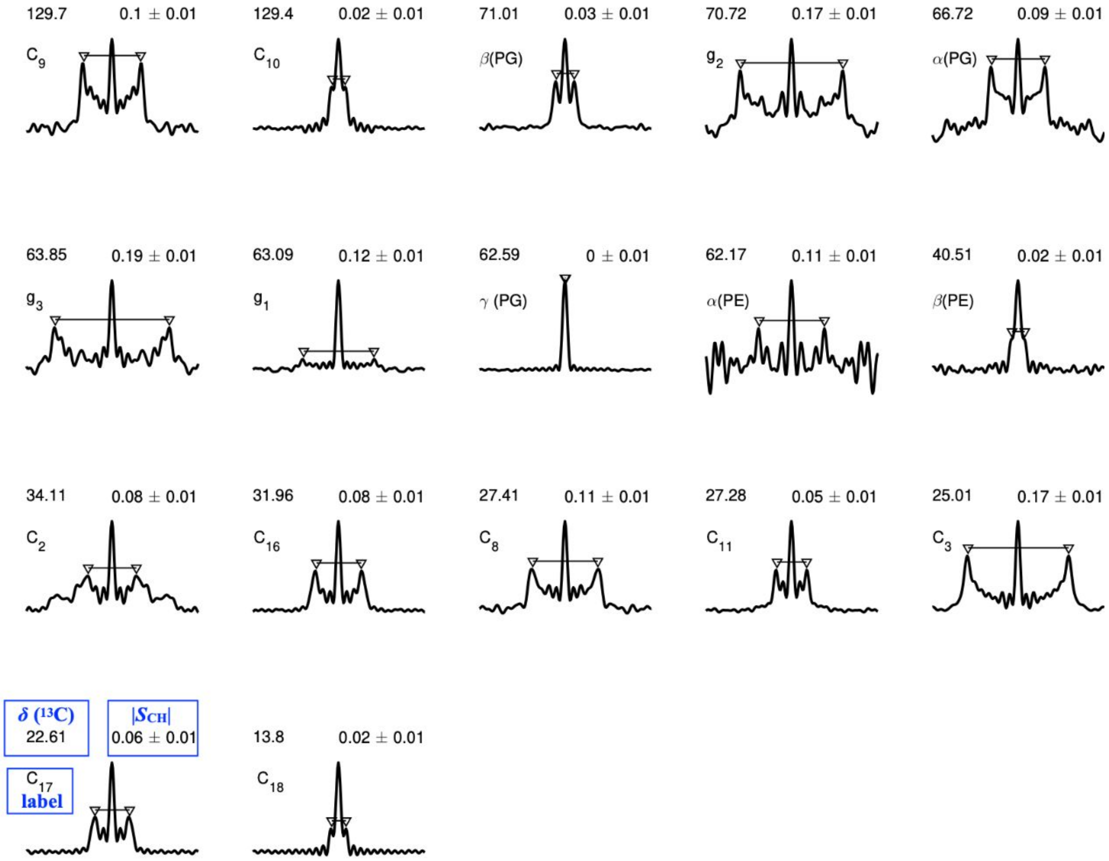

Figure S29. Selected dipolar slices showing the dipolar splittings used to calculate the different order parameters of the R-PDLF spectra acquired from the POPE:POPG (1:3) sample, with carbon assignments using labels as described in the main text.

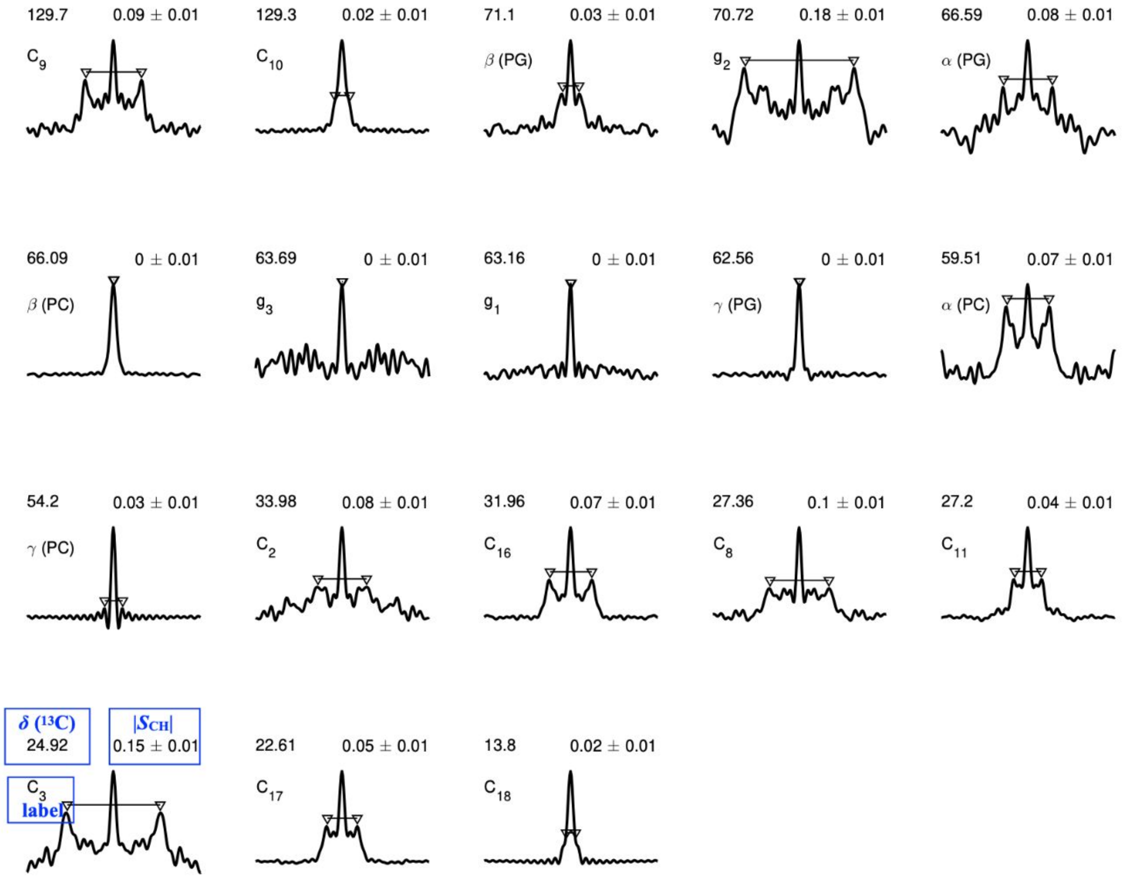

Figure S30. Selected dipolar slices showing the dipolar splittings used to calculate the different order parameters of the R-PDLF spectra acquired from the POPC:POPG (7:3) sample, with carbon assignments using labels as described in the main text.

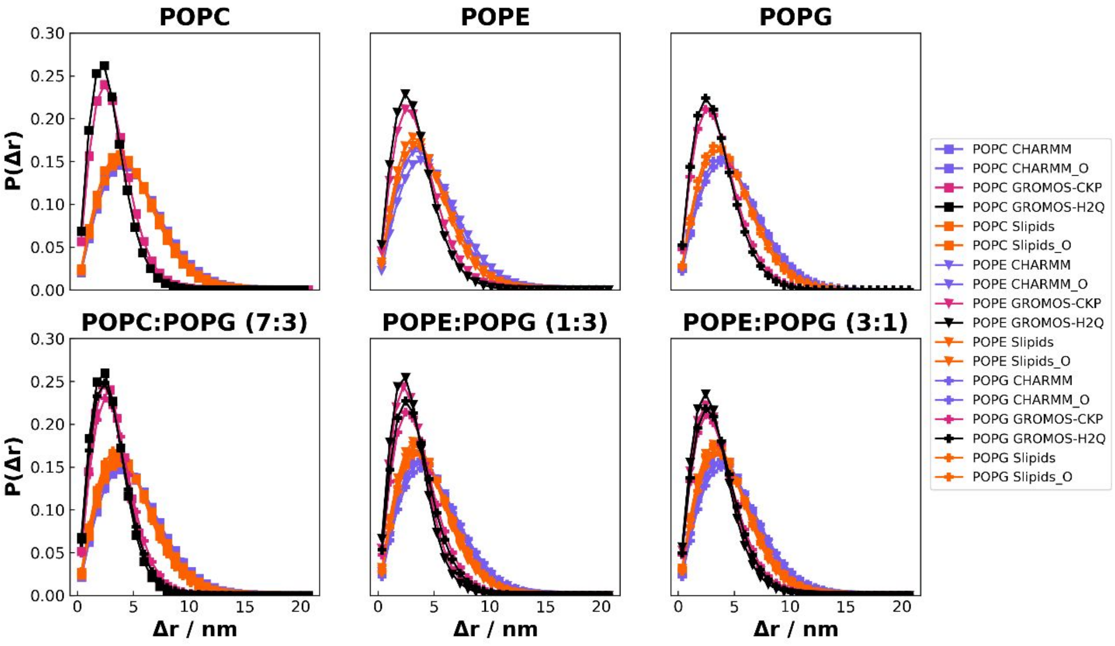

Figure S31. Probability density for the lateral displacement for each lipid type in a time window of Δt = 2 ns, calculated along the last 100 ns of the trajectory. Marker types differentiate specific lipids (square for POPC, triangle for POPE and plus for POPG), and colour indicates force field (violet for CHARMM, orange for Slipids, magenta for GROMOS-CKP and black for GROMOS-H2Q).

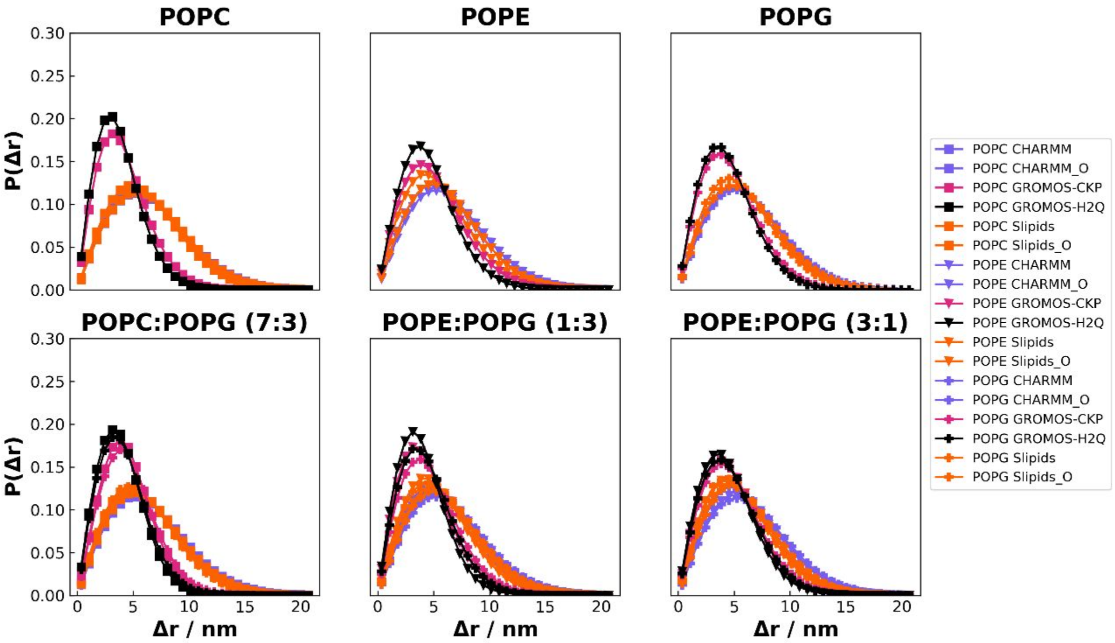

Figure S32. Probability density for the lateral displacement for each lipid type in a time window of Δt = 5 ns, calculated along the last 100 ns of the trajectory. Marker types differentiate specific lipids (square for POPC, triangle for POPE and plus for POPG), and colour indicates force field (violet for CHARMM, orange for Slipids, magenta for GROMOS-CKP and black for GROMOS-H2Q).

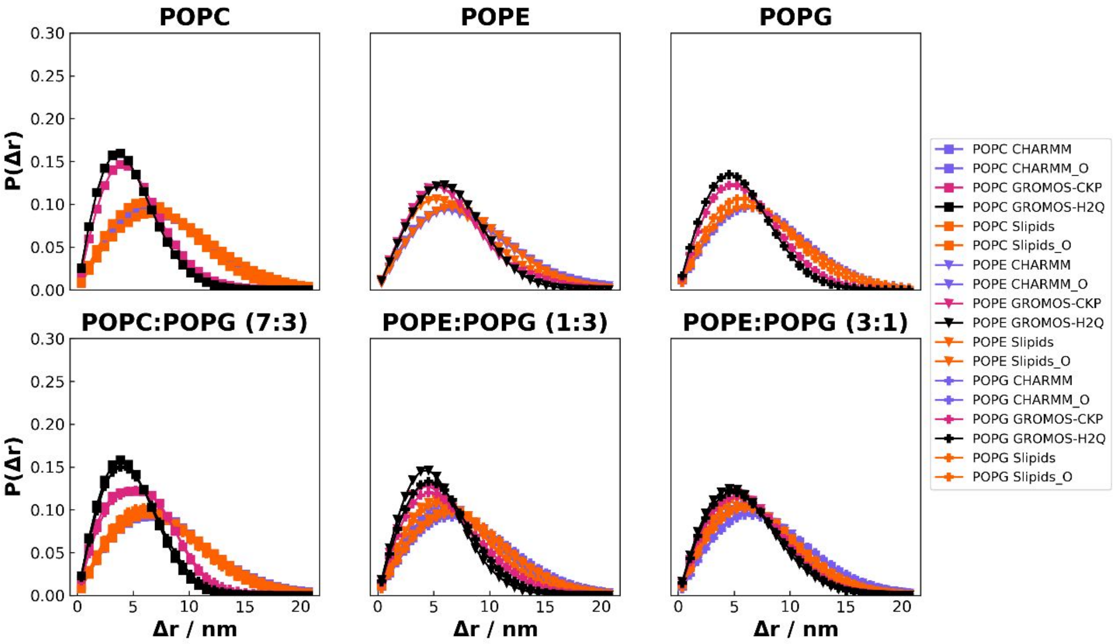

Figure S33. Probability density for the lateral displacement for each lipid type in a time window of Δt = 10 ns, calculated along the last 100 ns of the trajectory. Marker types differentiate specific lipids (square for POPC, triangle for POPE and plus for POPG), and colour indicates force field (violet for CHARMM, orange for Slipids, magenta for GROMOS-CKP and black for GROMOS-H2Q).

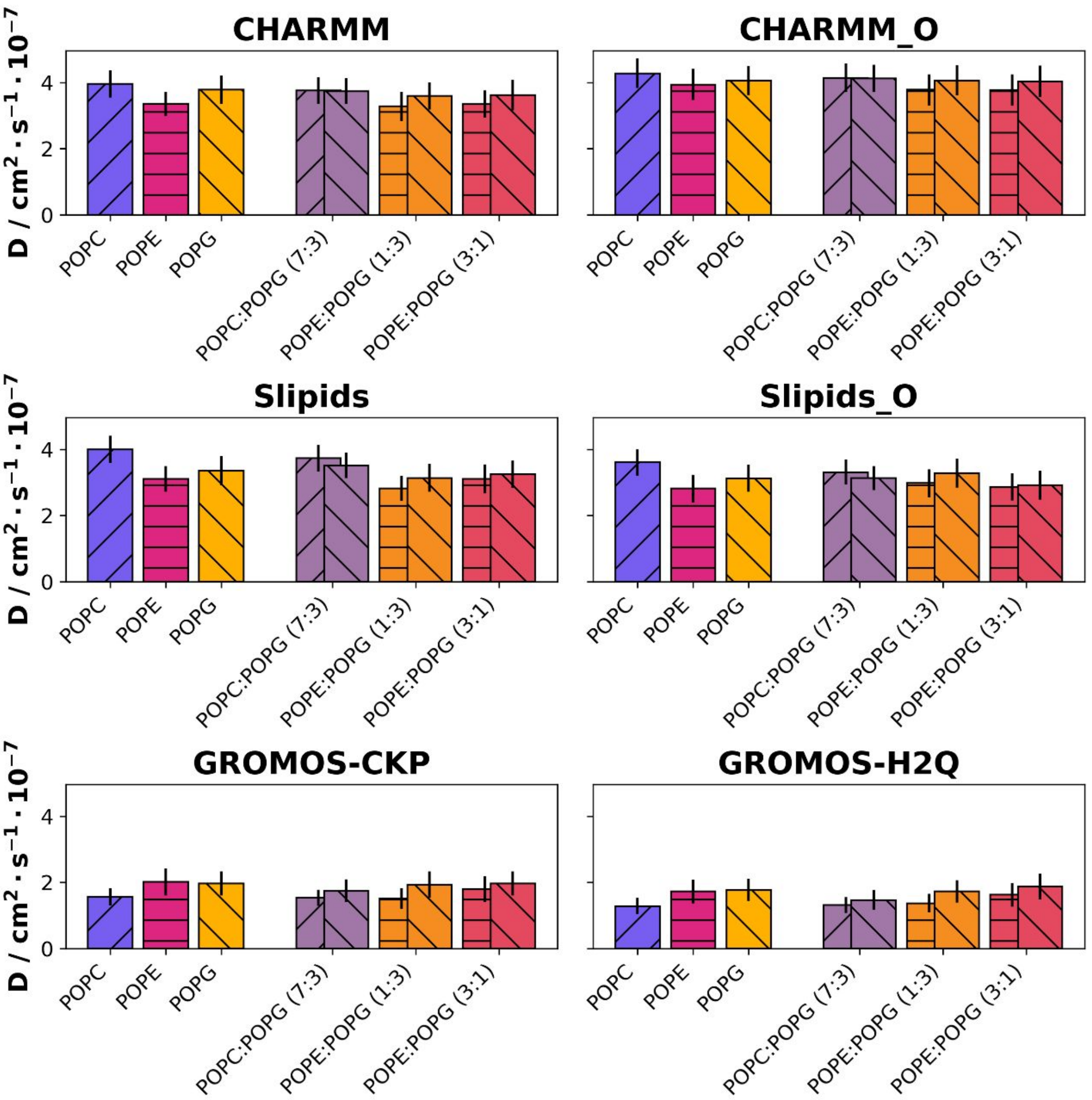

Figure S34. Bar plots from Figure S30, for a time window of $\Delta t = 2$ ns. The hatching represents the specific lipid type: forward slash – POPC, horizontal slash – POPE, backward slash – POPG. The errorbar represents $\pm \sigma$.

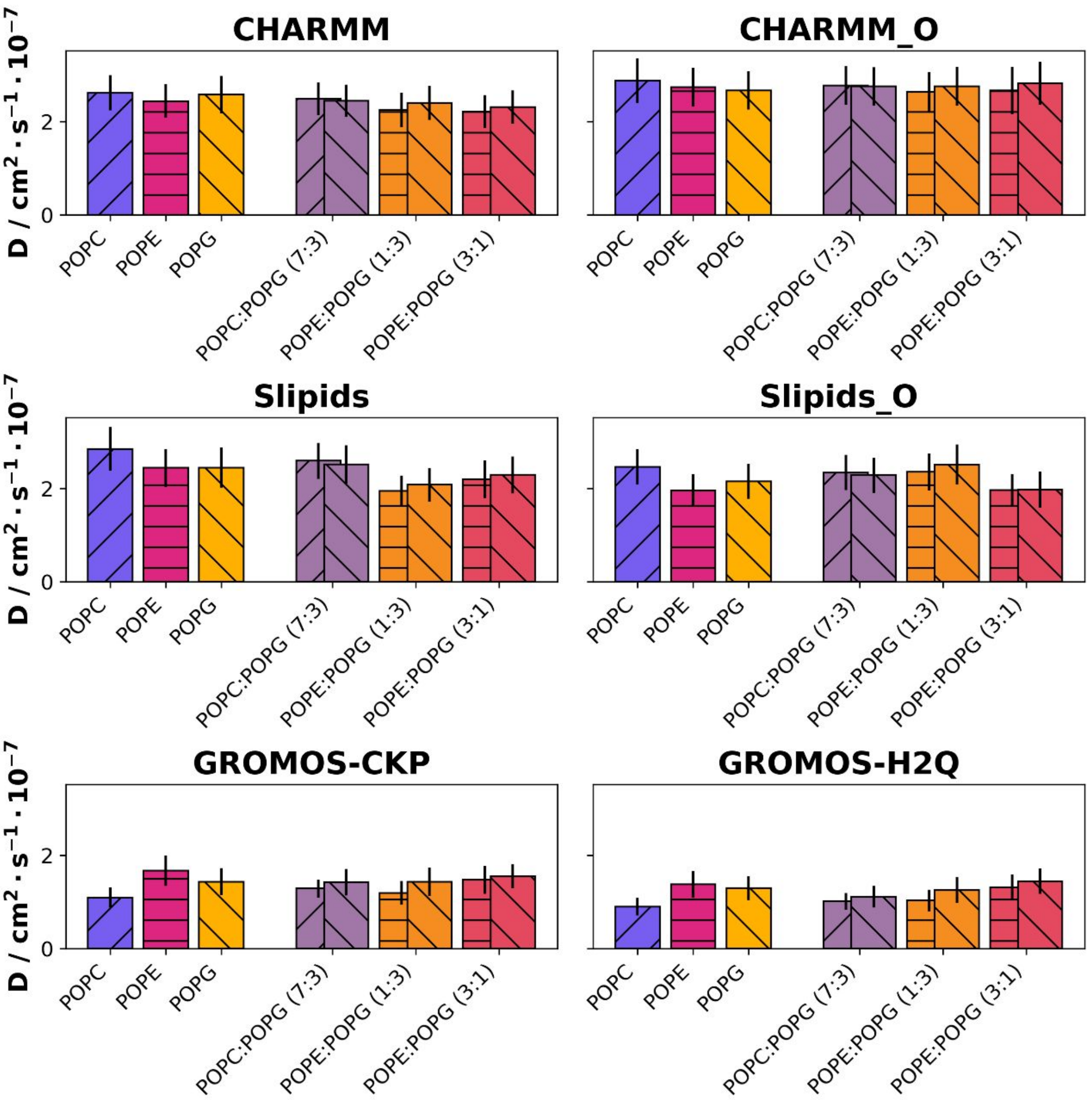

Figure S35. Bar plots from Figure S31, for a time window of t = 5 ns. The hatching represents the specific lipid type: forward slash – POPC, horizontal slash – POPE, backward slash – POPG. The errorbar represents ± σ.

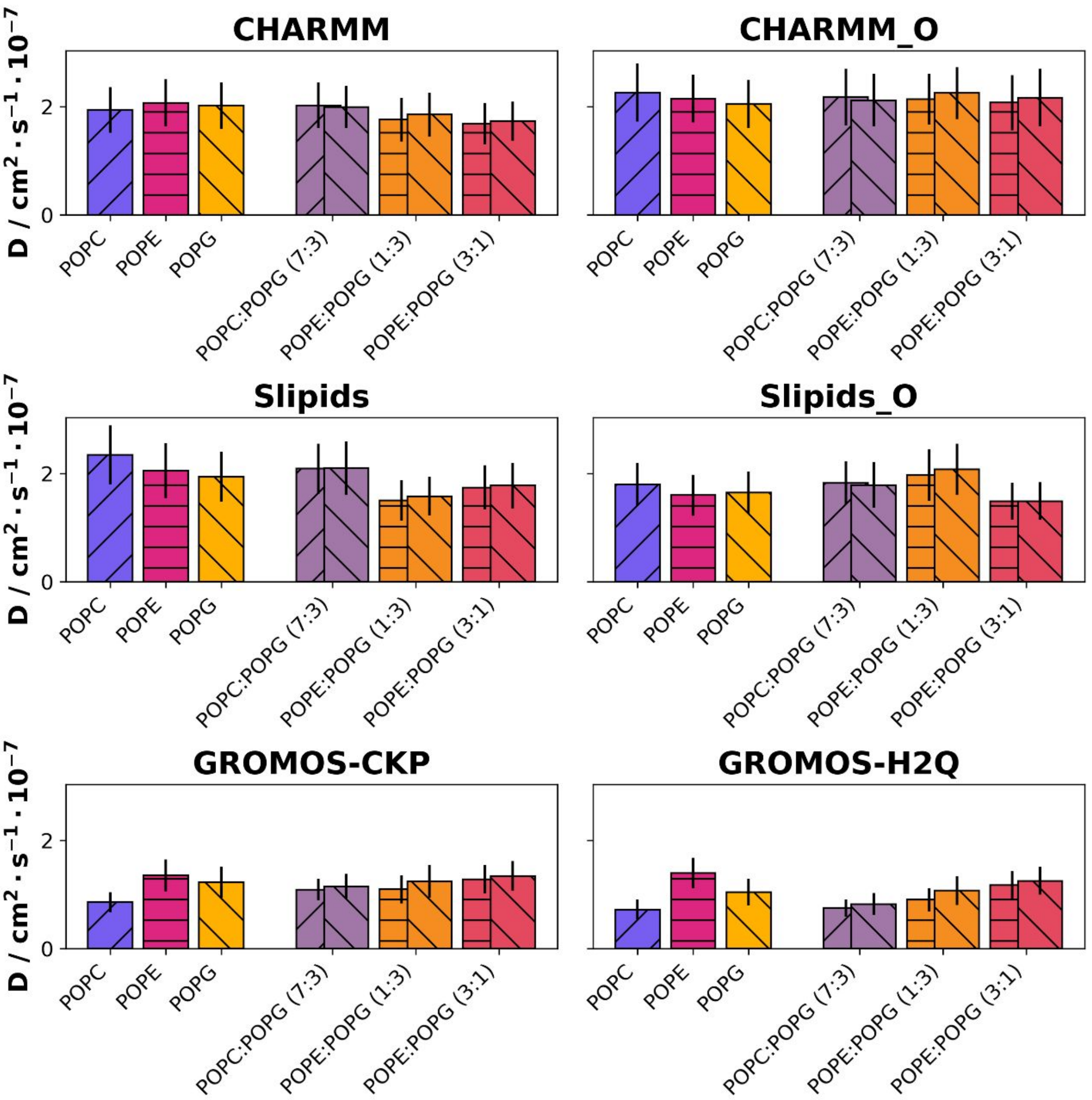

Figure S36. Bar plots from Figure S32, for a time window of t = 10 ns. The hatching represents the specific lipid type: forward slash – POPC, horizontal slash – POPE, backward slash – POPG. The errorbar represents ± σ.

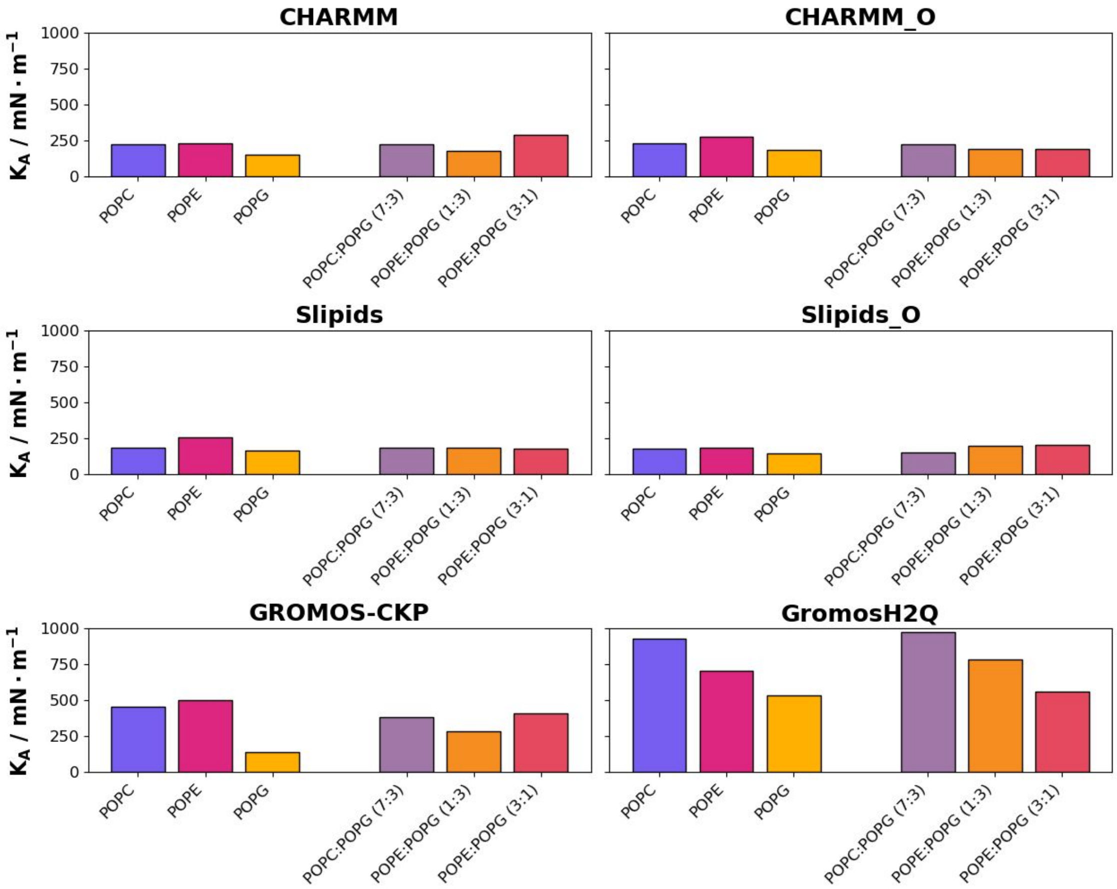

Figure S37. Area compressibility moduli obtained for the different force field parameterizations and lipid compositions.

Table S1. Area per lipid (A_L, in Å^2) calculated along the last 100 ns of the simulation (average and standard deviation). The last column corresponds to the experimental published values. n. a. (not available). (*) Non-very trustable value.

	CHARMM	CHARMM_O	Slipids	Slipids_O	GROMOS-CKP	GROMOS-H2Q	Experimental value
**POPC**	64.11±0.69	64.99±0.69	67.11±0.78	65.72±0.80	64.41±0.48	63.94±0.46	54.0 (275K)[4]<br>62.7 (293K)[5]<br>63.0 (297K)[6]<br>68.3 (303K)[7]<br>64.3 (303K)[5]<br>66.0 (310K)[8]<br>62.0 (323K)[4]
**POPE**	54.55±0.64	57.16±0.59	58.97±0.63	58.03±0.73	59.64±0.45	58.92±0.51	56.6 (303K)[9]
**POPG**	67.12±0.84	68.40±0.79	68.88±0.94	67.54±0.88	65.99±0.90	65.03±0.61	66.0 (303K)[9] *
**POPC:POPG (7:3)**	63.96±0.73	65.44±0.71	66.99±0.79	65.48±0.86	60.07±0.52	60.08±0.44	63.6 [9] *
**POPE:POPG (1:3)**	63.76±0.77	64.79±0.75	66.50±0.77	65.18±0.74	63.31±0.62	62.57±0.50	n. a.
**POPE:POPG (3:1)**	57.14±0.57	58.92±0.72	61.55±0.77	59.74±0.70	60.56±0.50	60.02±0.58	n. a.

Table S2. Bilayer thickness, calculated as a distance between average positions of phosphorus (P) atoms in the two leaflets of a bilayer (P-P distance). n. a. (not available).

	CHARMM	CHARMM_O	Slipids	Slipids_O	GROMOS-CKP	GROMOS-H2Q	Experimental value
**POPC**	38.81±4.22	38.66±4.05	37.14±4.17	37.29±3.94	37.49±3.85	37.84±3.86	39.1 (303K)5
**POPE**	42.19±3.60	41.50±3.68	39.12±3.84	39.96±3.60	38.99±3.61	39.84±3.78	40.5 (308K)10
**POPG**	37.23±4.55	36.94±4.75	35.77±4.41	35.80±4.39	37.21±4.15	37.51±4.21	36.3-38.5 (303K)[11]
**POPC:POPG (7:3)**	38.62±4.18	38.15±4.14	36.17±3.96	37.26±4.10	40.16±3.61	39.99±3.63	n. a.
**POPE:POPG (1:3)**	38.77±4.61	37.50±4.30	35.75±4.31	36.10±4.46	38.20±4.28	38.77±4.27	n. a.
**POPE:POPG (3:1)**	41.34±4.16	40.88±3.93	38.78±3.95	38.80±3.89	39.02±3.71	39.53±3.73	39.4 (309K)[12]

Table S3. Number of water molecules located within the bilayer's inner region. This inner region is specifically defined as the area situated between the glycerol lateral density peaks. The values were calculated by converting the lateral density from kg/m^3 to the number of water molecules along the Z-axis in #molecules/nm. Subsequently, the water curve was integrated along the Z-axis, but only within the region between the glycerol peaks.

	CHARMM	CHARMM_O	Slipids	Slipids_O	GROMOS-CKP	GROMOS-H2Q
**POPC**	1.46±0.13	1.34±0.13	1.28±0.17	1.13±0.11	0.660±0.074	0.65±0.12
**POPE**	0.786±0.068	0.868±0.078	0.77±0.11	0.654±0.050	0.418±0.062	0.404±0.042
**POPG**	1.43±0.16	1.56±0.23	1.11±0.12	1.02±0.16	0.72±0.10	0.70±0.15
**POPC:POPG (7:3)**	1.29±0.13	1.30±0.13	1.058±0.084	0.99±0.12	0.402±0.038	0.402±0.044
**POPE:POPG (1:3)**	1.32±0.16	1.30±0.25	1.01±0.15	1.01±0.15	0.59±0.12	0.59±0.14
**POPE:POPG (3:1)**	0.99±0.10	0.904±0.082	0.82±0.10	0.710±0.076	0.412±0.054	0.434±0.068

Table S4. Normalized number of H-bonds between the different groups (solvent, headgroups and glycerol) of each POPC and the POPC neighbors, along the last 100 ns of the trajectory of the simulation of the POPC membrane. All values are multiplied by 100.

	CHARMM	CHARMM_O	Slipids	Slipids_O	GROMOS-CKP	GROMOS-H2Q
**glc(PC)-Solv**	1.530±0.031	1.515±0.031	1.249±0.029	1.221±0.027	1.534±0.026	1.514±0.027
**hgp(PC)-Solv**	3.014±0.029	3.015±0.029	2.828±0.025	2.811±0.025	1.487±0.024	1.488±0.025

Table S5. Normalized number of H-bonds between the different groups (solvent, headgroups and glycerol) of each POPE and the POPE neighbors, along the last 100 ns of the trajectory of the simulation of the POPE membrane. All values are multiplied by 100.

	CHARMM	CHARMM_O	Slipids	Slipids_O	GROMOS-CKP	GROMOS-H2Q
**glc(PE)-Solv**	1.356±0.032	1.388±0.032	1.050±0.030	1.023±0.027	1.149±0.034	1.146±0.034
**hgp(PE)-glc(PE)**	1.50±0.14	1.38±0.13	1.47±0.14	1.51±0.13	6.08±0.21	6.07±0.19
**hgp(PE)-hgp(PE)**	4.76±0.18	4.67±0.20	5.25±0.18	5.36±0.21	0.80±0.10	0.80±0.10
**hgp(PE)-Solv**	3.532±0.051	3.591±0.055	3.361±0.048	3.327±0.051	2.687±0.041	2.707±0.039

Table S6. Normalized number of H-bonds between the different groups (solvent, headgroups and glycerol) of each POPG and the POPG neighbors, along the last 100 ns of the trajectory of the simulation of the POPG membrane. All values are multiplied by 100.

	**CHARMM**	**CHARMM_O**	**Slipids**	**Slipids_O**	**GROMOS-CKP**	**GROMOS-H2Q**
**glc(PG)-Solv**	1.527±0.033	1.521±0.031	1.049±0.031	1.011±0.032	1.203±0.037	1.187±0.037
**hgp(PG)-glc(PG)**	1.056±0.092	1.080±0.099	0.940±0.090	0.965±0.094	1.10±0.10	1.152±0.088
**hgp(PG)-hgp(PG)**	3.56±0.11	3.50±0.11	2.50±0.13	2.53±0.11	3.51±0.11	3.56±0.12
**hgp(PG)-Solv**	4.390±0.050	4.413±0.050	4.146±0.052	4.103±0.055	3.335±0.049	3.316±0.048

Table S7. Normalised number of H-bonds between the different groups (solvent, headgroups and glycerol) of each POPC and POPG and the POPC or POPG neighbors, along the last 100 ns of the trajectory of the simulation of the POPC:POPG (7:3) membrane. All values are multiplied by 100.

	**CHARMM**	**CHARMM_O**	**Slipids**	**Slipids_O**	**GROMOS-CKP**	**GROMOS-H2Q**
**glc(PC)-hgp(PG)**	0.397±0.094	0.393±0.095	0.40±0.10	0.404±0.097	0.70±0.10	0.77±0.11
**glc(PC)-Solv**	1.073±0.026	1.076±0.027	0.832±0.025	0.805±0.024	0.799±0.023	0.803±0.022
**glc(PG)-Solv**	0.458±0.018	0.456±0.017	0.343±0.016	0.331±0.016	0.399±0.016	0.379±0.016
**hgp(PC)-hgp(PG)**	0.58±0.11	0.56±0.11	0.56±0.12	0.58±0.13	1.15±0.13	1.14±0.13
**hgp(PC)-Solv**	2.164±0.026	2.170±0.027	1.976±0.024	1.968±0.025	1.070±0.021	1.079±0.023
**hgp(PG)-glc(PG)**	0.60±0.15	0.64±0.13	0.51±0.14	0.52±0.13	0.71±0.13	0.59±0.12
**hgp(PG)-hgp(PG)**	2.80±0.18	2.77±0.17	1.89±0.19	1.92±0.19	1.22±0.15	1.16±0.14
**hgp(PG)-Solv**	1.346±0.028	1.358±0.026	1.314±0.029	1.307±0.026	0.951±0.024	0.938±0.026

Table S8. Average number of H-bonds between the different groups (solvent, headgroups and glycerol) of each POPE and POPG and the POPE or POPG neighbors, along the last 100 ns of the trajectory of the simulation of the POPE:POPG (1:3) membrane. All values are multiplied by 100.

	CHARMM	CHARMM_O	Slipids	Slipids_O	GROMOS-CKP	GROMOS-H2Q
**glc(PE)-hgp(PG)**	0.226±0.062	0.221±0.061	0.242±0.063	0.269±0.065	0.365±0.081	0.351±0.071
**glc(PE)-Solv**	0.373±0.016	0.367±0.016	0.261±0.015	0.247±0.013	0.294±0.016	0.288±0.015
**glc(PG)-Solv**	1.133±0.027	1.125±0.029	0.798±0.026	0.777±0.028	0.865±0.034	0.864±0.031
**hgp(PE)-glc(PE)**	0.88±0.21	0.83±0.21	0.61±0.18	0.65±0.17	2.70±0.30	2.84±0.26
**hgp(PE)-glc(PG)**	0.52±0.13	0.51±0.12	0.60±0.13	0.58±0.13	3.16±0.25	3.23±0.19
**hgp(PE)-hgp(PE)**	1.56±0.25	1.67±0.28	1.31±0.22	1.22±0.23	0.51±0.15	0.54±0.15
**hgp(PE)-hgp(PG)**	1.60±0.14	1.62±0.15	2.21±0.14	2.33±0.15	1.72±0.14	1.55±0.14
**hgp(PE)-Solv**	0.987±0.026	0.982±0.025	0.895±0.025	0.890±0.024	0.579±0.021	0.580±0.019
**hgp(PG)-glc(PG)**	0.86±0.11	0.90±0.10	0.753±0.097	0.754±0.100	0.85±0.11	0.879±0.094
**hgp(PG)-hgp(PG)**	3.29±0.13	3.24±0.13	2.23±0.13	2.23±0.13	2.70±0.12	2.80±0.12
**hgp(PG)-Solv**	3.287±0.042	3.302±0.044	3.134±0.044	3.106±0.044	2.559±0.047	2.572±0.042

Table S9. Average number of H-bonds between the different groups (solvent, headgroups and glycerol) of each POPE and POPG and the POPE or POPG neighbors, along the last 100 ns of the trajectory of the simulation of the POPE:POPG (3:1) membrane. All values are multiplied by 100.

	CHARMM	CHARMM_O	Slipids	Slipids_O	GROMOS-CKP	GROMOS-H2Q
**glc(PE)-hgp(PG)**	0.336±0.093	0.313±0.083	0.380±0.098	0.404±0.099	0.378±0.091	0.466±0.098
**glc(PE)-Solv**	1.040±0.029	1.043±0.028	0.784±0.026	0.751±0.023	0.871±0.028	0.870±0.027
**glc(PG)-Solv**	0.372±0.017	0.373±0.016	0.271±0.014	0.263±0.016	0.271±0.015	0.278±0.017
**hgp(PE)-glc(PE)**	1.34±0.16	1.30±0.15	1.23±0.15	1.28±0.15	5.30±0.20	5.21±0.21
**hgp(PE)-glc(PG)**	0.50±0.10	0.45±0.11	0.49±0.11	0.48±0.11	2.67±0.15	2.68±0.18
**hgp(PE)-hgp(PE)**	3.70±0.21	3.73±0.19	3.88±0.19	4.02±0.21	0.70±0.10	0.69±0.11
**hgp(PE)-hgp(PG)**	2.20±0.20	2.18±0.20	2.90±0.21	3.01±0.22	2.10±0.20	2.12±0.17
**hgp(PE)-Solv**	2.731±0.045	2.739±0.042	2.590±0.042	2.544±0.039	1.933±0.033	1.934±0.034
**hgp(PG)-glc(PG)**	0.46±0.13	0.53±0.14	0.43±0.13	0.45±0.14	0.45±0.12	0.41±0.11
**hgp(PG)-hgp(PG)**	2.70±0.20	2.67±0.19	1.65±0.21	1.61±0.19	1.44±0.17	1.27±0.15
**hgp(PG)-Solv**	1.103±0.028	1.111±0.027	1.040±0.027	1.017±0.029	0.893±0.027	0.915±0.024

Table S10. Diffusion coefficient for each lipid type in 10^{-7} cm^2/s, calculated for the time window Δt = 2 ns.

	CHARMM	CHARMM_O	Slipids	Slipids_O	GROMOS-CPK	GROMOS-H2Q
**POPC**	3.95±0.41	4.28±0.45	3.99±0.41	3.60±0.40	1.57±0.26	1.28±0.24
**POPE**	3.36±0.37	3.94±0.47	3.11±0.39	2.81±0.42	2.01±0.41	1.73±0.36
**POPG**	3.78±0.43	4.06±0.44	3.36±0.44	3.12±0.41	1.97±0.36	1.77±0.34
**POPC:POPG (7:3)**	3.80±0.40, 3.74±0.39	4.14±0.43, 4.12±0.41	3.70±0.40, 3.51±0.39	3.31±0.38, 3.14±0.36	1.55±0.32, 1.71±0.41	1.32±0.24, 1.50±0.30
**POPE:POPG (1:3)**	3.28±0.45, 3.59±0.41	3.78±0.47, 4.06±0.46	2.82±0.38, 3.14±0.42	2.98±0.42, 3.28±0.44	1.52±0.31, 1.94±0.39	1.37±0.28, 1.73±0.34
**POPE:POPG (3:1)**	3.34±0.41, 3.62±0.46	3.77±0.48, 4.03±0.48	3.10±0.44, 3.25±0.42	2.86±0.41, 2.92±0.44	1.79±0.39, 1.97±0.37	1.63±0.36, 1.88±0.39

Table S11. Diffusion coefficient for each lipid type in 10^{-7} cm^2/s, calculated for the time window Δt = 5 ns.

	CHARMM	CHARMM_O	Slipids	Slipids_O	GROMOS-CPK	GROMOS-H2Q
**POPC**	2.62±0.38	2.88±0.48	2.85±0.47	2.46±0.38	1.09±0.21	0.90±0.18
**POPE**	2.44±0.36	2.74±0.42	2.44±0.41	1.96±0.34	1.67±0.33	1.38±0.28
**POPG**	2.60±0.40	2.67±0.41	2.45±0.43	2.15±0.37	1.43±0.29	1.30±0.26
**POPC:POPG (7:3)**	2.49±0.35, 2.45±0.34	2.77±0.42, 2.75±0.41	2.59±0.38, 2.51±0.41	2.34±0.38, 2.28±0.38	1.20±0.25, 1.31±0.29	1.01±0.18, 1.11±0.23
**POPE:POPG (1:3)**	2.25±0.37, 2.40±0.37	2.64±0.42, 2.76±0.41	1.94±0.34, 2.08±0.36	2.35±0.39, 2.51±0.43	1.19±0.25, 1.40±0.30	1.03±0.23, 1.25±0.27
**POPE:POPG (3:1)**	2.21±0.35, 2.31±0.36	2.67±0.50, 2.81±0.46	2.20±0.41, 2.29±0.39	1.96±0.35, 1.97±0.39	1.50±0.30, 1.55±0.26	1.32±0.27, 1.44±0.27

Table S12. Diffusion coefficient for each lipid type in 10^{-7} cm^2/s, calculated for the time window Δt = 10 ns.

	**CHARMM**	**CHARMM_O**	**Slipids**	**Slipids_O**	**GROMOS-CPK**	**GROMOS-H2Q**
**POPC**	1.94±0.42	2.26±0.54	2.35±0.55	1.80±0.39	0.86±0.18	0.72±0.18
**POPE**	2.07±0.43	2.15±0.44	2.06±0.51	1.60±0.38	1.36±0.30	1.40±0.28
**POPG**	2.02±0.43	2.05±0.44	1.94±0.46	1.65±0.39	1.23±0.29	1.04±0.25
**POPC:POPG (7:3)**	2.03±0.42, 2.00±0.39	2.18±0.52, 2.12±0.49	2.09±0.46, 2.10±0.50	1.83±0.40, 1.79±0.42	1.09±0.19, 1.15±0.23	0.75±0.16, 0.82±0.21
**POPE:POPG (1:3)**	1.76±0.40, 1.86±0.41	2.14±0.47, 2.25±0.48	1.50±0.38, 1.58±0.36	1.97±0.48, 2.08±0.47	1.10±0.26, 1.24±0.30	0.90±0.22, 1.07±0.27
**POPE:POPG (3:1)**	1.69±0.38, 1.73±0.36	2.07±0.51, 2.17±0.53	1.74±0.41, 1.78±0.42	1.49±0.34, 1.49±0.35	1.28±0.27, 1.34±0.27	1.17±0.26, 1.25±0.26

References:

[1] T.M. Ferreira, F. Coreta-Gomes, O.H. Samuli Ollila, M.J. Moreno, W.L.C. Vaz, D. Topgaard, Cholesterol and POPC segmental order parameters in lipid membranes: Solid state 1H-13C NMR and MD simulation studies, Phys. Chem. Chem. Phys. 15 (2013) 1976–1989. https://doi.org/10.1039/c2cp42738a.

[2] A. Wurl, K. Saalwächter, T. Mendes Ferreira, Time-domain proton-detected local-field NMR for molecular structure determination in complex lipid membranes, Magn. Reson. 4 (2023) 115–127. https://doi.org/10.5194/mr-4-115-2023.

[3] A. Bacle, P. Buslaev, R. Garcia-Fandino, F. Favela-Rosales, T. Mendes Ferreira, P.F.J. Fuchs, I. Gushchin, M. Javanainen, A.M. Kiirikki, J.J. Madsen, J. Melcr, P. Milán Rodríguez, M.S. Miettinen, O.H.S. Ollila, C.G. Papadopoulos, A. Peón, T.J. Piggot, Á. Piñeiro, S.I. Virtanen, Inverse Conformational Selection in Lipid-Protein Binding, J. Am. Chem. Soc. 143 (2021) 13701–13709. https://doi.org/10.1021/jacs.1c05549.

[4] G. Pabst, M. Rappolt, H. Amenitsch, P. Laggner, Structural information from multilamellar liposomes at full hydration: Full q-range fitting with high quality X-ray data, Phys. Rev. E - Stat. Physics, Plasmas, Fluids, Relat. Interdiscip. Top. 62 (2000) 4000–4009. https://doi.org/10.1103/PhysRevE.62.4000.

[5] N. Kučerka, M.P. Nieh, J. Katsaras, Fluid phase lipid areas and bilayer thicknesses of commonly used phosphatidylcholines as a function of temperature, Biochim. Biophys. Acta - Biomembr. 1808 (2011) 2761–2771. https://doi.org/10.1016/j.bbamem.2011.07.022.

[6] J.M. Smaby, M.M. Momsen, H.L. Brockman, R.E. Brown, Phosphatidylcholine acyl unsaturation modulates the decrease in interfacial elasticity induced by cholosterol, Biophys. J. 73 (1997) 1492–1505. https://doi.org/10.1016/S0006-3495(97)78181-5.

[7] N. Kučerka, S. Tristram-Nagle, J.F. Nagle, Structure of fully hydrated fluid phase lipid bilayers with monounsaturated chains, J. Membr. Biol. 208 (2006) 193–202. https://doi.org/10.1007/s00232-005-7006-8.

[8] P.A. Hyslop, B. Morel, R.D. Sauerheber, Organization and Interaction of Cholesterol and Phosphatidylcholine in Model Bilayer Membranes, Biochemistry. 29 (1990) 1025–1038. https://doi.org/10.1021/bi00456a027.

[9] S. Mukherjee, R.K. Kar, R.P.R. Nanga, K.H. Mroue, A. Ramamoorthy, A. Bhunia, Accelerated molecular dynamics simulation analysis of MSI-594 in a lipid bilayer, Phys. Chem. Chem. Phys. 19 (2017) 19289–19299. https://doi.org/10.1039/c7cp01941f.

[10] N. Kučerka, B. Van Oosten, J. Pan, F.A. Heberle, T.A. Harroun, J. Katsaras, Molecular structures of fluid phosphatidylethanolamine bilayers obtained from simulation-to-experiment comparisons and experimental scattering density profiles, J. Phys. Chem. A. 119 (2015) 1947–1956. https://doi.org/10.1021/jp511159q.

[11] K. N, H. BW, G. CG, T. B, K. J, Scattering density profile model of POPG bilayers as determined by molecular dynamics simulations and small-angle neutron and X-ray scattering experiments, J. Phys. Chem. B. 116 (2012) 232–239. https://doi.org/10.1021/JP208920H.

[12] T. Silva, B. Claro, B.F.B. Silva, N. Vale, P. Gomes, M.S. Gomes, S.S. Funari, J. Teixeira, D. Uhríková, M. Bastos, Unravelling a Mechanism of Action for a Cecropin A-Melittin Hybrid Antimicrobial Peptide: The Induced Formation of Multilamellar Lipid Stacks, Langmuir. 34 (2018) 2158–2170. https://doi.org/10.1021/ACS.LANGMUIR.7B03639.
